# Cyclic AMP Recruits a Discrete Intracellular Ca^2+^ Store by Unmasking Hypersensitive IP_3_ Receptors

**DOI:** 10.1016/j.celrep.2016.12.058

**Published:** 2017-01-17

**Authors:** Vera Konieczny, Stephen C. Tovey, Stefania Mataragka, David L. Prole, Colin W. Taylor

**Affiliations:** 1 Department of Pharmacology, University of Cambridge, Tennis Court Road, Cambridge CB2 1PD, UK

**Keywords:** cyclic AMP, endoplasmic reticulum, inositol 1,4,5-trisphosphate, intracellular Ca^2+^ store, IRBIT, Golgi apparatus, parathyroid hormone, phospholipase C

## Abstract

Inositol 1,4,5-trisphosphate (IP_3_) stimulates Ca^2+^ release from the endoplasmic reticulum (ER), and the response is potentiated by 3′,5′-cyclic AMP (cAMP). We investigated this interaction in HEK293 cells using carbachol and parathyroid hormone (PTH) to stimulate formation of IP_3_ and cAMP, respectively. PTH alone had no effect on the cytosolic Ca^2+^ concentration, but it potentiated the Ca^2+^ signals evoked by carbachol. Surprisingly, however, the intracellular Ca^2+^ stores that respond to carbachol alone could be both emptied and refilled without affecting the subsequent response to PTH. We provide evidence that PTH unmasks high-affinity IP_3_ receptors within a discrete Ca^2+^ store. We conclude that Ca^2+^ stores within the ER that dynamically exchange Ca^2+^ with the cytosol maintain a functional independence that allows one store to be released by carbachol and another to be released by carbachol with PTH. Compartmentalization of ER Ca^2+^ stores adds versatility to IP_3_-evoked Ca^2+^ signals.

## Highlights

• Cyclic AMP directly potentiates IP_3_-evoked Ca^2+^ release
• The Ca^2+^ stores released by IP_3_ alone or IP_3_ with cAMP are functionally independent
• Cyclic AMP unmasks high-affinity IP_3_ receptors in a discrete ER Ca^2+^ store
• Independent regulation of discrete Ca^2+^ stores increases signaling versatility

## Graphical Abstract

**Figure:**
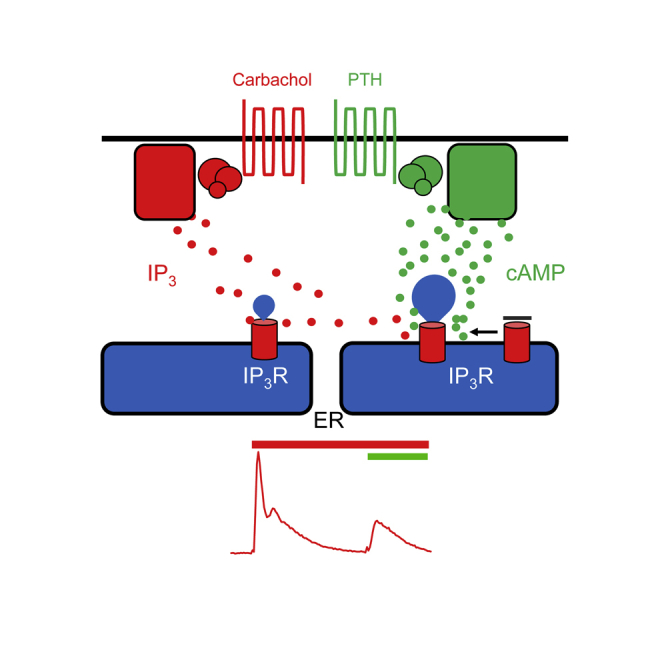

## Teaser

Cyclic AMP directly potentiates IP_3_-evoked Ca^2+^ release. Konieczny et al. find that IP_3_ alone and IP_3_ with cAMP release Ca^2+^ from independent stores within the endoplasmic reticulum. Compartmentalized Ca^2+^ stores increase the versatility of IP_3_-mediated Ca^2+^ signaling.

## Introduction

**Figure 1:**
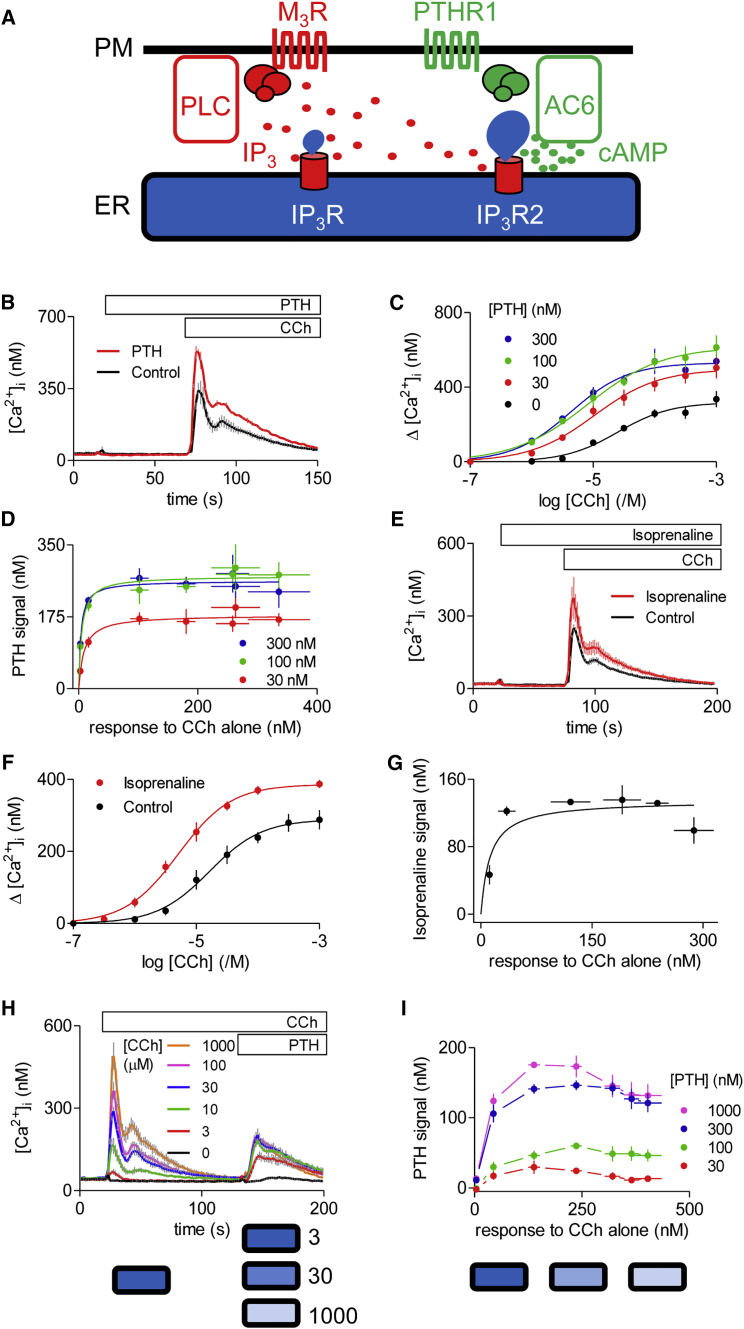
Potentiation of CCh-Evoked Ca^2+^ Signals by PTH and Isoprenaline (A) CCh through type 3 muscarinic acetylcholine receptors (M_3_Rs) stimulates phospholipase C (PLC) and formation of IP_3_, which stimulates Ca^2+^ release from the ER through IP_3_Rs. Stimulation of PTHR1 with PTH activates adenylyl cyclase (AC). The cAMP produced potentiates the Ca^2+^ release evoked by IP_3_. A specific association of AC6 with IP_3_R2 allows cAMP to be delivered at high concentrations to IP_3_Rs. (B) Typical results (mean ± SD from three wells) for HEK-PR1 cells stimulated with PTH (100 nM) and then CCh (1 mM) in Ca^2+^-free HBS. (C) Summary results show concentration-dependent effects of CCh alone or after pre-incubation (1 min) with the indicated concentrations of PTH on the increase in [Ca^2+^]_i_ (Δ[Ca^2+^]_i_). (D) From the results shown in (C), the increase in [Ca^2+^]_i_ evoked by each CCh concentration alone was subtracted from the response evoked by the same CCh concentration with PTH. The [Ca^2+^]_i_ increase due to PTH is plotted against that evoked by CCh alone. (E) Typical results (mean ± SD from three wells) for HEK293 cells stimulated with isoprenaline (10 μM) and then CCh (1 mM) in Ca^2+^-free HBS. (F) Summary results show the concentration-dependent effects on Δ[Ca^2+^]_i_ of CCh alone or after pre-incubation (1 min) with isoprenaline (10 μM). (G) Δ[Ca^2+^]_i_ due to isoprenaline is plotted against that evoked by CCh alone. (H) Typical responses (mean ± SD from three wells) for HEK-PR1 cells stimulated with the indicated concentrations of CCh before addition of PTH (300 nM). (I) Summary results show the responses evoked by PTH plotted against the increase in [Ca^2+^]_i_ evoked by the prior stimulation with CCh. Bottom diagrams in (H) and (I) represent the global Ca^2+^ content of the ER at the time of stimulus addition (darker tones indicate fuller stores, and the numbers alongside represent CCh concentrations in μM). Results are means ± SEM, n ≥ 3 (C, D, F, G, and I). See also Figures S2 and S3.

G-protein-coupled receptors (GPCRs) comprise the largest class of cell-surface receptors, and they endow cells with the ability to respond to diverse extracellular stimuli. However, most signaling from GPCRs proceeds through a very small number of intracellular messengers, among which 3′,5′-cyclic AMP (cAMP) and Ca^2+^ are the most prominent. GPCRs evoke cAMP formation by stimulating adenylyl cyclases (ACs), whereas most GPCR-evoked Ca^2+^ signals result from stimulation of phospholipase C (PLC) and formation of inositol 1,4,5-trisphosphate (IP_3_). IP_3_ then evokes Ca^2+^ release from the endoplasmic reticulum (ER) through IP_3_ receptors (IP_3_Rs) (Figure 1A) (Foskett et al., 2007, Prole and Taylor, 2016). At least three features contribute to specificity within these convergent GPCR signaling pathways. First, individual cells express only a few of the hundreds of GPCRs encoded by the human genome. Most cells are therefore insensitive to most stimuli that activate GPCRs. Second, regulation of many of the signaling proteins, notably ACs and IP_3_Rs, is polymodal. The proteins therefore respond optimally only when combinations of stimuli are presented together (Prole and Taylor, 2016, Willoughby and Cooper, 2007). Finally, signaling pathways are spatially organized, often with the aid of scaffold proteins, to allow targeted delivery of diffusible messengers to specific subcellular locations (Delmas et al., 2002, Konieczny et al., 2012, Tu et al., 1998, Willoughby and Cooper, 2007).

IP_3_Rs can be phosphorylated by cAMP-dependent protein kinase (PKA) and, at least for IP_3_R1 and IP_3_R2, this increases their IP_3_ sensitivity (Betzenhauser and Yule, 2010, Masuda et al., 2010). We and others have shown that cAMP can also potentiate IP_3_-evoked Ca^2+^ signals by a mechanism that requires neither of the usual targets of cAMP, PKA and exchange proteins activated by cAMP (EPACs) (Figure 1A) (Kurian et al., 2009, Tovey et al., 2008, Tovey et al., 2010). This potentiation is due to enhanced Ca^2+^ release by IP_3_Rs, rather than to inhibition of Ca^2+^ removal from the cytosol (Tovey et al., 2003). We have provided evidence that cAMP is delivered directly to IP_3_Rs within junctions formed between IP_3_R2 and AC6, and that within these junctions the local concentration of cAMP is more than sufficient to fully potentiate responses to IP_3_ (Figure 1A) (Tovey et al., 2008). We proposed that each junction works as a digital “on-off switch,” with more switches flicked as more AC-coupled receptors are activated (Tovey et al., 2008).

In the present study, we show that cAMP unmasks IP_3_Rs within an ER Ca^2+^ store that is functionally distinct from the store released by IP_3_ alone. Our results suggest a remarkable independence of the ER Ca^2+^ stores released by IP_3_ alone or IP_3_ combined with cAMP, and they thereby reveal an additional source of versatility within these signaling pathways.

## Results and Discussion

### Ca^2+^ Signals Evoked by Stimuli that Cause Very Different Increases in Intracellular Free Ca^2+^ Concentration Are Uniformly Enhanced by PTH

In Ca^2+^-free HEPES-buffered saline (HBS), carbachol (CCh) evoked a concentration-dependent increase in [Ca^2+^]_i_ (intracellular free Ca^2+^ concentration) (pEC_50_ = 4.60 ± 0.07, where pEC_50_ = −log of the half-maximally effective concentration) in HEK cells stably expressing type 1 human parathyroid hormone (PTH) receptor (HEK-PR1 cells) (Figures 1B and 1C). This is consistent with evidence that the endogenous M_3_ muscarinic acetylcholine receptors (M_3_R) of HEK293 cells stimulate Ca^2+^ release from intracellular stores through IP_3_Rs (Tovey et al., 2008). Neither isoprenaline, which stimulates endogenous β_2_-adrenoceptors, nor PTH evoked an increase in [Ca^2+^]_i_. However, pre-treatment with PTH or isoprenaline potentiated the increase in [Ca^2+^]_i_ evoked by maximal and submaximal concentrations of CCh (Figures 1B–1G). These results are consistent with previous reports showing that cAMP potentiates IP_3_-evoked Ca^2+^ signals in HEK293 cells (Kurian et al., 2009, Meena et al., 2015, Tovey et al., 2008) (Figure 1A).

Figure 1D compares the amplitudes of the Ca^2+^ signals evoked by CCh alone with the amplitude of the additional increase in [Ca^2+^]_i_ because of pre-treatment with a maximal (100 nM) or submaximal (30 nM) concentration of PTH. The results demonstrate that for CCh concentrations that evoked Ca^2+^ signals of very different amplitudes (∼20–340 nM), the additional increase in [Ca^2+^]_i_ evoked by PTH was almost invariant, but larger for the maximal concentration of PTH (Δ[Ca^2+^]_i_ ∼240 nM) than for the submaximal PTH concentration (Δ[Ca^2+^]_i_ ∼170 nM) (Figure 1D). Similar results were obtained when the cells were first stimulated with CCh and then with PTH after [Ca^2+^]_i_ had returned to its basal level (Figures 1H and 1I). The reduced sensitivity to PTH in this second protocol is probably due to the briefer exposure to PTH, which is likely to equilibrate slowly with its receptors.

**Figure 2:**
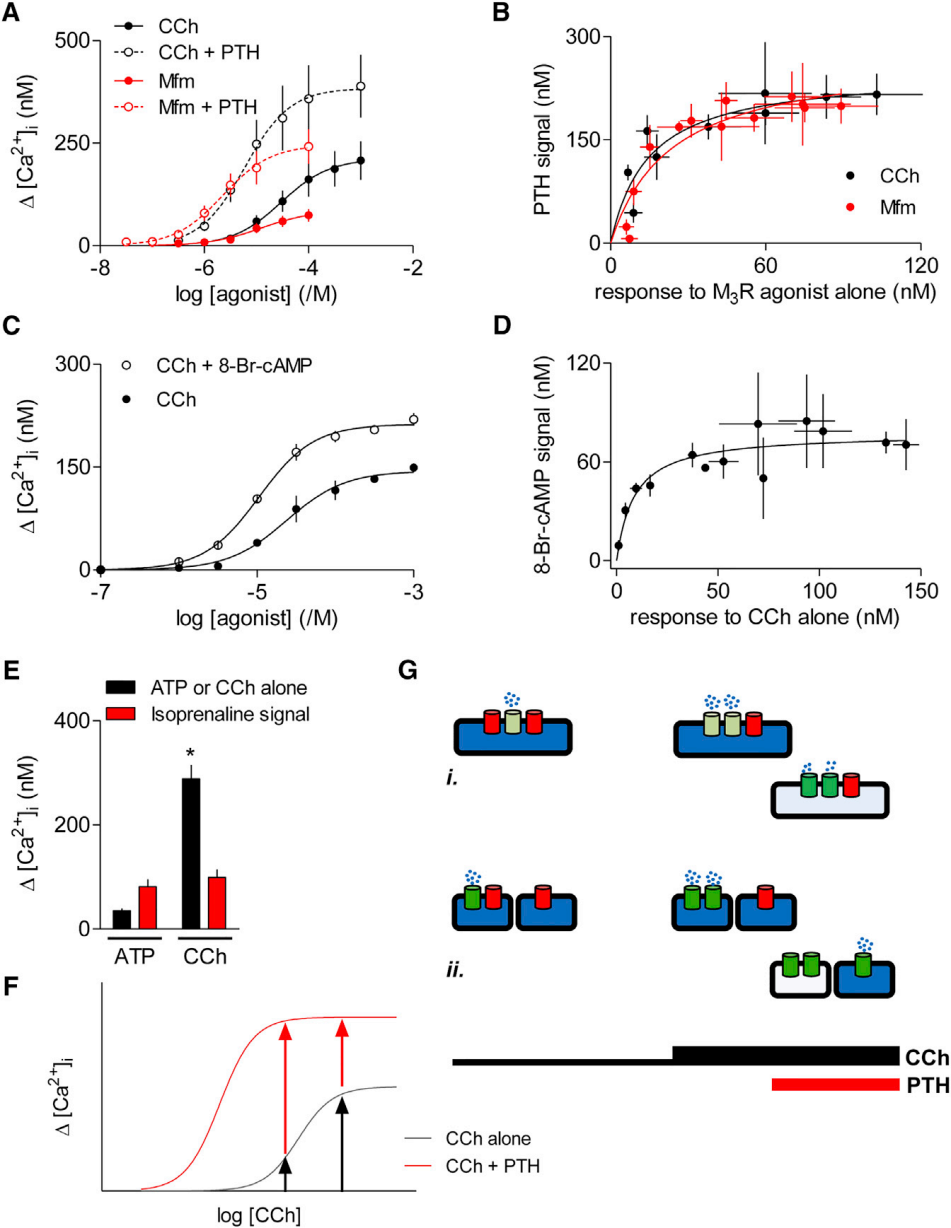
cAMP Evokes Similar Ca^2+^ Signals after Stimuli That Alone Evoke Very Different Increases in [Ca^2+^]_i_ (A) Methods similar to those shown in Figure 1B were used to assess the effects on [Ca^2+^]_i_ of the indicated concentrations of CCh or Mfm alone, or after pre-incubation with PTH (100 nM, 1 min). (B) Δ[Ca^2+^]_i_ due to PTH is plotted against that evoked by CCh or Mfm alone. (C) Effects on Δ[Ca^2+^]_i_ in HEK-PR1 cells of CCh alone or CCh after pre-incubation with 8-Br-cAMP (10 mM, 20 min). (D) Δ[Ca^2+^]_i_ due to 8-Br-cAMP is plotted against that evoked by CCh alone. (E) Similar analyses of HEK293 cells stimulated with ATP (300 μM) or CCh (1 mM) alone, or after pre-incubation with isoprenaline (10 μM, 1 min). The maximal amplitudes of the Ca^2+^ signals evoked by CCh or ATP alone, and the additional effect of isoprenaline are shown as means ± SEM, n ≥ 3. ^∗^p < 0.05, Student’s t test, for CCh compared with ATP. (F) Expected effects of PTH on CCh-evoked Ca^2+^ signals assuming that IP_3_ is uniformly delivered to all IP_3_Rs made more sensitive to IP_3_ by cAMP. Previous work established that even maximal activation of M_3_Rs in HEK-PR1 cells generates insufficient IP_3_ to activate all IP_3_Rs (Tovey et al., 2008), hence the increased maximal response to CCh in the presence of PTH. (G) The similar Ca^2+^ signals evoked by CCh with PTH after CCh alone has evoked Ca^2+^ signals with very different amplitudes might be because of Ca^2+^ release from a uniform ER, with the increased sensitivity of more IP_3_Rs compensating for the diminished ER Ca^2+^ content (i). Alternatively, CCh alone and CCh with PTH may evoke Ca^2+^ release through IP_3_Rs resident in different stores (ii). See also Figures S2 and S3.

5-Methylfurmethiodide (Mfm) is a partial agonist of M_3_Rs: the maximal increase in [Ca^2+^]_i_ evoked by Mfm was only 36% ± 1% of that evoked by CCh (Figure 2A). Nevertheless, the amplitude of the additional Ca^2+^ signal evoked in the presence of PTH was similar across most concentrations of Mfm, and also similar to that evoked by PTH with CCh (Figures 2A and 2B). Similar results were obtained when cells were pretreated with 8-bromo cAMP (8-Br-cAMP), rather than PTH, and then stimulated with CCh (Figures 2C and 2D). In HEK293 cells, ATP through P2Y receptors also evoked an increase in [Ca^2+^]_i_, but the maximal amplitude of the Ca^2+^ signal was only 13% ± 2% of that evoked by CCh in the same cells (Figure 2E). Nevertheless, the additional Ca^2+^ signal evoked by isoprenaline was similar for maximally effective concentrations of CCh and ATP (Figure 2E).

The similar effect of PTH (and of other cAMP-elevating stimuli) across most CCh concentrations (Figure 1) is unexpected because if PTH uniformly increased the sensitivity of IP_3_Rs to IP_3_, its effects should be most pronounced at the lowest CCh concentrations (Figure 2F). The same argument applies to the results with other submaximal responses to Ca^2+^-mobilizing stimuli (Figures 2A–2E). It might be argued that the similar effect of a maximal PTH concentration on the Ca^2+^ signals evoked by most CCh concentrations is due to PTH causing an increase in IP_3_R sensitivity sufficient for each CCh concentration to evoke maximal Ca^2+^ release. However, that explanation cannot easily be reconciled with the observation that the response to PTH is similar after stores have been minimally or substantially depleted of Ca^2+^ by prior treatment with CCh (bottom diagrams in Figures 1H and 1I). Nor could it account for the uniform effect of a submaximal PTH concentration, which also had similar effects across most CCh concentrations, although less than those of the maximal PTH concentration (Figures 1D and 1I).

These results demonstrate that for stimuli that evoke very different increases in [Ca^2+^]_i_, the additional Ca^2+^ release evoked by PTH (or isoprenaline) is similar. We can envisage two possible explanations for these observations. It may be that all stimuli release Ca^2+^ from a shared Ca^2+^ store, and the consistent responses to PTH then reflect a balance, as the CCh concentration increases, between the declining content of the Ca^2+^ store and a compensating increase in the sensitivity of a larger number of IP_3_Rs (Figure 2Gi). That fortuitous balance would need to hold across a diverse array of stimulus combinations and intensities, and between cell lines (Figures 1 and 2). Alternatively, CCh alone and CCh with PTH may release Ca^2+^ from different intracellular stores (Figure 2Gii). Subsequent experiments seek to distinguish between these possibilities.

### Depletion of the CCh-Sensitive Ca^2+^ Stores Does Not Affect Responses to PTH

**Figure 3:**
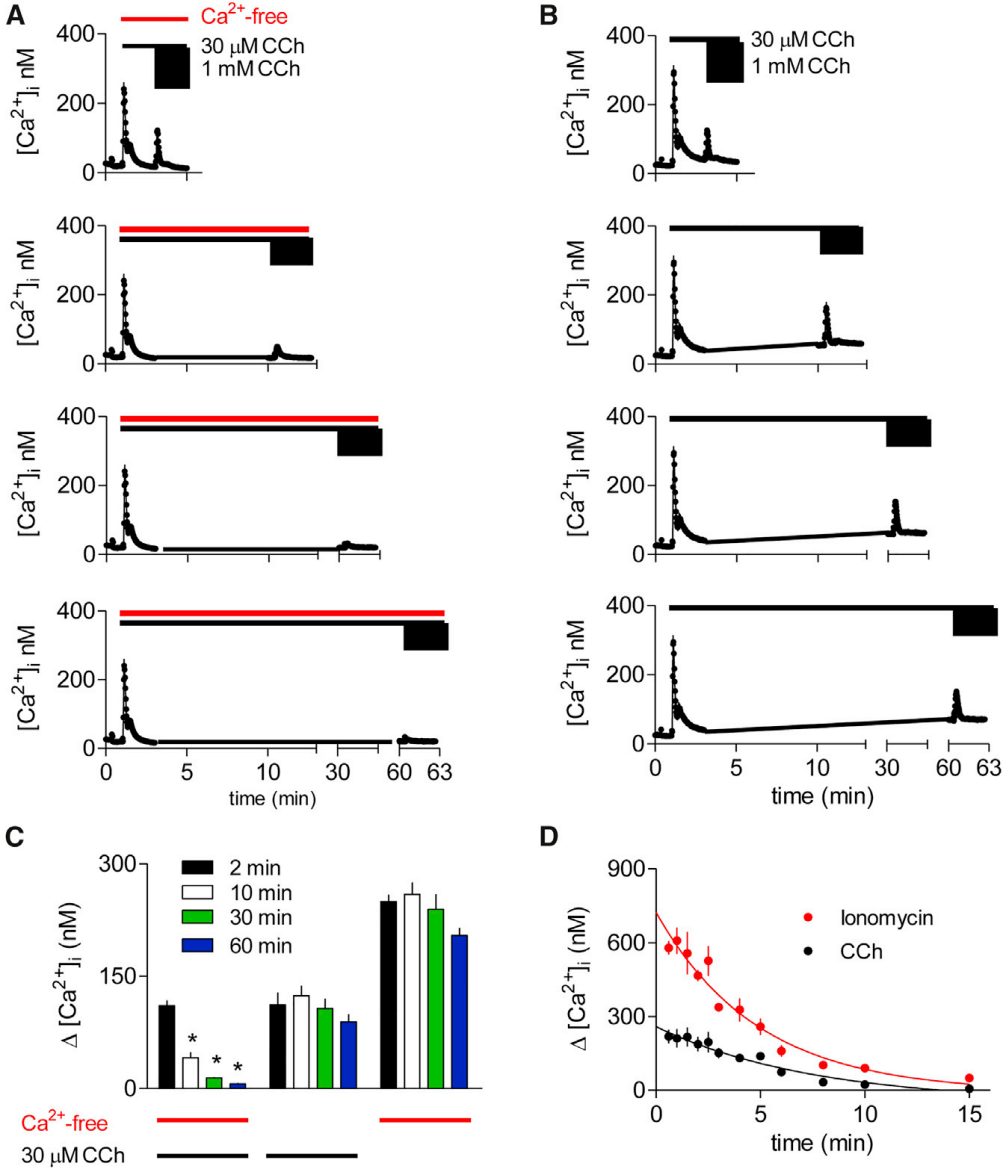
Depletion of the CCh-Sensitive Ca^2+^ Stores by Sustained Submaximal Stimulation with CCh (A and B) Populations of HEK-PR1 cells in either Ca^2+^-free HBS (A) or normal HBS (B) were stimulated with 30 μM CCh for the indicated times before addition of 1 mM CCh. Typical results show means ± SD from three replicates in each trace. (C) Summary results (mean ± SEM, from three independent experiments) show peak increases in [Ca^2+^]_i_ evoked by 1 mM CCh after pre-incubation with 30 μM CCh in Ca^2+^-free or Ca^2+^-containing HBS, or without prior stimulation with CCh in Ca^2+^-free HBS for the indicated times. ^∗^p < 0.05, one-way ANOVA and Tukey’s post hoc test, relative to measurements at 2 min. (D) HEK-PR1 cells in Ca^2+^-free HBS were stimulated with thapsigargin (1 μM), and at intervals thereafter the effects of CCh (1 mM) or ionomycin (10 μM) were determined. Results are mean ± SEM from three independent experiments. See also Figures S2 and S3.

During prolonged incubation of HEK-PR1 cells with a half-maximally effective concentration of CCh (30 μM) in Ca^2+^-free HBS, the increase in [Ca^2+^]_i_ evoked by subsequent addition of a maximal CCh concentration (1 mM) decreased with time (half-time for loss of response, t_½_ = 5 ± 1 min) (Figure 3A). After a 60 min incubation with 30 μM CCh, the response to maximal stimulation declined to 6% ± 1% of that recorded after a 2 min incubation. However, after a 60 min incubation in Ca^2+^-free HBS without CCh, the response to 1 mM CCh was reduced to 82% ± 4% of the initial response, and after a 60 min incubation with 30 μM CCh in Ca^2+^-containing HBS, the response to subsequent addition of 1 mM CCh was 83% ± 13% of the initial response (Figures 3B and 3C).

We considered whether the response to stimulation with a maximal concentration of CCh might fail to directly report the Ca^2+^ content of the CCh-sensitive stores. If, for example, IP_3_Rs were regulated by luminal Ca^2+^, then CCh-evoked Ca^2+^ release might terminate before the stores were empty. However, when cells were treated with 1 μM thapsigargin to inhibit the sarcoplasmic/endoplasmic reticulum Ca^2+^-ATPase (SERCA) and so unmask a Ca^2+^ leak from the ER, the rates of decline of the response to 1 mM CCh (t_½_ = 3.8 ± 0.4 min, n = 3) and of the Ca^2+^ content of the stores assessed by addition of 1 μM ionomycin (t_½_ = 3.0 ± 0.3 min) were indistinguishable (Figure 3D). Together these results demonstrate that sustained stimulation with a submaximal concentration of CCh depletes the intracellular stores from which CCh releases Ca^2+^.

**Figure 4:**
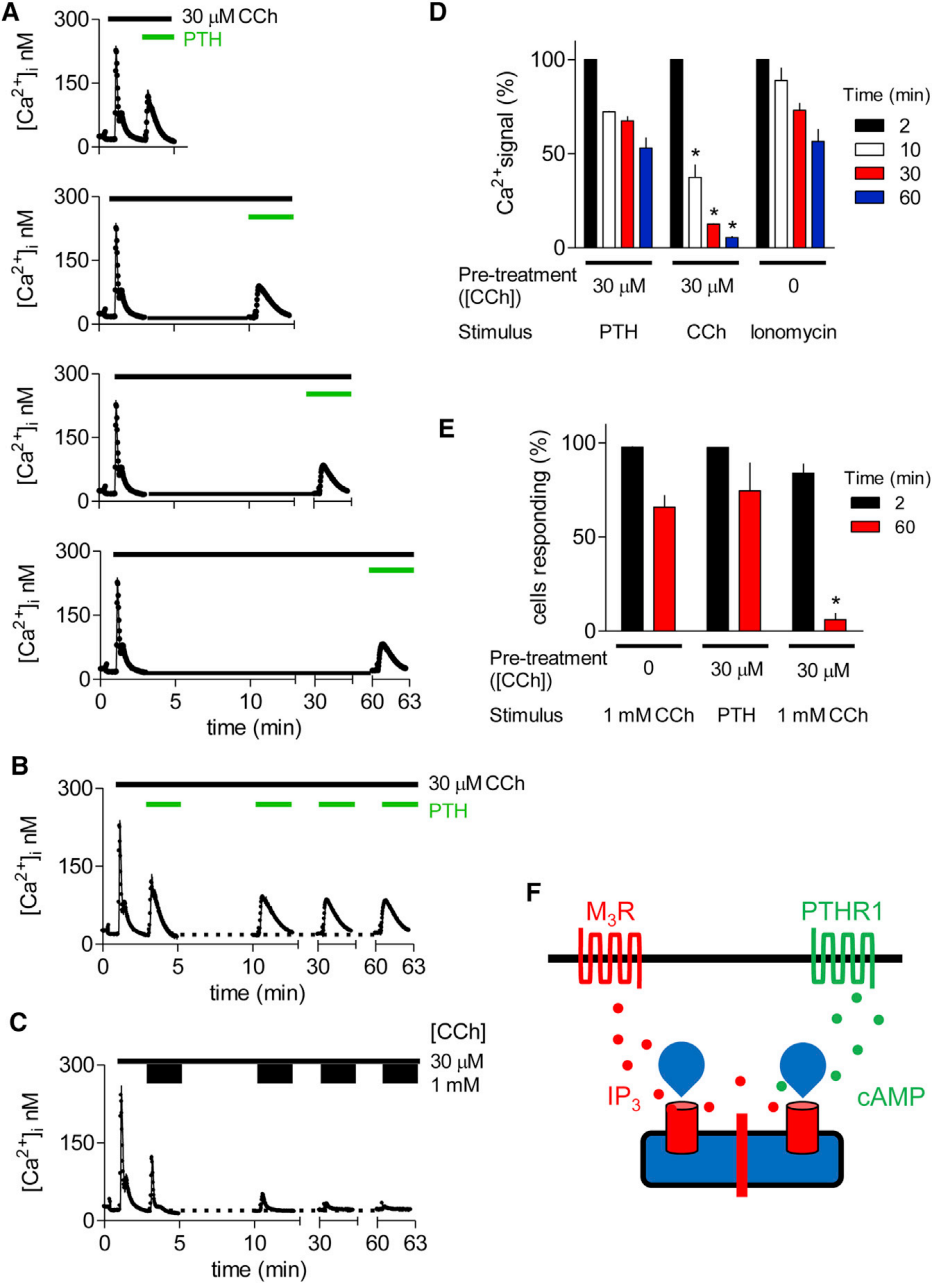
PTH Evokes Ca^2+^ Release after Depletion of CCh-Sensitive Ca^2+^ Stores (A) HEK-PR1 cells in Ca^2+^-free HBS were stimulated with 30 μM CCh for the indicated times before addition of 100 nM PTH. Typical results show means ± SD from three replicates in each trace. (B) Traces from the four experiments shown in (A) are reproduced in this single panel. (C) Similar representation of the results from Figure 3A. (D) Summary shows the peak increases in [Ca^2+^]_i_ evoked by 1 mM CCh or 100 nM PTH after the indicated periods in Ca^2+^-free HBS with 30 μM CCh, and the responses to 1 μM ionomycin after the indicated periods in Ca^2+^-free HBS alone. Results (mean ± SEM, n = 3) are normalized to the Ca^2+^ signals measured after 2 min. ^∗^p < 0.05, one-way ANOVA and Tukey’s post hoc test, relative to time-matched response to ionomycin. (E) Single HEK-PR1 cells were stimulated continuously with 30 μM CCh in Ca^2+^-free HBS and then with PTH (100 nM) or CCh (1 mM) after the indicated intervals. Results (mean ± SEM, from three experiments with at least 53 cells analyzed in each) show the percentage of responsive cells. ^∗^p < 0.05, one-way ANOVA and Tukey’s post hoc test, relative to measurement at 2 min. (F) The results suggest that CCh and CCh with PTH evoke Ca^2+^ release from independent Ca^2+^ stores. See also Figures S1–S3.

Addition of PTH (100 nM) to HEK-PR1 cells stimulated for 2 min with 30 μM CCh in Ca^2+^-free HBS evoked an increase in [Ca^2+^]_i_ (Δ[Ca^2+^]_i_ = 92 ± 10 nM) similar to that evoked by addition of 1 mM CCh (110 ± 7 nM) (Figures 4A–4C). However, whereas sustained stimulation with 30 μM CCh effectively abolished the response to subsequent addition of 1 mM CCh, it had very little effect on the response to PTH (Figures 4B and 4C). The modest decline in the response to PTH matched the slow decline of the Ca^2+^ content of the stores in Ca^2+^-free HBS without CCh (determined by addition of ionomycin; Figure 4D). Similar results, namely loss of the response to a maximal concentration of CCh alone and unperturbed responses to PTH, were observed when areas under the Ca^2+^ responses, rather than peak increases in [Ca^2+^]_i_, were analyzed (data not shown). Analysis of single HEK-PR1 cells using the same protocol established that the very different effects of depleting CCh-sensitive Ca^2+^ stores on subsequent responses to CCh or PTH were not due to cellular heterogeneity (Figure 4E).

PTH can, particularly when its receptors are overexpressed, stimulate formation of IP_3_ (He et al., 2015, Taylor and Tovey, 2012). However, we showed previously that PTH does not stimulate IP_3_ formation in HEK-PR1 cells (Meena et al., 2015, Short and Taylor, 2000), and others have shown that potentiation of M_3_R-evoked Ca^2+^ signals by activation of β_2_-adrenoceptors occurs without formation of additional IP_3_ (Kurian et al., 2009). Our conclusion that the effects of PTH are not mediated by formation of additional IP_3_ is further confirmed by the present results showing that PTH evokes Ca^2+^ release under conditions where increasing IP_3_ formation, by increasing the CCh concentration, is ineffective (Figures 4B and 4C).

Hence, although PTH evokes Ca^2+^ release only when there is coincident activation of M_3_Rs by CCh, the Ca^2+^ stores released by CCh alone and by CCh with PTH are largely independent (Figure 4F). We suggested a similar conclusion previously, albeit with less decisive evidence, from results showing that depleting membranes of cholesterol selectively abolished the Ca^2+^ signals evoked by CCh without affecting those evoked by CCh with PTH (Tovey and Taylor, 2013).

### PTH-Evoked Ca^2+^ Release Requires Continuous Activation of M_3_Rs

Methylatropine is a competitive antagonist of M_3_Rs and, as expected, it abolished the Ca^2+^ signals evoked by CCh (data not shown). During sustained exposure to 30 μM CCh, the increase in [Ca^2+^]_i_ evoked by subsequent addition of PTH was abolished when methylatropine was added with the PTH (Figures S1A–S1C). Neither CCh nor methylatropine affected the amount of cAMP produced in response to PTH (Figure S1D). These results suggest three conclusions. They demonstrate that the response to PTH requires ongoing activation of M_3_Rs and is not a long-lasting consequence of their prior activation. They indicate that every step in the signaling pathway linking M_3_Rs to activation of IP_3_Rs is rapidly reversed when CCh can no longer reassociate with M_3_Rs. This second conclusion is consistent with rapid degradation of IP_3_ in cells (t_½_ ≤ 10 s) (Fink et al., 1999, Matsu-ura et al., 2006, Wang et al., 1995), and it suggests rapid termination of all preceding steps in the signaling pathway, including G protein de-activation and dissociation of IP_3_ from IP_3_Rs. Finally, the results demonstrate that there is no desensitization of M_3_Rs during sustained incubations with CCh.

In rat basophilic leukemia cells, store-operated Ca^2+^ entry (SOCE) is required for resynthesis of the pool of phosphatidylinositol 4,5-bisphosphate that sustains IP_3_ production during activation of leukotriene receptors (Alswied and Parekh, 2015). There appears to be no such requirement for SOCE in HEK-PR1 cells, because throughout a 60 min stimulation with a submaximal concentration of CCh in the absence of extracellular Ca^2+^, the formation of IP_3_ was sustained (Figures 4B and S1A–S1C).

### PTH Recruits More Sensitive IP_3_Rs

**Figure 5:**
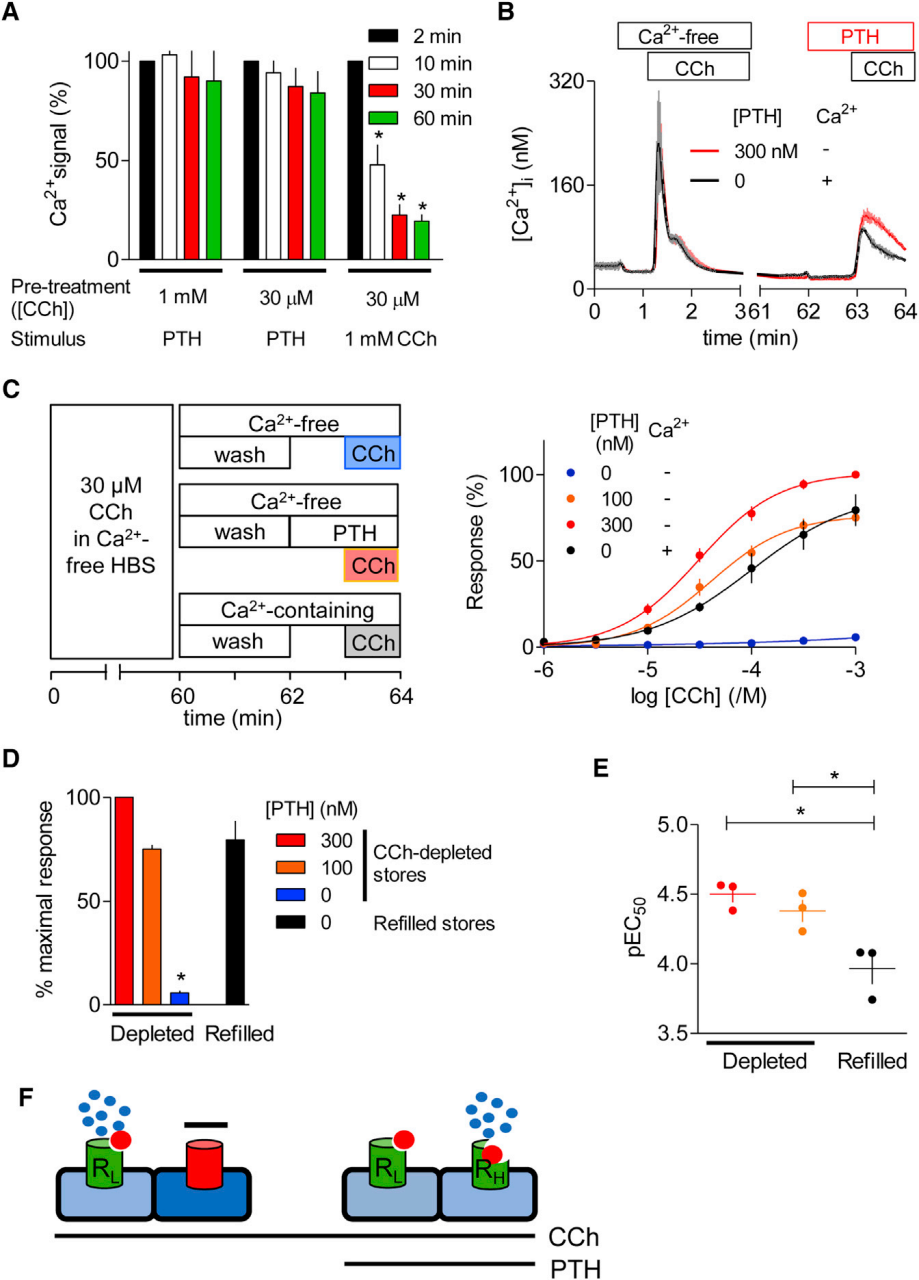
PTH Recruits More Sensitive IP_3_Rs (A) HEK-PR1 cells were pre-stimulated with 30 μM or 1 mM CCh for the indicated periods in Ca^2+^-free HBS before addition of PTH (100 nM) or CCh (1 mM), as indicated. Results show peak increases in [Ca^2+^]_i_ evoked by the final stimulus expressed as a percentage of that evoked when it was presented 2 min after the first addition of CCh. ^∗^p < 0.05, one-way ANOVA and Tukey’s post hoc test. (B) The CCh-sensitive Ca^2+^ stores were first emptied by incubating cells for 60 min with 30 μM CCh in Ca^2+^-free HBS to determine the CCh sensitivity of the stores that respond to CCh alone or CCh with PTH. Cells were then washed in Ca^2+^-free HBS to remove CCh (2 min) and then stimulated with PTH and CCh in Ca^2+^-free HBS (to determine the CCh sensitivity of the CCh/PTH-responsive stores). Alternatively, cells were washed in normal HBS (to allow intracellular stores to refill) and then stimulated with CCh (to determine the sensitivity of the CCh-responsive stores). Shown are typical traces for the indicated treatments (mean ± SD for three replicates). (C) Summary, with the protocol shown alongside, shows the concentration-dependent effects of CCh on the peak increase in [Ca^2+^]_i_ after the indicated treatments. Results are normalized to the maximal response evoked by CCh with 300 nM PTH. (D and E) Summary results show the effects of different concentrations of PTH on the maximal amplitude of the Ca^2+^ signal (D) and the sensitivity (pEC_50_) to CCh (E). (F) The results suggest that PTH unmasks IP_3_Rs with increased affinity for IP_3_ (from R_L_ to R_H_) in a discrete Ca^2+^ store. Results are mean ± SEM, n = 3 (A and C–E). ^∗^p < 0.05, one-way ANOVA and Tukey’s post hoc test (D and E).

After sustained stimulation of HEK-PR1 cells with a submaximal (30 μM) or maximal (1 mM) concentration of CCh to deplete the CCh-sensitive Ca^2+^ stores, the subsequent response to PTH was the same for both CCh concentrations (Figure 5A). These results extend those shown in Figure 4 by demonstrating that even sustained (60 min) stimulation with a maximally effective CCh concentration has no effect on the subsequent response to PTH. Furthermore, the results demonstrate that a low CCh concentration is as effective as a maximal CCh concentration in allowing PTH to evoke Ca^2+^ signals. This suggests that the IP_3_Rs recruited by PTH are more sensitive to IP_3_ than those responding to CCh alone.

The apparent independence of the Ca^2+^ stores released by CCh alone or CCh with PTH (Figure 4F) allowed us to directly determine the CCh sensitivity of the two stores using the protocol shown in Figure 5B. This involved depleting the CCh-sensitive stores by sustained stimulation in Ca^2+^-free HBS, washing the cells, and then determining their sensitivity to CCh with PTH. Under these conditions, there was no response to CCh or PTH alone, but CCh with PTH stimulated Ca^2+^ release (Figures 5B and 5C). To determine the sensitivity of the stores that respond to CCh alone, the stores were allowed to refill with Ca^2+^ by incubation in normal HBS during the washing period and subsequent stimulation with CCh. The comparison is valid because Ca^2+^ entry does not contribute to the peak Ca^2+^ signals evoked by CCh or CCh with PTH (see Figure S3B). The results demonstrate that PTH causes a concentration-dependent increase in the maximal response (Figure 5D), and that the stores responding to CCh with PTH are more sensitive to CCh than those responding to CCh alone (Figure 5E). We conclude that PTH causes a concentration-dependent unmasking of IP_3_Rs within a discrete Ca^2+^ store, and that these unmasked IP_3_Rs have enhanced sensitivity to CCh (Figure 5F). We showed previously, using small interfering RNA (siRNA), that in HEK-PR1 cells responses to CCh alone were most affected by loss of IP_3_R1, whereas responses to CCh with PTH were most affected by loss of IP_3_R2 (Tovey et al., 2008). Hence, our conclusion that PTH unmasks sensitive IP_3_Rs aligns with evidence that IP_3_R2, the most sensitive IP_3_R subtype (Iwai et al., 2005), is selectively regulated by PTH.

The functional independence of the Ca^2+^ stores released by CCh alone or CCh with PTH (Figure 4F) implies that IP_3_Rs in the stores responding to CCh alone are insensitive to cAMP. We speculated previously that association of these IP_3_Rs with M_3_R signaling pathways might allow local delivery of IP_3_ at concentrations more than sufficient for their maximal activation, thereby depriving the IP_3_Rs of any additional benefit from cAMP (Tovey and Taylor, 2013). However, this explanation now seems unlikely because we have found no evidence that CCh causes local saturation of IP_3_Rs with IP_3_ (Konieczny, 2015). Our new results, suggesting that PTH unmasks IP_3_Rs within a distinct Ca^2+^ store, provide a simple explanation for the lack of effect of PTH on the Ca^2+^ stores that respond to CCh alone, because their IP_3_Rs are already accessible to IP_3_.

The results so far prompt experiments designed to address the mechanism by which PTH (through cAMP) unmasks IP_3_Rs and the means by which two intracellular Ca^2+^ stores maintain their functional independence.

### IRBIT Is Unlikely to Mediate the Effect of PTH on Ca^2+^ Signals

The phosphoprotein, IRBIT (IP_3_R-binding protein released by IP_3_), is an endogenous IP_3_R antagonist (Ando et al., 2003, Devogelaere et al., 2006) that is expressed in HEK293 cells (Kiefer et al., 2009). Because a protein homologous to the C-terminal region of IRBIT, *S*-adenosylhomocysteine-hydrolase (AHCY), binds cAMP (Kloor et al., 2009), IRBIT is a candidate for suppressing IP_3_R activity. Furthermore, IRBIT has been implicated in synergistic regulation of fluid secretion by cAMP and IP_3_-evoked Ca^2+^ release, where phosphorylation of IP_3_Rs by PKA was proposed to facilitate Ca^2+^ release by reciprocally regulating the affinity of IP_3_R1 for IP_3_ and IRBIT (Park et al., 2013).

Two different siRNAs to IRBIT, which inhibited IRBIT expression by ∼90% without affecting expression of IP_3_R1 (Figure S2A), had no significant effect on either the concentration-dependent effects of CCh on [Ca^2+^]_i_ or the potentiating effect of any PTH concentration (Figures S2B and S2C). We also used baculovirus to achieve high levels of expression of IRBIT or a dominant-negative form (IRBIT-S68A) (Ando et al., 2006) in HEK-PR1 cells (Figure S2D). Expression of these proteins had no effect on the Ca^2+^ signals evoked by CCh alone or CCh with PTH (Figures S2E and S2F). We conclude that IRBIT does not contribute to the effects of PTH on CCh-evoked Ca^2+^ signals.

It is surprising, when endogenous IRBIT has been reported to inhibit IP_3_-evoked Ca^2+^ signals in other cells (Ando et al., 2006, Devogelaere et al., 2006, Zaika et al., 2011), that neither overexpression of IRBIT nor its inhibition should affect IP_3_-evoked Ca^2+^ signals in HEK-PR1 cells (Figure S2). Because IRBIT must be phosphorylated before it can bind to IP_3_Rs (Ando et al., 2006, Devogelaere et al., 2007, Kiefer et al., 2009), we suggest that the mechanisms responsible for phosphorylation of IRBIT may be inactive in HEK-PR1 cells. Whatever the explanation for the lack of effect of IRBIT on IP_3_-evoked Ca^2+^ release, it seems clear that dissociation of IRBIT from IP_3_Rs is not the means by which cAMP unmasks IP_3_R activity.

### Stores Depleted by CCh or CCh with PTH Are Similarly Effective in Evoking SOCE

SOCE is triggered by loss of Ca^2+^ from the ER, leading to association of stromal interaction molecule 1 (STIM1) and Orai at ER-plasma membrane junctions (Lewis, 2011). Previous work established that, in HEK-PR1 cells, CCh-evoked Ca^2+^ entry is entirely mediated by SOCE (López Sanjurjo et al., 2014). We considered whether the Ca^2+^ stores emptied by CCh or CCh with PTH might differ in their abilities to evoke SOCE. The peak increases in [Ca^2+^]_i_ evoked by CCh alone or CCh with PTH were, as expected, entirely mediated by Ca^2+^ release from intracellular stores (Figures S3A and S3B). Comparison of the initial peak increases in [Ca^2+^]_i_ evoked by CCh or CCh with PTH (Ca^2+^ release) with the amplitude of the subsequent sustained increase in [Ca^2+^]_i_ (SOCE) revealed that the relationship between the two Ca^2+^ signals was indistinguishable for cells stimulated with the different stimuli (Figure S3C). These results, which are also consistent with previous reports that intracellular stores must be substantially depleted of Ca^2+^ before they effectively evoke STIM1 translocation (Suzuki et al., 2014) and activation of SOCE (Bird et al., 2009, Luik et al., 2008), suggest that stores depleted by CCh alone or CCh with PTH are equally capable of stimulating SOCE. We also considered whether translocation of STIM1 after store depletion might reveal the subcellular location of the Ca^2+^ stores emptied by CCh alone or with PTH. In HEK-PR1 cells expressing mCherry-STIM1, the stimuli evoked formation of STIM1 puncta near the plasma membrane, but there was no discernible difference in the spatial distribution of the puncta formed after stimulation with CCh alone or CCh with PTH (Figure S3D).

### The Golgi Apparatus Is Not the Independent Ca^2+^ Store Recruited by PTH

The ER and Golgi apparatus accumulate Ca^2+^, IP_3_ can evoke Ca^2+^ release from both organelles (Pizzo et al., 2011, Rodríguez-Prados et al., 2015, Wong et al., 2013), and recent work suggests that in cardiac myocytes spontaneous Ca^2+^ release through ryanodine receptors in the Golgi apparatus is enhanced by activation of G_s_-coupled receptors (Yang et al., 2015). Ca^2+^ accumulation by the Golgi apparatus is mediated by a SERCA and, within the *trans*-Golgi, by a secretory pathway Ca^2+^-ATPase (SPCA) (Aulestia et al., 2015). Both Ca^2+^ pumps are inhibited by thapsigargin, although SPCAs are less sensitive to thapsigargin than SERCAs (Dode et al., 2006). Because considerable evidence suggests that the ER is luminally continuous (Park et al., 2000), allowing free movement of proteins as large as GFP (Dayel et al., 1999), we considered whether the Golgi apparatus might provide the independent Ca^2+^ store recruited by PTH. The latter would be consistent with evidence that the Ca^2+^ release evoked by CCh or CCh with PTH is abolished by pretreatment with thapsigargin (Short and Taylor, 2000).

**Figure 6:**
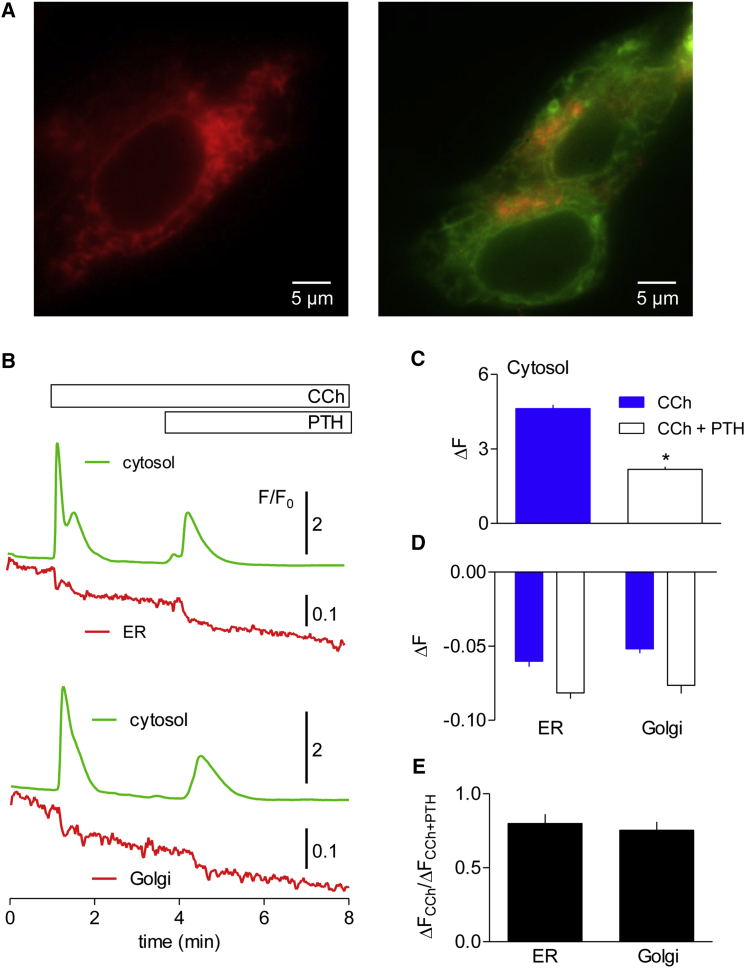
The Golgi Apparatus Is Not the Independent Ca^2+^ Store Recruited by PTH (A) Typical widefield images of HEK-PR1 cells expressing ER-LAR-GECO1 (left) and Golgi-LAR-GECO1 with GFP-ER (right). (B) HEK-PR1 cells expressing Ca^2+^ sensors within either the ER or Golgi lumen and loaded with a cytosolic Ca^2+^ indicator (fluo-8) were stimulated as indicated, in Ca^2+^-free HBS with CCh (1 mM) and PTH (300 nM). Typical traces, each from a single cell, show the simultaneous recordings of cytosolic and luminal fluorescence measured in several regions of interest in each cell (as F/F_0_, where F_0_ is the average fluorescence recorded for 15 s before any stimulation). (C and D) Summary results show fluorescence changes for the cytosolic (C) and luminal (D) indicators (as ΔF = F_peak_ − F_pre_, where F_peak_ is the peak F/F_0_ value, and F_pre_ is the value determined immediately before stimulation). The code applies to both panels. (E) For each cell, the ratio of the fluorescence signals (ΔF) evoked by CCh and PTH is shown for the ER and Golgi sensors. (C–E) Results are mean ± SEM from at least 27 cells. ^∗^p < 0.05, Student’s t test, comparing CCh with CCh and PTH (C) or ER relative to Golgi (D and E).

We used a low-affinity, red Ca^2+^ sensor (LAR-GECO1, K_D_ = 24 μM) (Wu et al., 2014) targeted to the lumen of either the ER or the medial/*trans*-Golgi apparatus (Figure 6A) to measure the free [Ca^2+^] within these organelles. These sensors were used with fluo-8 to report the changes in luminal and cytosolic [Ca^2+^] evoked by CCh and then PTH (Figure 6B). CCh and the subsequent addition of PTH evoked increases in [Ca^2+^]_i_ (Figure 6C), and they both caused decreases in the fluorescence of the ER and Golgi sensors (Figure 6D). Comparison of the effects of CCh and the subsequent addition of PTH on the ER and Golgi sensors (ΔF_CCh_/ΔF_CCh then PTH_) shows that neither organelle responded selectively to PTH (Figure 6E). The results suggest that the independence of the stores from which CCh or CCh with PTH release Ca^2+^ is not due to selective release of Ca^2+^ from the medial/*trans*-Golgi apparatus. We have not examined the *cis*-Golgi, which has been reported to have a higher luminal Ca^2+^ concentration and more IP_3_Rs than the medial/*trans*-Golgi (Pizzo et al., 2011).

### CCh and CCh with PTH Release Ca^2+^ from Intracellular Stores that Dynamically Exchange Ca^2+^ with the Cytosol

**Figure 7:**
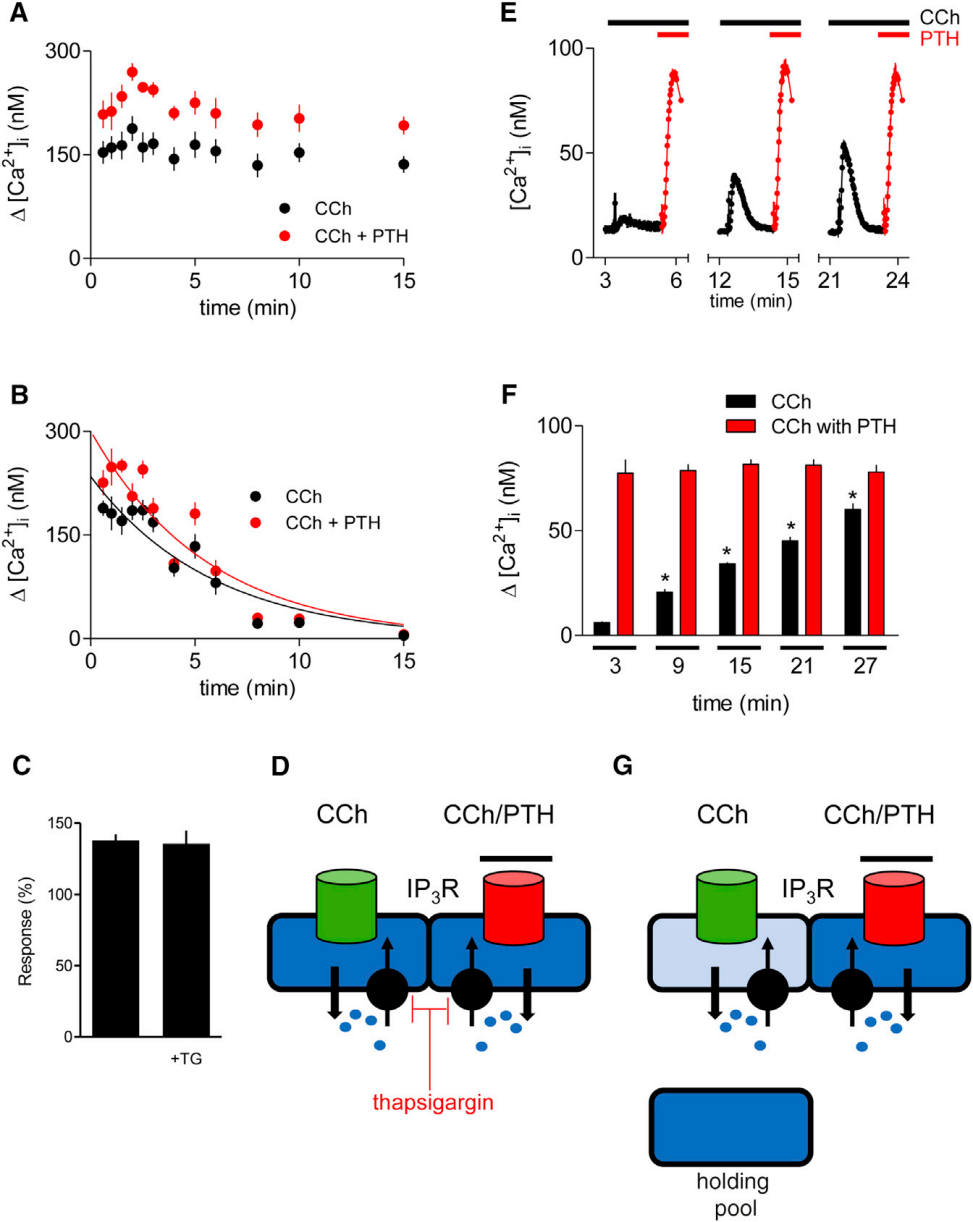
Independent Refilling of Dynamic Ca^2+^ Pools that Respond to CCh or CCh with PTH (A) HEK-PR1 cells were incubated in Ca^2+^-free HBS for the indicated times before addition of CCh (1 mM) alone or with PTH (100 nM). Results show peak increases in [Ca^2+^]_i_ (Δ[Ca^2+^]_i_). (B) Similar experiments in the presence of thapsigargin (1 μM) to inhibit SERCA. The lines are mono-exponential curve fits. Results show mean ± SEM, n = 3. (C) The increase in [Ca^2+^]_i_ evoked by CCh with PTH in Ca^2+^-free HBS alone or with thapsigargin (TG, 1 μM, 1 min) is shown relative to the time-matched response evoked by CCh alone (%, mean ± SEM, n = 3). Data are from (A) and (B). (D) Two distinct Ca^2+^ stores, responding to CCh alone or CCh with PTH, maintain their integrity despite rapidly recycling their Ca^2+^ through the cytosol. (E) HEK-PR1 cells were incubated with 30 μM CCh for 60 min in Ca^2+^-free HBS to deplete the CCh-sensitive Ca^2+^ stores and then washed twice with Ca^2+^-free HBS to remove CCh. Typical traces (mean ± SD from three wells) show the response to CCh (1 mM) and then PTH (100 nM) after the indicated periods of recovery in Ca^2+^-free HBS. (F) Summary results (mean ± SEM from three independent experiments) show the response to CCh alone and the subsequent response to PTH after the indicated periods of recovery. ^∗^p < 0.05, one-way ANOVA and Tukey’s post hoc test, relative to measurement at 3 min. (G) The stores that respond to CCh alone can refill in the absence of extracellular Ca^2+^ without affecting the Ca^2+^ content of the stores that respond to CCh with PTH. The “holding pool” from which these stores acquire Ca^2+^ has not been identified, although it may reside within mitochondria or lysosomes. See also Figure S4.

We next considered whether the independence of the Ca^2+^ stores released by CCh alone or CCh with PTH might reflect the existence of a store that only very slowly exchanges Ca^2+^ with the cytosol. In Ca^2+^-free HBS, the intracellular stores of unstimulated HEK-PR1 cells lose Ca^2+^ extremely slowly (Figure 3C), suggesting either that the stores exchange Ca^2+^ very slowly with the cytosol or that cells efficiently retain Ca^2+^ to allow rapid recycling to intracellular stores. Inhibition of SERCA with thapsigargin reveals that there is rapid cycling of Ca^2+^ across ER membranes. In Ca^2+^-free HBS without thapsigargin, there was no significant loss of the response to CCh or CCh with PTH after 15 min (Figure 7A), whereas with thapsigargin there was no response to either stimulus after 15 min (Figure 7B). The rate of decline of the response was indistinguishable for Ca^2+^ signals evoked by CCh alone (t_½_ = 4.0 ± 0.2 min, n = 3) or CCh with PTH (t_½_ = 3.9 ± 0.2 min) (Figure 7B), suggesting that the Ca^2+^ stores released by CCh or CCh with PTH have similar basal rates of Ca^2+^ leak. Because PTH evokes Ca^2+^ release from a thapsigargin-sensitive Ca^2+^ store (Short and Taylor, 2000), these results confirm that responses to PTH are not dependent on Ca^2+^ being chased from one pool to another (Figure 7C), and they demonstrate that segregation of the two intracellular stores is maintained despite rapid cycling of Ca^2+^ between the cytosol and stores (Figure 7D).

### The Two Ca^2+^ Stores Refill Independently

HEK-PR1 cells in Ca^2+^-free HBS were incubated for 60 min with 30 μM CCh to empty the CCh-sensitive Ca^2+^ stores; the cells were then rapidly washed in Ca^2+^-free HBS to remove CCh, and the responses to CCh alone or CCh with PTH in Ca^2+^-free HBS were assessed during this recovery period. At the end of the 60 min incubation with 30 μM CCh, the response to addition of 1 mM CCh had declined to 1% ± 1% (n = 3) of the initial response, consistent with previous results (Figures 3 and 4). Because there is no desensitization of M_3_Rs with this stimulus regime (Figures 4 and S1), the results confirm that the CCh-sensitive stores were empty at the end of the sustained incubation. During the subsequent recovery period in Ca^2+^-free HBS, the response to 1 mM CCh recovered relatively slowly (to ∼10 times the initial response after 27 min). However, the response to addition of PTH after CCh remained constant over the entire recovery period (Figures 7E and 7F). Hence, under conditions where the CCh-sensitive store substantially refilled, there was no effect on the Ca^2+^ content of the store released by CCh with PTH. We have not determined the source of the intracellular Ca^2+^ that replenished the CCh-sensitive store, although mitochondria (Rizzuto et al., 2012) or lysosomes (López Sanjurjo et al., 2014) are likely candidates. Others have also reported refilling of IP_3_-sensitive Ca^2+^ stores within the ER from unidentified intracellular sources (Suzuki et al., 2014). Our results, where CCh-sensitive stores refill without affecting the response to PTH, mirror those in Figure 4, where depletion of the CCh-sensitive stores had no impact on the subsequent response to PTH. Both sets of results establish the functional independence of the Ca^2+^ stores released by CCh alone and by CCh with PTH (Figure 7G).

### Conclusions

Substantial evidence suggests that the ER is luminally continuous, and so unlikely to provide a barrier to free movement of Ca^2+^ within the ER lumen (Dayel et al., 1999, Mogami et al., 1997, Park et al., 2000, Rizzuto and Pozzan, 2006), but other evidence suggests some functional compartmentalization of ER Ca^2+^ stores. In HEK293 cells, for example, CCh and ATP, via their respective PLC-coupled receptors, can release Ca^2+^ from different IP_3_-sensitive Ca^2+^ stores (Short et al., 2000). Further evidence for compartmentalization within ER Ca^2+^ stores includes measurements of sustained focal changes of luminal Ca^2+^ concentration within the ER and different responses of adjacent compartments to activation of IP_3_R and ryanodine receptors (Golovina and Blaustein, 1997). ER Ca^2+^ pools that differ in their susceptibilities to SERCA inhibitors further suggest a degree of compartmentalization (Aulestia et al., 2011). A recent cryo-electron tomographic analysis of ER-plasma membrane contact sites, where the lumen of some ER is very constricted, suggests a possible structural basis for compartmentalization of ER Ca^2+^ stores (Fernández-Busnadiego et al., 2015).

Our present results demonstrate a remarkable functional independence of two discrete ER Ca^2+^ stores that persists despite each rapidly exchanging Ca^2+^ with the cytosol. The first store expresses IP_3_Rs with modest affinity and responds to the IP_3_ produced in response to CCh alone. The second store expresses IP_3_Rs with greater affinity for IP_3_ (possibly IP_3_R2), but these IP_3_Rs are unmasked only in the presence of cAMP. We have not established the identities of the independent Ca^2+^ stores, although it is clear that IP_3_R2, which we showed to be important for responses to PTH (Tovey et al., 2008), has a different subcellular distribution to that of IP_3_R1 and IP_3_R3 (Figure S4). The interactions between PTH and CCh in HEK-PR1 cells are reminiscent of those between PTH and ATP in osteoblasts (Buckley et al., 2001), suggesting that the mechanisms we have described here may be widespread. We conclude that a strict functional compartmentalization of ER Ca^2+^ stores allows IP_3_ alone and IP_3_ with cAMP to release Ca^2+^ from discrete stores. Our results suggest a hitherto unexpected versatility in IP_3_-evoked Ca^2+^ release from the ER.

## Experimental Procedures

### Measurements of [Ca^2+^]_i_ and Intracellular cAMP

HEK-PR1 cells (Short and Taylor, 2000) were cultured as described previously (Tovey et al., 2008). HEK293 cells (without PTH receptors) were used for some experiments because ATP evoked larger Ca^2+^ signals in these cells than in HEK-PR1 cells. Measurements of intracellular free Ca^2+^ concentration ([Ca^2+^]_i_) in single cells and populations of fluo-4-loaded HEK-PR1 cells were performed as previously described (Tovey et al., 2008). Intracellular cAMP was measured as previously described (Pantazaka et al., 2013) (Supplemental Experimental Procedures).

### Measurements of Luminal-Free [Ca^2+^] within the ER and Golgi Apparatus

A low-affinity (K_D_ = 24 μM), red genetically encoded Ca^2+^ sensor (LAR-GECO1) was used to record the luminal [Ca^2+^] within the ER ([Ca^2+^]_ER_ using ER-LAR-GECO1) (Wu et al., 2014) or within the Golgi apparatus ([Ca^2+^]_GA_ using Golgi-LAR-GECO1). Details are given in the Supplemental Experimental Procedures.

### Expression of IRBIT and siRNA-Mediated Knockdown

BacMam viruses were used to express IRBIT and IRBIT-S68A in HEK-PR1 cells. Cells were transfected with siRNAs to reduce IRBIT expression in HEK-PR1 cells (Supplemental Experimental Procedures).

### Statistical Analyses

The experiments reported were completed over a prolonged period during which there was some variation between absolute values for changes in [Ca^2+^]_i_ and sensitivities to CCh and PTH. Hence, all statistical comparisons use observations from matched analyses. For each experiment, the concentration-effect relationship was fitted to a logistic equation (GraphPad Prism version 5). From each experiment, pEC_50_ (−log of the half-maximally effective concentration [EC_50_] in M) and the maximal response were obtained and used for statistical analyses. Most graphs show mean results from several experiments, but values (pEC_50_, etc.) were computed from individual experiments before pooling for statistical comparisons.

## Author Contributions

V.K. performed experiments. S.C.T. contributed to fluorescence experiments. S.M. performed western blot (WB) and analyses of STIM1. D.L.P. contributed to design and analysis of targeted Ca^2+^ indicators. C.W.T. supervised the project and contributed to data analysis. C.W.T with V.K and D.L.P. wrote the paper. All authors contributed to review of the paper.

## Supplemental Information

Document S1. Supplemental Experimental Procedures and Figures S1–S4

Document S2. Article plus Supplemental Information
