# Supplementary material for: Cyclic AMP Recruits a Discrete Intracellular Ca2+ Store by Unmasking Hypersensitive IP3 Receptors
Source: Cell Rep. 2017 Jan 17;18(3):711–22. doi: 10.1016/j.celrep.2016.12.058 (PMC5276804; doi:10.1016/j.celrep.2016.12.058)
Supplement: Document S1. Supplemental Experimental Procedures and Figures S1–S4 [file mmc1.pdf]

Cell Reports, Volume *18*

Supplemental Information

Cyclic AMP Recruits a Discrete Intracellular Ca^{2+} Store by Unmasking Hypersensitive IP_3 Receptors

Vera Konieczny, Stephen C. Tovey, Stefania Mataragka, David L. Prole, and Colin W. Taylor

Abbreviations

BAPTA, 1,2-bis(*O*-aminophenoxy)ethane-N,N,N',N'-tetraacetic acid; $[Ca^{2+}]_i$ intracellular free Ca^{2+} concentration; $[Ca^{2+}]_{ER}$, $[Ca^{2+}]_{GA}$, free Ca^{2+} concentration within the ER, Golgi apparatus; cAMP, 3′,5′-cyclic adenosine monophosphate; CCh, carbachol (carbamylcholine); 8-Br-cAMP, 8-bromo cAMP; EC_{50}, half-maximally effective concentration; EGFP, enhanced green fluorescent protein; HBS, HEPES-buffered saline; HEK-PR1, human embryonic kidney cell stably expressing human type 1 PTH receptor; IP_3 inositol 1,4,5-trisphosphate; IP_3R, IP_3 receptor; IRBIT, IP_3R-binding protein released by IP_3; Mfm, 5-methylfurmethiodide; MOI, multiplicity of infection; M_r, relative molecular mass; pEC_{50}, -log EC_{50}; PCR, polymerase chain reaction; PFU, plaque-forming unit; PKA, cyclic AMP-dependent protein kinase; PTH, parathyroid hormone (residues 1-34); SD, standard deviation; SEM, standard error of the mean; SERCA, sarcoplasmic/endoplasmic reticulum Ca^{2+}-ATPase; siRNA, small interfering RNA; SOCE, store-operated Ca^{2+} entry; SPCA, secretory pathway Ca^{2+}-ATPase; $t_{½}$, half-time; STIM1, stromal interaction molecule 1; TIRFM, total internal refection fluorescence microscopy; WB, Western blot.

SUPPLEMENTAL EXPERIMENTAL PROCEDURES

Materials

Cell culture materials and fluo-4 acetoxymethyl ester (fluo-4/AM) were from Life Technologies (Paisley, UK). Fluo-8/AM was from AAT Bioquest (Sunnyvale, CA, USA). Poly-L-lysine, Triton X-100, carbamylcholine hydrochloride (carbachol, CCh), methylatropine (Atr), isoprenaline, Pluronic F-127, trichloroacetic acid, alumina, Dowex 50WX4-400 and dimethyl sulfoxide (DMSO) were from Sigma-Aldrich (Poole, Dorset, UK). Human parathyroid hormone (residues 1-34, PTH) was from Bachem (St Helens, UK). Thapsigargin was from Tocris (Bristol, UK). 1,2-bis(*O*-aminophenoxy)ethane-N,N,N',N'-tetraacetic acid (BAPTA) was from Molekula (Dorset, UK). Ionomycin was from Apollo Scientific (Bredbury, UK). Imidazole was from Thermo Scientific (Waltham, MA, USA). ^{3}H-adenine (18.4 Ci/mmol) and Ultima Gold scintillant were from Perkin Elmer (Waltham, MA, USA).

Measurements of $[Ca^{2+}]_i$

HEK293 cells stably expressing human type 1 PTH receptor (HEK-PR1 cells) (Short and Taylor, 2000) were cultured as described previously (Tovey et al., 2008). HEK293 cells (without PTH receptors) were used for some experiments because ATP evoked larger Ca^{2+} signals in these cells than in HEK-PR1 cells.

For measurements of intracellular free Ca^{2+} concentration ($[Ca^{2+}]_i$) in cell populations, HEK-PR1 cells (8 x 10^4 cells/well in poly-L-lysine-coated black full-area 96-well plates, Greiner Bio-One) were grown to confluence (48 h). The cells were washed in HEPES-buffered saline (HBS) and loaded with fluo-4 by incubation with fluo-4/AM (2 µM) in HBS with Pluronic F-127 (0.02 %, v/v) for 1 h at 20 °C in the dark. Cells were then washed and incubated with HBS for 45 min to allow de-esterification of the fluo-4/AM. HBS had the following composition (in mM): NaCl (135), KCl (5.9), $MgCl_2$ (1.2), $CaCl_2$ (1.5), HEPES (11.6), D-glucose (11.5), pH 7.3. Fluorescence (excitation, 485 nm; emission, 525 nm) was recorded at 1.44-s intervals in HBS (100-160 µl) at 20 °C using a FlexStation III fluorescence plate-reader (MDS Analytical Devices, Wokingham, UK) (Tovey et al., 2008). Minimum (F_{min}) and maximum (F_{max}) fluorescence values were determined from parallel wells on each plate by addition of 0.1 % Triton X-100 with 10 mM BAPTA (F_{min}) or 10 mM $CaCl_2$ (F_{max}). Fluorescence values (F) were calibrated to $[Ca^{2+}]_i$ from:

$$[Ca^{2+}]_i = K_D \times \frac{F - F_{min}}{F_{max} - F}$$

The K_D of fluo-4 was assumed to be 345 nM (Gee et al., 2000). Fluo-8 was used for some experiments (K_D = 389 nM).

For experiments in Ca^{2+}-free HBS, cells were incubated in normal HBS (60 µl) to maintain the Ca^{2+} content of the intracellular stores before addition of Ca^{2+}-free HBS containing 5 mM BAPTA (60 µl) 20 s before stimulation (final BAPTA and Ca^{2+} concentrations of 2.5 mM and 0.75 mM, respectively; free $[Ca^{2+}]$ < 60 nM). Stimuli (20 µl) were added by an automated pipetting system from stock solutions prepared in HBS or Ca^{2+}-free HBS, as appropriate.

For single-cell measurements of $[Ca^{2+}]_i$, cells were seeded onto poly-L-lysine-coated 35-mm glass-bottomed dishes (MatTek, number 1 coverglass) and grown to ~80 % confluence (48 h). Cells were loaded with fluo-4 and fluorescence was recorded at 20°C using an Olympus IX81 inverted microscope with a 40x/1.35 NA objective. Cells were illuminated every 1 s with a mercury xenon lamp and U-MNIBA filter set (Olympus, excitation 470-495 nm, emission 510-550 nm). Fluorescence was detected using an Andor iXon 897 EMCCD camera. Fluorescence values were calibrated to $[Ca^{2+}]_i$ as described above. Images were processed using CellR software (Olympus).

Measurements of Intracellular cAMP

HEK-PR1 cells (4.5 x 10^5 cells/well) grown to confluence in 24-well plates (48 h) were incubated for 2 h in normal growth medium with ^{3}H-adenine (1 µCi/well) at 37 °C with 5 %

CO_2. The cells were washed twice with HBS and then incubated in Ca^{2+}-free HBS with appropriate stimuli at 20 °C. After 5 min, the medium was removed, and the reaction was terminated by addition of ice-cold trichloroacetic acid (5 %). ^{3}H-cAMP was separated from other ^{3}H-adenine nucleotides by sequential column chromatography using a Dowex cation exchange resin and alumina as described (Pantazaka et al., 2013). The activity of the eluates was determined by liquid scintillation counting in Ultima Gold scintillant. ^{3}H-cAMP levels are expressed as percentages of the sum of ^{3}H-ATP, ^{3}H-ADP and ^{3}H-cAMP activities.

Measurements of Luminal Free [Ca^{2+}] Within the ER and Golgi Apparatus

A low-affinity (K_D = 24 μM) red genetically encoded Ca^{2+} sensor (LAR-GECO1) was used to record the luminal [Ca^{2+}] within the ER ($[Ca^{2+}]_{ER}$ using ER-LAR-GECO1) (Wu et al., 2014) or within the Golgi apparatus ($[Ca^{2+}]_{GA}$ using Golgi-LAR-GECO1). Targeting to the medial/trans Golgi apparatus (Llopis et al., 1998) was achieved by fusing the N-terminus of LAR-GECO1 to a 61-residue N-terminal sequence from human β-1,4-galactosidase 1 (MRLREPLLSGSAAMPGASLQRACRLLVAVCALHLGVTLVYYLAGRDLSRLPQLVG VSTPLQ) (Tian et al., 2014). The targeting signal and flanking restriction sites (BamHI and EcoRI) were added to LAR-GECO1 by PCR using CMV-ER-LAR-GECO1 (Addgene plasmid 61244) (Wu et al., 2014) as a template. Digestion with BamHI and EcoRI then allowed subcloning into similarly digested pcDNA3.1(+) (Thermo Fisher) to give Golgi-LAR-GECO1.

HEK-PR1 cells were seeded on fibronectin-coated 35-mm glass-bottomed dishes (MatTek, number 1 coverglass) and transfected (1 μg DNA/dish) after 24 h using TransIT-LT1 (Mirus Bio, Madison, WI, USA). Cells were used 24 h after transfection. For simultaneous measurements of $[Ca^{2+}]_i$ and either $[Ca^{2+}]_{ER}$ or $[Ca^{2+}]_{GA}$, cells were loaded with fluo-8/AM as described for fluo-4/AM, and imaged using an Olympus IX83 inverted microscope with a 100x/1.49 NA objective. Cells were alternately illuminated (50-100 ms for each wavelength on a 400-ms cycle) with a 470-nm light-emitting diode (LED) (Spectra X, Lumencor) for visualizing fluo-8 fluorescence and a 561-laser (Coherent) for visualizing ER-LAR-GECO1 and Golgi-LAR-GECO1, via a 405/488/561/647-nm quad band dichroic/emitter (TRF89902, Chroma Technology). Fluorescence was detected using an Andor 6 iXon Ultra EMCCD camera. Images were processed using MetaMorph (Molecular Devices).

Expression of IRBIT and siRNA-Mediated Knockdown

The coding sequences of mouse IRBIT (sequence identifier: GI 17390492) and IRBIT with an N-terminal EGFP tag and linker sequence (SGRTQISSSSFEF were cloned into pENTR1A (Life Technologies) as SalI/NotI fragments. For the dominant-negative form of IRBIT (IRBIT-S68A) (Ando et al., 2006), the codon TCA encoding Ser68 was mutated to GCA (Ala) using the QuikChange Lightning Mutagenesis Kit (Agilent) following the manufacturer's instructions.

To generate BacMam viruses (Fornwald et al., 2007) for expression of IRBIT and IRBIT-S68A, the open reading frames of the IRBIT constructs were transferred from pENTR1A to the pCMV-DEST vector (Life Technologies). Viral stocks (P1-P3) of each construct were prepared following the manufacturer's instructions (Life Technologies). For transduction of HEK-PR1 cells, the P3 viral stock (10 µl, ~10^8 PFU/ml) was added to HEK-PR1 cells in suspension (10^5 cells in 200 µl of culture medium, MOI ~10 PFU/cell). Cells were used after 48 h. Western blots (WB) using a mouse polyclonal anti-IRBIT antiserum (1:750, #H00010768-A01, Abnova, Littleton, CO, USA) were used to quantify IRBIT expression.

For siRNA-mediated knockdown of IRBIT, HiPerFect transfection reagent (Qiagen, 0.75 µl/well or 12 µl/well for 96- and 6-well plates, respectively) was added to 40 nM siRNA in serum-free DMEM/F-12 GlutaMAX medium (50 µl or 200 µl for 96- and 6-well plates). The mixture was then incubated for 15 min at 22 °C to allow complex formation. Cells were seeded onto poly-L-lysine-coated wells in normal medium: 3 x 10^4 cells/well for 96-well plates used for measurements of $[Ca^{2+}]_i$, and 4.8 x 10^5 cells/well in 6-well plates for WB analysis. The siRNAs used were Hs_AHCYL1_1 and Hs_AHCYL1_2 (SI00090328, SI00090335) and the AllStars Negative Control siRNA (SI036550318) (Qiagen).

Translocation of mCherry-STIM1 to ER-Plasma Membrane Junctions
HEK-PR1 cells grown on poly-L-lysine-coated 35-mm glass-bottomed dishes (MatTek) were transfected with STIM1-mCherry (Wang et al., 2010) using TransIT-LT1 (1 µg plasmid DNA/dish). After 24 h, cells were loaded with the Ca^{2+} indicator, Cal-520, by incubation with Cal-520/AM (2 µM, Stratech Scientific Ltd) in HBS containing F-127 pluronic acid (0.02%). After 1 h, the cells were washed and incubated in HBS to allow de-esterification of the indicator (30 min). TIRFM, at 20°C in HBS, used an Olympus IX83 inverted microscope with 100x/1.49 NA TIRF objective. Diode-pumped solid-state lasers (iLas Laser System, Cairn) were used to excite the fluorescence of Cal-520 (488 nm, emission 525 nm) and m-Cherry (561 nm, emission 630 nm). Emitted light was captured by an Andor iXon 897 EMCCD camera at 1-s intervals for Cal-520 (50-ms capture interval) and (to minimize bleaching) for 20 ms from frames captured before or 2 min after addition of each stimulus for m-Cherry. Images were corrected for background by subtraction of fluorescence determined from an area outside the cell.

SUPPLEMENTAL REFERENCES

Ando, H., Mizutani, A., Kiefer, H., Tsuzurugi, D., Michikawa, T., and Mikoshiba, K. (2006). IRBIT suppresses IP_3 receptor activity by competing with IP_3 for the common binding site on the IP_3 receptor. Mol Cell *22*, 795-806.

Fornwald, J.A., Lu, Q., Wang, D., and Ames, R.S. (2007). Gene expression in mammalian cells using BacMam, a modified baculovirus system. Methods Mol Biol *388*, 95-114.

Gee, K.R., Brown, K.A., Chen, W.N., Bishop-Stewart, J., Gray, D., and Johnson, I. (2000). Chemical and physiological characterization of fluo-4 Ca^{2+}-indicator dyes. Cell Calcium *27*, 97-106.

Llopis, J., McCaffery, J.M., Miyawaki, A., Farquhar, M.G., and Tsien, R.Y. (1998). Measurement of cytosolic, mitochondrial, and Golgi pH in single living cells with green fluorescent proteins. Proc Natl Acad Sci USA *95*, 6803-6808.

Pantazaka, E., Taylor, E.J.A., Bernard, W., and Taylor, C.W. (2013). Ca^{2+} signals evoked by histamine H_1 receptors are attenuated by activation of prostaglandin EP_2 receptors in human aortic smooth muscle. Br J Pharmacol *169*, 1624-1634.

Short, A.D., and Taylor, C.W. (2000). Parathyroid hormone controls the size of the intracellular Ca^{2+} stores available to receptors linked to inositol trisphosphate formation. J Biol Chem *275*, 1807-1813.

Tian, G., Ropelewski, P., Nemet, I., Lee, R., Lodowski, K.H., and Imanishi, Y. (2014). An unconventional secretory pathway mediates the cilia targeting of peripherin/rds. J Neurosci *34*, 992-1006.

Tovey, S.C., Dedos, S.G., Taylor, E.J.A., Church, J.E., and Taylor, C.W. (2008). Selective coupling of type 6 adenylyl cyclase with type 2 IP_3 receptors mediates a direct sensitization of IP_3 receptors by cAMP. J Cell Biol *183*, 297-311.

Wang, Y., Deng, X., Mancarella, S., Hendron, E., Eguchi, S., Soboloff, J., Tang, X.D., and Gill, D.L. (2010). The calcium store sensor, STIM1, reciprocally controls Orai and $Ca_V1.2$ channels. Science *330*, 105-109.

Wu, J., Prole, D.L., Shen, Y., Lin, Z., Gnanasekaran, A., Liu, Y., Chen, L., Zhou, H., Chen, S.R., Usachev, Y.M., *et al.* (2014). Red fluorescent genetically encoded Ca^{2+} indicators for use in mitochondria and endoplasmic reticulum. Biochem J *464*, 13-22.

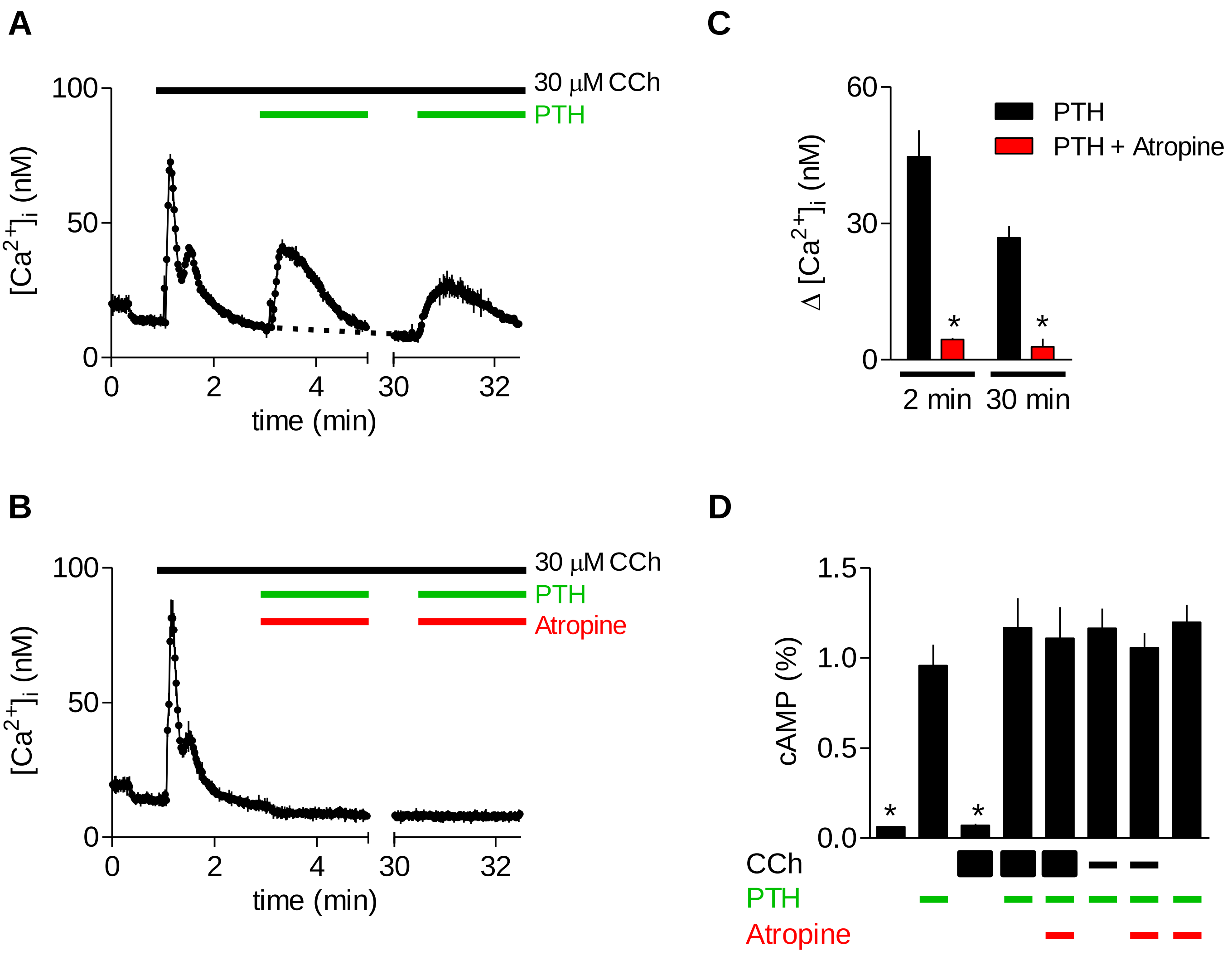

Figure S1. PTH-Evoked Ca^{2+} Release Requires Continuous Stimulation of M_3Rs. Related to Figure 4.

(A, B) HEK-PR1 cells were stimulated with 30 μM CCh in Ca^{2+}-free HBS before addition of PTH alone (100 nM) (A) or with methylatropine (10 μM) (B). Each panel shows results from 2 separate experiments in which PTH was added 2 or 30 min after addition of CCh. The typical traces show mean ± SD from 3 measurements.
(C) Summary shows the increase in $[Ca^{2+}]_i$ evoked by PTH (mean ± SEM from 3 independent experiments). $^{*}P < 0.05$, Student's *t*-test.
(D) Intracellular cAMP concentrations (% of ^{3}H-adenine nucleotides) in HEK-PR1 cells measured 5 min after addition of PTH (100 nM) with methylatropine (10 μM) and/or CCh (1 mM or 30 μM, thick and thin bar, respectively) as indicated. Results show means ± SEM, n = 3. $^{*}P < 0.05$, one-way ANOVA and Tukey's post hoc test, relative to all other conditions.

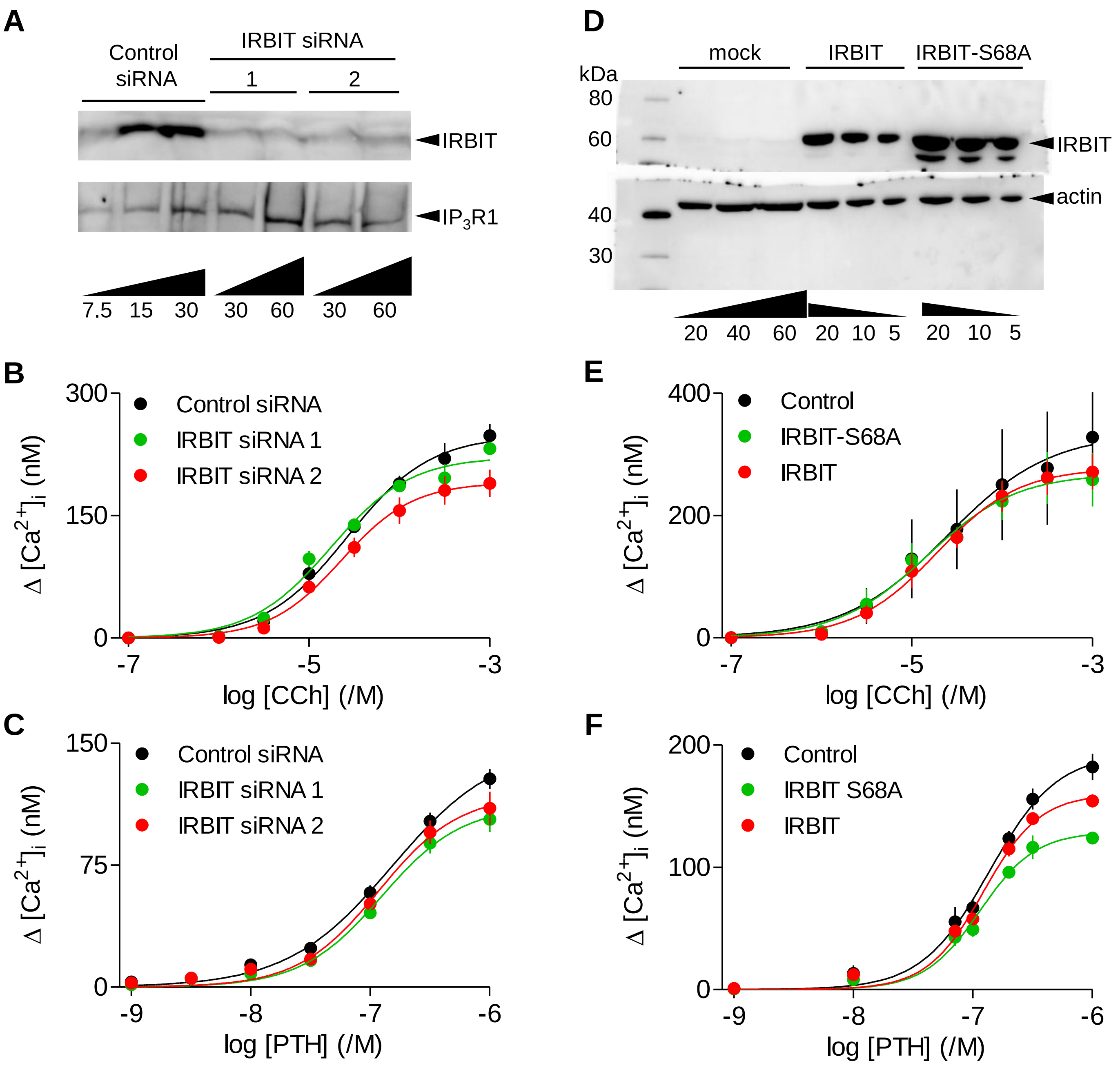

Figure S2. IRBIT Does Not Mediate the Effects of PTH on CCh-Evoked Ca^{2+} Release. Related to Figures 1-4.

(A) WB (taken from the same gel) show expression of IP_3R1 and IRBIT in HEK-PR1 cells transfected with control siRNA or 2 different siRNA against IRBIT. The amounts of protein loaded are shown beneath each lane (µg). Similar results (showing ~90% loss of IRBIT expression after treatment with either effective siRNA) were obtained in at least 3 WB. Neither the control siRNA nor mock transfection affected IRBIT expression. Expression of IP_3R1 was unaffected by IRBIT siRNA.
(B, C) Effects of siRNA treatment on CCh-evoked Ca^{2+} release (B) and the response to PTH added 2 min after CCh (1 mM) (C). Results (mean ± SEM, n = 3) show peak increases in $[Ca^{2+}]_i$ ($\Delta[Ca^{2+}]_i$).
(D) WB (taken from the same gel), typical of 3 similar WB, shows expression of β-actin, IRBIT and IRBIT-S68A in HEK-PR1 cells after transduction with baculovirus. Protein loadings (µg) are shown beneath each lane. M_r markers (kDa) are shown. Parallel transductions of cells with EGFP-IRBIT confirmed that >90% of cells expressed the protein.
(E, F) Effects of over-expressing IRBIT or IRBIT-S68A on CCh-evoked Ca^{2+} release (E) and the response to PTH added 2 min after CCh (1 mM) (F). Results are mean ± SEM, n = 3.

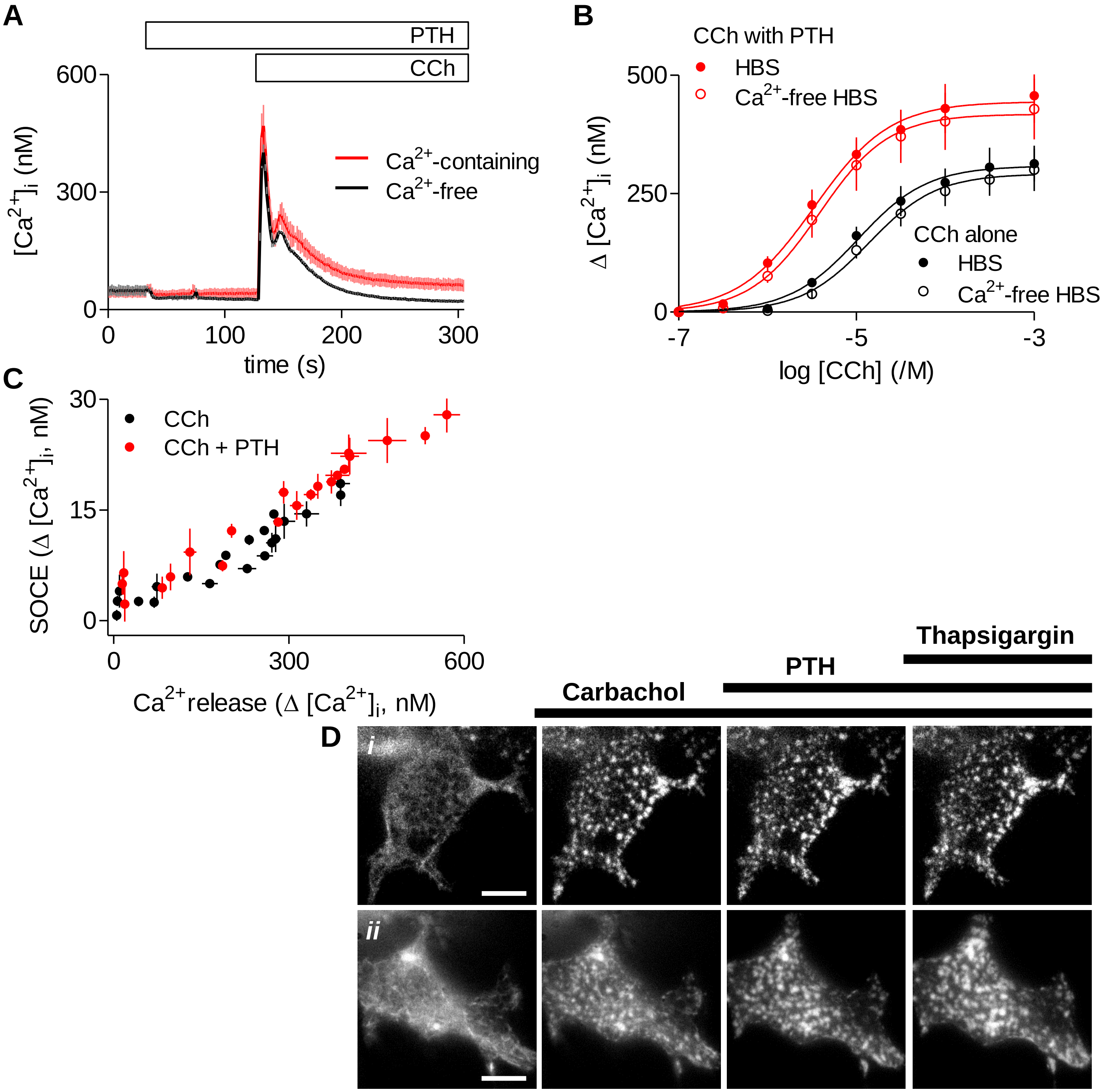

Figure S3. Store-Depletion Evoked by CCh or CCh With PTH Similarly Stimulate SOCE and Translocation of STIM1.
Related to Figures 1-4.

(A) Typical responses from HEK-PR1 cells stimulated with CCh after PTH (100 nM, 1 min) in either HBS or Ca^{2+}-free HBS. Mean ± SD, for 3 replicates for each trace.
(B) Summary results show the peak increase in $[Ca^{2+}]_i$ ($\Delta[Ca^{2+}]_i$).
(C) The sustained increase in $[Ca^{2+}]_i$ in normal HBS was determined 2.5 min after CCh addition (SOCE) and plotted against the initial peak increase in $[Ca^{2+}]_i$ (Ca^{2+} release) for each CCh concentration alone or with PTH. Results (B and C) show mean ± SEM, n = 3.
(D) Representative TIRFM images of HEK-PR1 cells expressing mCh-STIM1 and stimulated with carbachol (1 mM), PTH (100 nM) and then thapsigargin (1 μM) in Ca^{2+}-free HBS (*i*) or HBS (*ii*). Simultaneous recordings of $[Ca^{2+}]_i$ confirmed that each stimulus evoked the expected Ca^{2+} signal. However, the formation of STIM1 puncta varied considerably between cells. The results provided no clear evidence that PTH evoked formation of puncta that were spatially distinct from those evoked by carbachol. Scale bars = 10 μm.

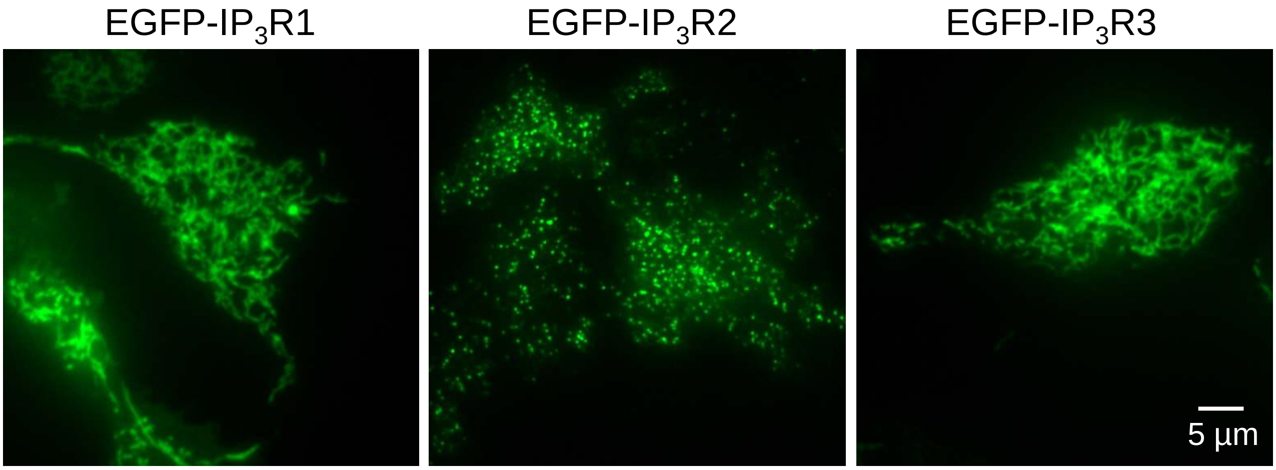

Figure S4. Distribution of GFP-tagged IP_3Rs in HEK-293 Cells.
Related to Figure 7.

Typical total internal reflection fluorescence (TIRF) images of HEK293 cells expressing EGFP-IP_3R1, EGFP-IP_3R2 or EGFP-IP_3R3.
