# Supplementary material for: Cyclic AMP Recruits a Discrete Intracellular Ca2+ Store by Unmasking Hypersensitive IP3 Receptors
Source: Cell Rep. 2017 Jan 17;18(3):711–22. doi: 10.1016/j.celrep.2016.12.058 (PMC5276804; doi:10.1016/j.celrep.2016.12.058)
Supplement: Document S2. Article plus Supplemental Information [file mmc2.pdf]

Cell Reports

Article

Cyclic AMP Recruits a Discrete Intracellular Ca^{2+} Store by Unmasking Hypersensitive IP_3 Receptors

Graphical Abstract

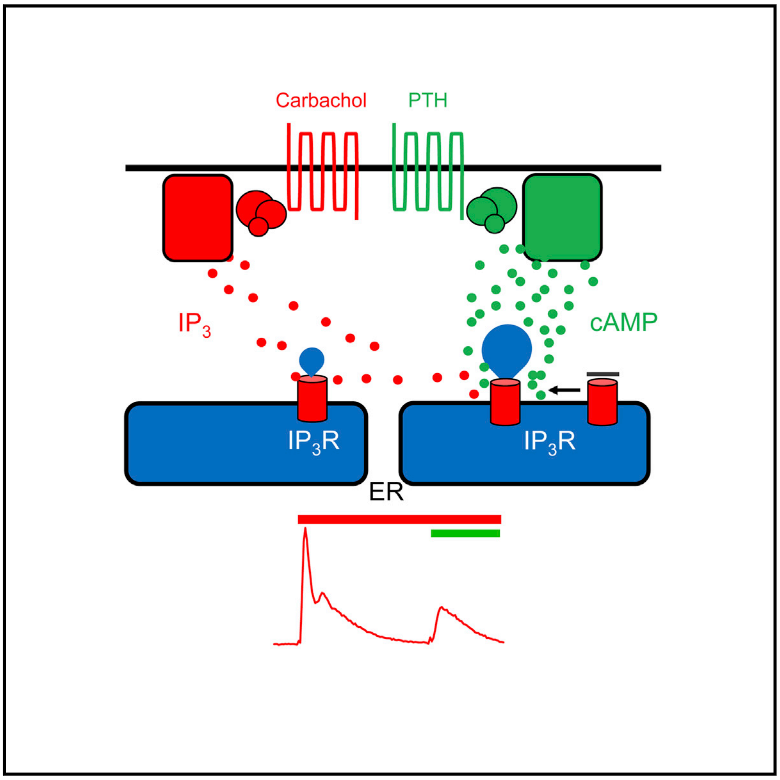

Authors

Vera Konieczny, Stephen C. Tovey, Stefania Mataragka, David L. Prole, Colin W. Taylor

Correspondence

cwt1000@cam.ac.uk

In Brief

Cyclic AMP directly potentiates IP_3-evoked Ca^{2+} release. Konieczny et al. find that IP_3 alone and IP_3 with cAMP release Ca^{2+} from independent stores within the endoplasmic reticulum. Compartmentalized Ca^{2+} stores increase the versatility of IP_3-mediated Ca^{2+} signaling.

Highlights

- Cyclic AMP directly potentiates IP_3-evoked Ca^{2+} release
- The Ca^{2+} stores released by IP_3 alone or IP_3 with cAMP are functionally independent
- Cyclic AMP unmasks high-affinity IP_3 receptors in a discrete ER Ca^{2+} store
- Independent regulation of discrete Ca^{2+} stores increases signaling versatility

CrossMark

Konieczny et al., 2017, Cell Reports *18*, 711–722
January 17, 2017 © 2017 The Author(s).
http://dx.doi.org/10.1016/j.celrep.2016.12.058

CellPress

Cyclic AMP Recruits a Discrete Intracellular Ca^{2+} Store by Unmasking Hypersensitive IP_3 Receptors

Vera Konieczny,[1] Stephen C. Tovey,[1] Stefania Mataragka,[1] David L. Prole,[1] and Colin W. Taylor[1,2,*]
[1]Department of Pharmacology, University of Cambridge, Tennis Court Road, Cambridge CB2 1PD, UK
[2]Lead Contact
*Correspondence: cwt1000@cam.ac.uk
http://dx.doi.org/10.1016/j.celrep.2016.12.058

SUMMARY

Inositol 1,4,5-trisphosphate (IP_3) stimulates Ca^{2+} release from the endoplasmic reticulum (ER), and the response is potentiated by 3′,5′-cyclic AMP (cAMP). We investigated this interaction in HEK293 cells using carbachol and parathyroid hormone (PTH) to stimulate formation of IP_3 and cAMP, respectively. PTH alone had no effect on the cytosolic Ca^{2+} concentration, but it potentiated the Ca^{2+} signals evoked by carbachol. Surprisingly, however, the intracellular Ca^{2+} stores that respond to carbachol alone could be both emptied and refilled without affecting the subsequent response to PTH. We provide evidence that PTH unmasks high-affinity IP_3 receptors within a discrete Ca^{2+} store. We conclude that Ca^{2+} stores within the ER that dynamically exchange Ca^{2+} with the cytosol maintain a functional independence that allows one store to be released by carbachol and another to be released by carbachol with PTH. Compartmentalization of ER Ca^{2+} stores adds versatility to IP_3-evoked Ca^{2+} signals.

INTRODUCTION

G-protein-coupled receptors (GPCRs) comprise the largest class of cell-surface receptors, and they endow cells with the ability to respond to diverse extracellular stimuli. However, most signaling from GPCRs proceeds through a very small number of intracellular messengers, among which 3′,5′-cyclic AMP (cAMP) and Ca^{2+} are the most prominent. GPCRs evoke cAMP formation by stimulating adenylyl cyclases (ACs), whereas most GPCR-evoked Ca^{2+} signals result from stimulation of phospholipase C (PLC) and formation of inositol 1,4,5-trisphosphate (IP_3). IP_3 then evokes Ca^{2+} release from the endoplasmic reticulum (ER) through IP_3 receptors (IP_3Rs) (Figure 1A) (Foskett et al., 2007; Prole and Taylor, 2016). At least three features contribute to specificity within these convergent GPCR signaling pathways. First, individual cells express only a few of the hundreds of GPCRs encoded by the human genome. Most cells are therefore insensitive to most stimuli that activate GPCRs. Second, regulation of many of the signaling proteins, notably ACs and IP_3Rs, is polymodal. The proteins therefore respond optimally only when combinations of stimuli are presented together (Prole and Taylor, 2016; Willoughby and Cooper, 2007). Finally, signaling pathways are spatially organized, often with the aid of scaffold proteins, to allow targeted delivery of diffusible messengers to specific subcellular locations (Delmas et al., 2002; Konieczny et al., 2012; Tu et al., 1998; Willoughby and Cooper, 2007).

IP_3Rs can be phosphorylated by cAMP-dependent protein kinase (PKA) and, at least for IP_3R1 and IP_3R2, this increases their IP_3 sensitivity (Betzenhauser and Yule, 2010; Masuda et al., 2010). We and others have shown that cAMP can also potentiate IP_3-evoked Ca^{2+} signals by a mechanism that requires neither of the usual targets of cAMP, PKA and exchange proteins activated by cAMP (EPACs) (Figure 1A) (Kurian et al., 2009; Tovey et al., 2008, 2010). This potentiation is due to enhanced Ca^{2+} release by IP_3Rs, rather than to inhibition of Ca^{2+} removal from the cytosol (Tovey et al., 2003). We have provided evidence that cAMP is delivered directly to IP_3Rs within junctions formed between IP_3R2 and AC6, and that within these junctions the local concentration of cAMP is more than sufficient to fully potentiate responses to IP_3 (Figure 1A) (Tovey et al., 2008). We proposed that each junction works as a digital "on-off switch," with more switches flicked as more AC-coupled receptors are activated (Tovey et al., 2008).

In the present study, we show that cAMP unmasks IP_3Rs within an ER Ca^{2+} store that is functionally distinct from the store released by IP_3 alone. Our results suggest a remarkable independence of the ER Ca^{2+} stores released by IP_3 alone or IP_3 combined with cAMP, and they thereby reveal an additional source of versatility within these signaling pathways.

RESULTS AND DISCUSSION

Ca^{2+} Signals Evoked by Stimuli that Cause Very Different Increases in Intracellular Free Ca^{2+} Concentration Are Uniformly Enhanced by PTH

In Ca^{2+}-free HEPES-buffered saline (HBS), carbachol (CCh) evoked a concentration-dependent increase in $[Ca^{2+}]_i$ (intracellular free Ca^{2+} concentration) (pEC_{50} = 4.60 ± 0.07, where pEC_{50} = −log of the half-maximally effective concentration) in HEK cells stably expressing type 1 human parathyroid hormone (PTH) receptor (HEK-PR1 cells) (Figures 1B and 1C). This is consistent with evidence that the endogenous M_3

This is an open access article under the CC BY license (http://creativecommons.org/licenses/by/4.0/).

OPEN
ACCESS
CellPress

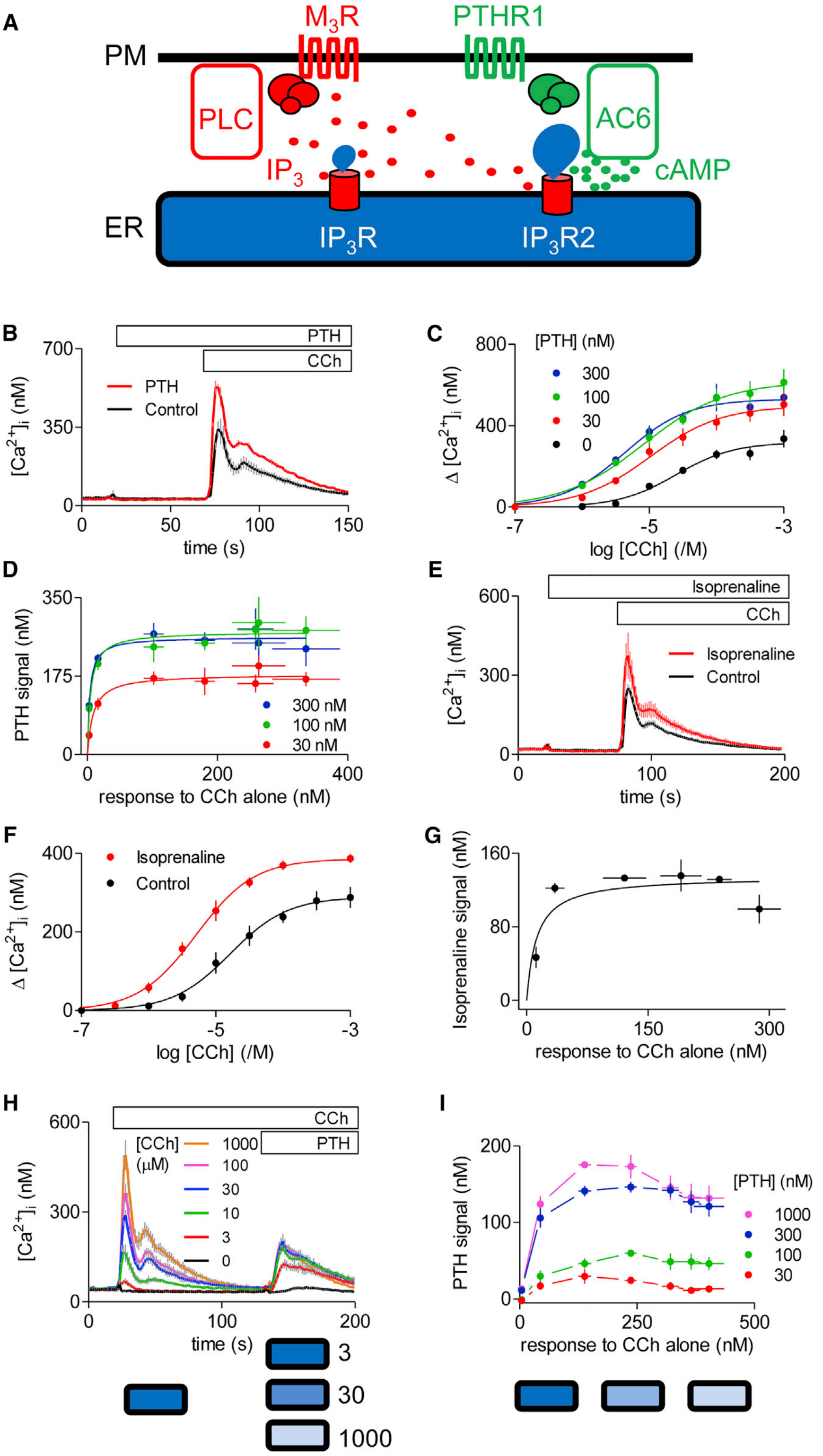

Figure 1. Potentiation of CCh-Evoked Ca^{2+} Signals by PTH and Isoprenaline

(A) CCh through type 3 muscarinic acetylcholine receptors (M_3Rs) stimulates phospholipase C (PLC) and formation of IP_3, which stimulates Ca^{2+} release from the ER through IP_3Rs. Stimulation of PTHR1 with PTH activates adenylyl cyclase (AC). The cAMP produced potentiates the Ca^{2+} release evoked by IP_3. A specific association of AC6 with IP_3R2 allows cAMP to be delivered at high concentrations to IP_3Rs.

(B) Typical results (mean ± SD from three wells) for HEK-PR1 cells stimulated with PTH (100 nM) and then CCh (1 mM) in Ca^{2+}-free HBS.

(C) Summary results show concentration-dependent effects of CCh alone or after pre-incubation (1 min) with the indicated concentrations of PTH on the increase in $[Ca^{2+}]_i$ ($\Delta[Ca^{2+}]_i$).

(D) From the results shown in (C), the increase in $[Ca^{2+}]_i$ evoked by each CCh concentration alone was subtracted from the response evoked by the same CCh concentration with PTH. The $[Ca^{2+}]_i$ increase due to PTH is plotted against that evoked by CCh alone.

(E) Typical results (mean ± SD from three wells) for HEK293 cells stimulated with isoprenaline (10 μM) and then CCh (1 mM) in Ca^{2+}-free HBS.

(F) Summary results show the concentration-dependent effects on $\Delta[Ca^{2+}]_i$ of CCh alone or after pre-incubation (1 min) with isoprenaline (10 μM).

(G) $\Delta[Ca^{2+}]_i$ due to isoprenaline is plotted against that evoked by CCh alone.

(H) Typical responses (mean ± SD from three wells) for HEK-PR1 cells stimulated with the indicated concentrations of CCh before addition of PTH (300 nM).

(I) Summary results show the responses evoked by PTH plotted against the increase in $[Ca^{2+}]_i$ evoked by the prior stimulation with CCh. Bottom diagrams in (H) and (I) represent the global Ca^{2+} content of the ER at the time of stimulus addition (darker tones indicate fuller stores, and the numbers alongside represent CCh concentrations in μM).

Results are means ± SEM, n ≥ 3 (C, D, F, G, and I). See also Figures S2 and S3.

muscarinic acetylcholine receptors (M_3R) of HEK293 cells stimulate Ca^{2+} release from intracellular stores through IP_3Rs (Tovey et al., 2008). Neither isoprenaline, which stimulates endogenous β_2-adrenoceptors, nor PTH evoked an increase in $[Ca^{2+}]_i$. However, pre-treatment with PTH or isoprenaline potentiated the increase in $[Ca^{2+}]_i$ evoked by maximal and submaximal concentrations of CCh (Figures 1B–1G). These results are consistent with previous reports showing that cAMP potentiates IP_3-evoked Ca^{2+} signals in HEK293 cells (Kurian et al., 2009; Meena et al., 2015; Tovey et al., 2008) (Figure 1A).

Figure 1D compares the amplitudes of the Ca^{2+} signals evoked by CCh alone with the amplitude of the additional increase in $[Ca^{2+}]_i$ because of pre-treatment with a maximal

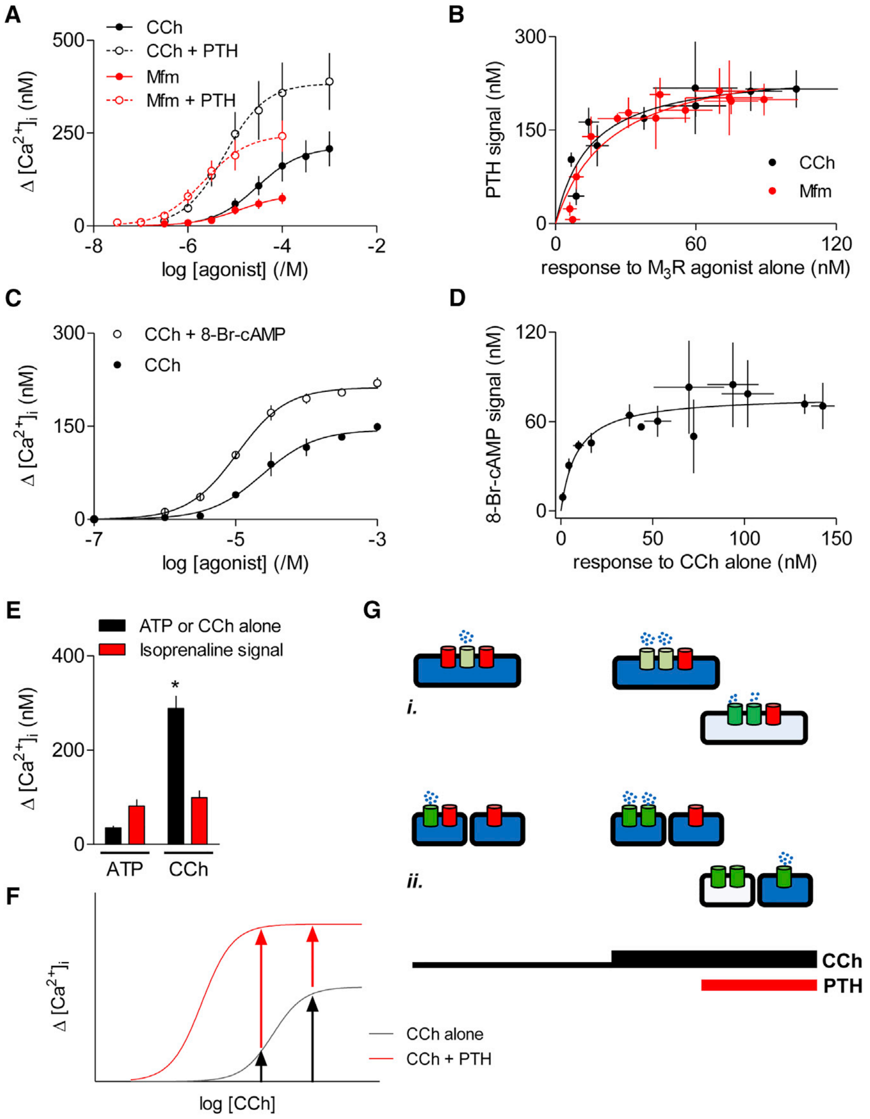

Figure 2. cAMP Evokes Similar Ca^{2+} Signals after Stimuli That Alone Evoke Very Different Increases in $[Ca^{2+}]_i$

(A) Methods similar to those shown in Figure 1B were used to assess the effects on $[Ca^{2+}]_i$ of the indicated concentrations of CCh or Mfm alone, or after pre-incubation with PTH (100 nM, 1 min).

(B) $\Delta[Ca^{2+}]_i$ due to PTH is plotted against that evoked by CCh or Mfm alone.

(C) Effects on $\Delta[Ca^{2+}]_i$ in HEK-PR1 cells of CCh alone or CCh after pre-incubation with 8-Br-cAMP (10 mM, 20 min).

(D) $\Delta[Ca^{2+}]_i$ due to 8-Br-cAMP is plotted against that evoked by CCh alone.

(E) Similar analyses of HEK293 cells stimulated with ATP (300 μM) or CCh (1 mM) alone, or after pre-incubation with isoprenaline (10 μM, 1 min). The maximal amplitudes of the Ca^{2+} signals evoked by CCh or ATP alone, and the additional effect of isoprenaline are shown as means ± SEM, n ≥ 3. *$p < 0.05$, Student's t test, for CCh compared with ATP.

(F) Expected effects of PTH on CCh-evoked Ca^{2+} signals assuming that IP_3 is uniformly delivered to all IP_3Rs made more sensitive to IP_3 by cAMP. Previous work established that even maximal activation of M_3Rs in HEK-PR1 cells generates insufficient IP_3 to activate all IP_3Rs (Tovey et al., 2008), hence the increased maximal response to CCh in the presence of PTH.

(G) The similar Ca^{2+} signals evoked by CCh with PTH after CCh alone has evoked Ca^{2+} signals with very different amplitudes might be because of Ca^{2+} release from a uniform ER, with the increased sensitivity of more IP_3Rs compensating for the diminished ER Ca^{2+} content (i). Alternatively, CCh alone and CCh with PTH may evoke Ca^{2+} release through IP_3Rs resident in different stores (ii).

See also Figures S2 and S3.

(100 nM) or submaximal (30 nM) concentration of PTH. The results demonstrate that for CCh concentrations that evoked Ca^{2+} signals of very different amplitudes (~20–340 nM), the additional increase in $[Ca^{2+}]_i$ evoked by PTH was almost invariant, but larger for the maximal concentration of PTH ($\Delta[Ca^{2+}]_i$ ~240 nM) than for the submaximal PTH concentration ($\Delta[Ca^{2+}]_i$ ~170 nM) (Figure 1D). Similar results were obtained when the cells were first stimulated with CCh and then with PTH after $[Ca^{2+}]_i$ had returned to its basal level (Figures 1H and 1I). The reduced sensitivity to PTH in this second protocol is probably due to the briefer exposure to PTH, which is likely to equilibrate slowly with its receptors.

5-Methylfurmethiodide (Mfm) is a partial agonist of M_3Rs: the maximal increase in $[Ca^{2+}]_i$ evoked by Mfm was only 36% ± 1% of that evoked by CCh (Figure 2A). Nevertheless, the amplitude of the additional Ca^{2+} signal evoked in the presence of PTH was similar across most concentrations of Mfm, and also similar to that evoked by PTH with CCh (Figures 2A and 2B). Similar results were obtained when cells were pre-treated with 8-bromo cAMP (8-Br-cAMP), rather than PTH, and then stimulated with CCh (Figures 2C and 2D). In HEK293 cells, ATP through P2Y receptors also evoked an increase in $[Ca^{2+}]_i$, but the maximal amplitude of the Ca^{2+} signal was only 13% ± 2% of that evoked by CCh in the same cells (Figure 2E). Nevertheless, the additional Ca^{2+} signal evoked by isoprenaline was similar for maximally effective concentrations of CCh and ATP (Figure 2E).

The similar effect of PTH (and of other cAMP-elevating stimuli) across most CCh concentrations (Figure 1) is unexpected because if PTH uniformly increased the sensitivity of IP_3Rs to IP_3, its effects should be most pronounced at the lowest CCh concentrations (Figure 2F). The same argument applies to the results with other submaximal responses to Ca^{2+}-mobilizing stimuli (Figures 2A–2E). It might be argued that the similar effect of a maximal PTH concentration on the Ca^{2+} signals evoked by most CCh concentrations is due to PTH causing an increase in IP_3R sensitivity sufficient for each CCh concentration to evoke maximal Ca^{2+} release. However, that explanation cannot easily be reconciled with

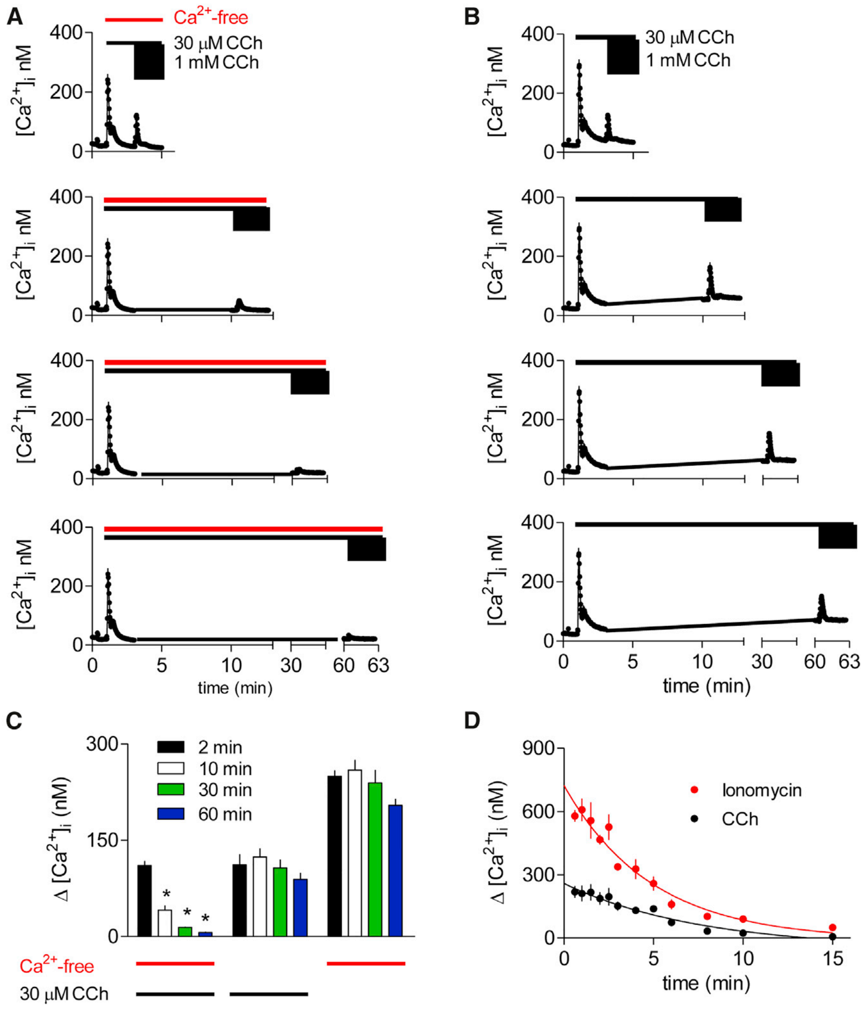

Figure 3. Depletion of the CCh-Sensitive Ca^{2+} Stores by Sustained Submaximal Stimulation with CCh

(A and B) Populations of HEK-PR1 cells in either Ca^{2+}-free HBS (A) or normal HBS (B) were stimulated with 30 μM CCh for the indicated times before addition of 1 mM CCh. Typical results show means ± SD from three replicates in each trace.

(C) Summary results (mean ± SEM, from three independent experiments) show peak increases in $[Ca^{2+}]_i$ evoked by 1 mM CCh after pre-incubation with 30 μM CCh in Ca^{2+}-free or Ca^{2+}-containing HBS, or without prior stimulation with CCh in Ca^{2+}-free HBS for the indicated times. $^*p < 0.05$, one-way ANOVA and Tukey's post hoc test, relative to measurements at 2 min.

(D) HEK-PR1 cells in Ca^{2+}-free HBS were stimulated with thapsigargin (1 μM), and at intervals thereafter the effects of CCh (1 mM) or ionomycin (10 μM) were determined. Results are mean ± SEM from three independent experiments.

See also Figures S2 and S3.

the observation that the response to PTH is similar after stores have been minimally or substantially depleted of Ca^{2+} by prior treatment with CCh (bottom diagrams in Figures 1H and 1I). Nor could it account for the uniform effect of a submaximal PTH concentration, which also had similar effects across most CCh concentrations, although less than those of the maximal PTH concentration (Figures 1D and 1I).

These results demonstrate that for stimuli that evoke very different increases in $[Ca^{2+}]_i$, the additional Ca^{2+} release evoked by PTH (or isoprenaline) is similar. We can envisage two possible explanations for these observations. It may be that all stimuli release Ca^{2+} from a shared Ca^{2+} store, and the consistent responses to PTH then reflect a balance, as the CCh concentration increases, between the declining content of the Ca^{2+} store and a compensating increase in the sensitivity of a larger number of IP_3Rs (Figure 2Gi). That fortuitous balance would need to hold across a diverse array of stimulus combinations and intensities, and between cell lines (Figures 1 and 2). Alternatively, CCh alone and CCh with PTH may release Ca^{2+} from different intracellular stores (Figure 2Gii). Subsequent experiments seek to distinguish between these possibilities.

Depletion of the CCh-Sensitive Ca^{2+} Stores Does Not Affect Responses to PTH

During prolonged incubation of HEK-PR1 cells with a half-maximally effective concentration of CCh (30 μM) in Ca^{2+}-free HBS, the increase in $[Ca^{2+}]_i$ evoked by subsequent addition of a maximal CCh concentration (1 mM) decreased with time (half-time for loss of response, $t_{½}$ = 5 ± 1 min) (Figure 3A). After a 60 min incubation with 30 μM CCh, the response to maximal stimulation declined to 6% ± 1% of that recorded after a 2 min incubation. However, after a 60 min incubation in Ca^{2+}-free HBS without CCh, the response to 1 mM CCh was reduced to 82% ± 4% of the initial response, and after a 60 min incubation with 30 μM CCh in Ca^{2+}-containing HBS, the response to subsequent addition of 1 mM CCh was 83% ± 13% of the initial response (Figures 3B and 3C).

We considered whether the response to stimulation with a maximal concentration of CCh might fail to directly report the Ca^{2+} content of the CCh-sensitive stores. If, for example, IP_3Rs were regulated by luminal Ca^{2+}, then CCh-evoked Ca^{2+} release might terminate before the stores were empty. However, when cells were treated with 1 μM thapsigargin to inhibit the sarcoplasmic/endoplasmic reticulum Ca^{2+}-ATPase (SERCA) and so unmask a Ca^{2+} leak from the ER, the rates of decline of the response to 1 mM CCh ($t_{½}$ = 3.8 ± 0.4 min, n = 3) and of the Ca^{2+} content of the stores assessed by addition of 1 μM ionomycin ($t_{½}$ = 3.0 ± 0.3 min) were indistinguishable (Figure 3D). Together these results demonstrate that sustained stimulation with a submaximal concentration of CCh depletes the intracellular stores from which CCh releases Ca^{2+}.

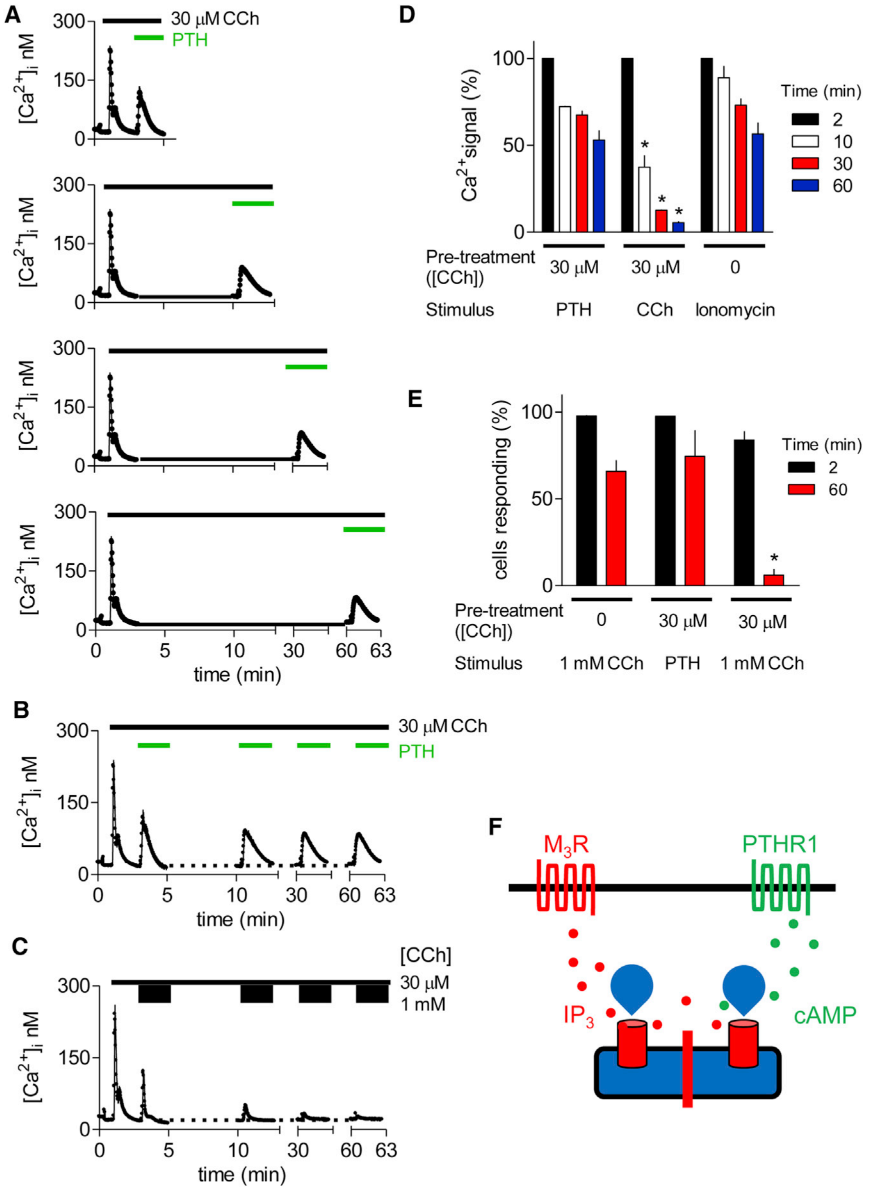

Figure 4. PTH Evokes Ca^{2+} Release after Depletion of CCh-Sensitive Ca^{2+} Stores

(A) HEK-PR1 cells in Ca^{2+}-free HBS were stimulated with 30 μM CCh for the indicated times before addition of 100 nM PTH. Typical results show means ± SD from three replicates in each trace.

(B) Traces from the four experiments shown in (A) are reproduced in this single panel.

(C) Similar representation of the results from Figure 3A.

(D) Summary shows the peak increases in $[Ca^{2+}]_i$ evoked by 1 mM CCh or 100 nM PTH after the indicated periods in Ca^{2+}-free HBS with 30 μM CCh, and the responses to 1 μM ionomycin after the indicated periods in Ca^{2+}-free HBS alone. Results (mean ± SEM, n = 3) are normalized to the Ca^{2+} signals measured after 2 min. *$p < 0.05$, one-way ANOVA and Tukey's post hoc test, relative to time-matched response to ionomycin.

(E) Single HEK-PR1 cells were stimulated continuously with 30 μM CCh in Ca^{2+}-free HBS and then with PTH (100 nM) or CCh (1 mM) after the indicated intervals. Results (mean ± SEM, from three experiments with at least 53 cells analyzed in each) show the percentage of responsive cells. *$p < 0.05$, one-way ANOVA and Tukey's post hoc test, relative to measurement at 2 min.

(F) The results suggest that CCh and CCh with PTH evoke Ca^{2+} release from independent Ca^{2+} stores.

See also Figures S1–S3.

Addition of PTH (100 nM) to HEK-PR1 cells stimulated for 2 min with 30 μM CCh in Ca^{2+}-free HBS evoked an increase in $[Ca^{2+}]_i$ ($\Delta[Ca^{2+}]_i$ = 92 ± 10 nM) similar to that evoked by addition of 1 mM CCh (110 ± 7 nM) (Figures 4A–4C). However, whereas sustained stimulation with 30 μM CCh effectively abolished the response to subsequent addition of 1 mM CCh, it had very little effect on the response to PTH (Figures 4B and 4C). The modest decline in the response to PTH matched the slow decline of the Ca^{2+} content of the stores in Ca^{2+}-free HBS without CCh (determined by addition of ionomycin; Figure 4D). Similar results, namely loss of the response to a maximal concentration of CCh alone and unperturbed responses to PTH, were observed when areas under the Ca^{2+} responses, rather than peak increases in $[Ca^{2+}]_i$, were analyzed (data not shown). Analysis of single HEK-PR1 cells using the same protocol established that the very different effects of depleting CCh-sensitive Ca^{2+} stores on subsequent responses to CCh or PTH were not due to cellular heterogeneity (Figure 4E).

PTH can, particularly when its receptors are overexpressed, stimulate formation of IP_3 (He et al., 2015; Taylor and Tovey, 2012). However, we showed previously that PTH does not stimulate IP_3 formation in HEK-PR1 cells (Meena et al., 2015; Short and Taylor, 2000), and others have shown that potentiation of M_3R-evoked Ca^{2+} signals by activation of β_2-adrenoceptors occurs without formation of additional IP_3 (Kurian et al., 2009). Our conclusion that the effects of PTH are not mediated by formation of additional IP_3 is further confirmed by the present results showing that PTH evokes Ca^{2+} release under conditions where increasing IP_3 formation, by increasing the CCh concentration, is ineffective (Figures 4B and 4C).

Hence, although PTH evokes Ca^{2+} release only when there is coincident activation of M_3Rs by CCh, the Ca^{2+} stores released by CCh alone and by CCh with PTH are largely independent (Figure 4F). We suggested a similar conclusion previously, albeit with less decisive evidence, from results showing that depleting membranes of cholesterol selectively abolished the Ca^{2+} signals evoked by CCh without affecting those evoked by CCh with PTH (Tovey and Taylor, 2013).

PTH-Evoked Ca^{2+} Release Requires Continuous Activation of M_3Rs

Methylatropine is a competitive antagonist of M_3Rs and, as expected, it abolished the Ca^{2+} signals evoked by CCh (data not shown). During sustained exposure to 30 μM CCh, the increase in $[Ca^{2+}]_i$ evoked by subsequent addition of PTH was abolished when methylatropine was added with the PTH (Figures S1A–S1C). Neither CCh nor methylatropine affected the amount of cAMP produced in response to PTH (Figure S1D). These results suggest three conclusions. They demonstrate that the response to PTH requires ongoing activation of M_3Rs and is not a long-lasting consequence of their prior activation. They indicate that every step in the signaling pathway linking M_3Rs to activation of IP_3Rs is rapidly reversed when CCh can no longer reassociate with M_3Rs. This second conclusion is consistent with rapid degradation of IP_3 in cells ($t_{½} \leq 10$ s) (Fink et al., 1999; Matsuura et al., 2006; Wang et al., 1995), and it suggests rapid termination of all preceding steps in the signaling pathway, including G protein de-activation and dissociation of IP_3 from IP_3Rs. Finally, the results demonstrate that there is no desensitization of M_3Rs during sustained incubations with CCh.

In rat basophilic leukemia cells, store-operated Ca^{2+} entry (SOCE) is required for resynthesis of the pool of phosphatidylinositol 4,5-bisphosphate that sustains IP_3 production during activation of leukotriene receptors (Alswied and Parekh, 2015). There appears to be no such requirement for SOCE in HEK-PR1 cells, because throughout a 60 min stimulation with a submaximal concentration of CCh in the absence of extracellular Ca^{2+}, the formation of IP_3 was sustained (Figures 4B and S1A–S1C).

PTH Recruits More Sensitive IP_3Rs

After sustained stimulation of HEK-PR1 cells with a submaximal (30 μM) or maximal (1 mM) concentration of CCh to deplete the CCh-sensitive Ca^{2+} stores, the subsequent response to PTH was the same for both CCh concentrations (Figure 5A). These results extend those shown in Figure 4 by demonstrating that even sustained (60 min) stimulation with a maximally effective CCh concentration has no effect on the subsequent response to PTH. Furthermore, the results demonstrate that a low CCh concentration is as effective as a maximal CCh concentration in allowing PTH to evoke Ca^{2+} signals. This suggests that the IP_3Rs recruited by PTH are more sensitive to IP_3 than those responding to CCh alone.

The apparent independence of the Ca^{2+} stores released by CCh alone or CCh with PTH (Figure 4F) allowed us to directly determine the CCh sensitivity of the two stores using the protocol shown in Figure 5B. This involved depleting the CCh-sensitive stores by sustained stimulation in Ca^{2+}-free HBS, washing the cells, and then determining their sensitivity to CCh with PTH. Under these conditions, there was no response to CCh or PTH alone, but CCh with PTH stimulated Ca^{2+} release (Figures 5B and 5C). To determine the sensitivity of the stores that respond to CCh alone, the stores were allowed to refill with Ca^{2+} by incubation in normal HBS during the washing period and subsequent stimulation with CCh. The comparison is valid because Ca^{2+} entry does not contribute to the peak Ca^{2+} signals evoked by CCh or CCh with PTH (see Figure S3B). The results demonstrate that PTH causes a concentration-dependent increase in the maximal response (Figure 5D), and that the stores responding to CCh with PTH are more sensitive to CCh than those responding to CCh alone (Figure 5E). We conclude that PTH causes a concentration-dependent unmasking of IP_3Rs within a discrete Ca^{2+} store, and that these unmasked IP_3Rs have enhanced sensitivity to CCh (Figure 5F). We showed previously, using small interfering RNA (siRNA), that in HEK-PR1 cells responses to CCh alone were most affected by loss of IP_3R1, whereas responses to CCh with PTH were most affected by loss of IP_3R2 (Tovey et al., 2008). Hence, our conclusion that PTH unmasks sensitive IP_3Rs aligns with evidence that IP_3R2, the most sensitive IP_3R subtype (Iwai et al., 2005), is selectively regulated by PTH.

The functional independence of the Ca^{2+} stores released by CCh alone or CCh with PTH (Figure 4F) implies that IP_3Rs in the stores responding to CCh alone are insensitive to cAMP. We speculated previously that association of these IP_3Rs with M_3R signaling pathways might allow local delivery of IP_3 at concentrations more than sufficient for their maximal activation, thereby depriving the IP_3Rs of any additional benefit from cAMP (Tovey and Taylor, 2013). However, this explanation now seems unlikely because we have found no evidence that CCh causes local saturation of IP_3Rs with IP_3 (Konieczny, 2015). Our new results, suggesting that PTH unmasks IP_3Rs within a distinct Ca^{2+} store, provide a simple explanation for the lack of effect of PTH on the Ca^{2+} stores that respond to CCh alone, because their IP_3Rs are already accessible to IP_3.

The results so far prompt experiments designed to address the mechanism by which PTH (through cAMP) unmasks IP_3Rs and the means by which two intracellular Ca^{2+} stores maintain their functional independence.

IRBIT Is Unlikely to Mediate the Effect of PTH on Ca^{2+} Signals

The phosphoprotein, IRBIT (IP_3R-binding protein released by IP_3), is an endogenous IP_3R antagonist (Ando et al., 2003; Devogelaere et al., 2006) that is expressed in HEK293 cells (Kiefer et al., 2009). Because a protein homologous to the C-terminal region of IRBIT, *S*-adenosylhomocysteine-hydrolase (AHCY), binds cAMP (Kloor et al., 2009), IRBIT is a candidate for suppressing IP_3R activity. Furthermore, IRBIT has been implicated in synergistic regulation of fluid secretion by cAMP and IP_3-evoked Ca^{2+} release, where phosphorylation of IP_3Rs by PKA was proposed to facilitate Ca^{2+} release by reciprocally regulating the affinity of IP_3R1 for IP_3 and IRBIT (Park et al., 2013).

Two different siRNAs to IRBIT, which inhibited IRBIT expression by ~90% without affecting expression of IP_3R1 (Figure S2A), had no significant effect on either the concentration-dependent effects of CCh on $[Ca^{2+}]_i$ or the potentiating effect of any PTH concentration (Figures S2B and S2C). We also used baculovirus to achieve high levels of expression of IRBIT or a dominant-negative form (IRBIT-S68A) (Ando et al., 2006) in HEK-PR1 cells (Figure S2D). Expression of these proteins had no effect on the Ca^{2+} signals evoked by CCh alone or CCh with PTH (Figures S2E and S2F). We conclude that IRBIT does not contribute to the effects of PTH on CCh-evoked Ca^{2+} signals.

It is surprising, when endogenous IRBIT has been reported to inhibit IP_3-evoked Ca^{2+} signals in other cells (Ando et al., 2006;

OPEN ACCESS
CellPress

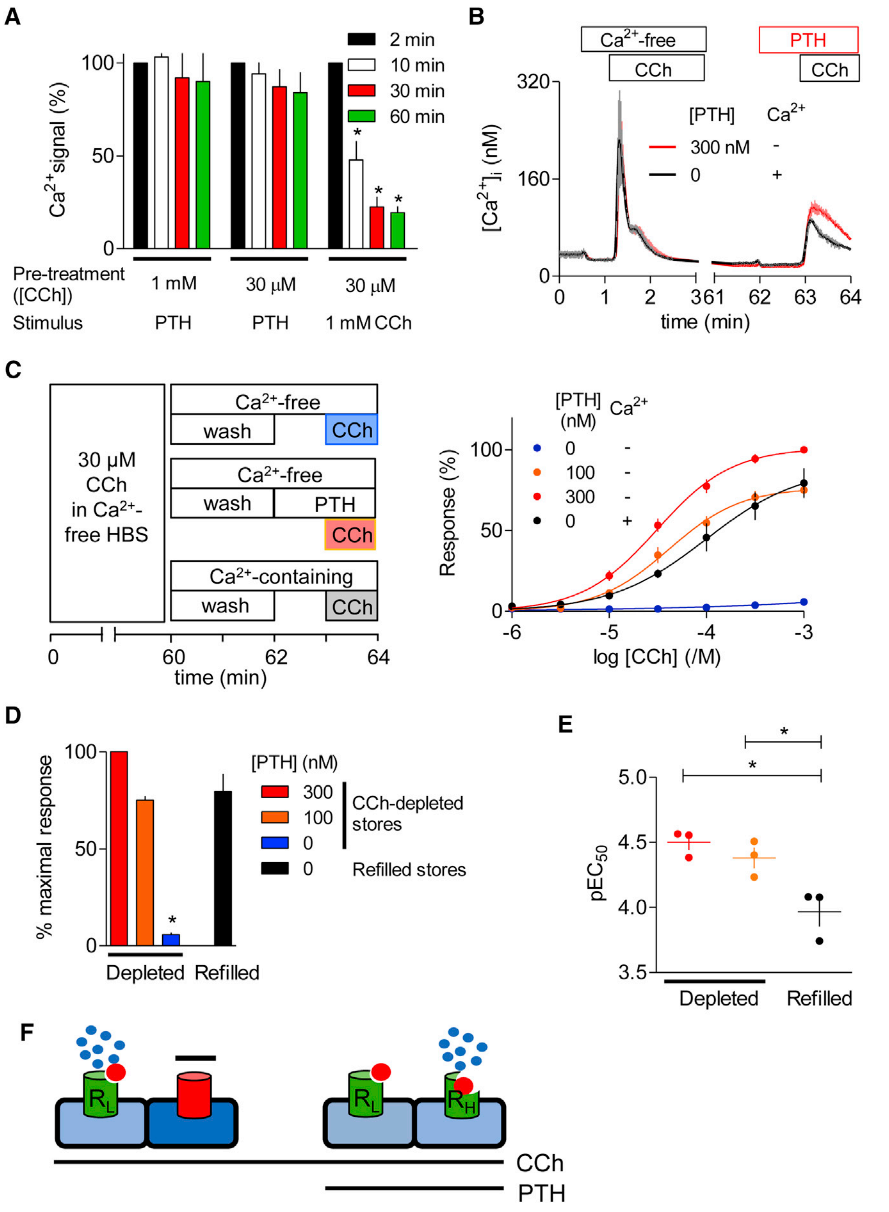

Figure 5. PTH Recruits More Sensitive IP_3Rs

(A) HEK-PR1 cells were pre-stimulated with 30 μM or 1 mM CCh for the indicated periods in Ca^{2+}-free HBS before addition of PTH (100 nM) or CCh (1 mM), as indicated. Results show peak increases in $[Ca^{2+}]_i$ evoked by the final stimulus expressed as a percentage of that evoked when it was presented 2 min after the first addition of CCh. *p < 0.05, one-way ANOVA and Tukey's post hoc test.

(B) The CCh-sensitive Ca^{2+} stores were first emptied by incubating cells for 60 min with 30 μM CCh in Ca^{2+}-free HBS to determine the CCh sensitivity of the stores that respond to CCh alone or CCh with PTH. Cells were then washed in Ca^{2+}-free HBS to remove CCh (2 min) and then stimulated with PTH and CCh in Ca^{2+}-free HBS (to determine the CCh sensitivity of the CCh/PTH-responsive stores). Alternatively, cells were washed in normal HBS (to allow intracellular stores to refill) and then stimulated with CCh (to determine the sensitivity of the CCh-responsive stores). Shown are typical traces for the indicated treatments (mean ± SD for three replicates).

(C) Summary, with the protocol shown alongside, shows the concentration-dependent effects of CCh on the peak increase in $[Ca^{2+}]_i$ after the indicated treatments. Results are normalized to the maximal response evoked by CCh with 300 nM PTH.

(D and E) Summary results show the effects of different concentrations of PTH on the maximal amplitude of the Ca^{2+} signal (D) and the sensitivity (pEC_{50}) to CCh (E).

(F) The results suggest that PTH unmasks IP_3Rs with increased affinity for IP_3 (from R_L to R_H) in a discrete Ca^{2+} store.

Results are mean ± SEM, n = 3 (A and C–E). *p < 0.05, one-way ANOVA and Tukey's post hoc test (D and E).

Devogelaere et al., 2006; Zaika et al., 2011), that neither overexpression of IRBIT nor its inhibition should affect IP_3-evoked Ca^{2+} signals in HEK-PR1 cells (Figure S2). Because IRBIT must be phosphorylated before it can bind to IP_3Rs (Ando et al., 2006; Devogelaere et al., 2007; Kiefer et al., 2009), we suggest that the mechanisms responsible for phosphorylation of IRBIT may be inactive in HEK-PR1 cells. Whatever the explanation for the lack of effect of IRBIT on IP_3-evoked Ca^{2+} release, it seems clear that dissociation of IRBIT from IP_3Rs is not the means by which cAMP unmasks IP_3R activity.

Stores Depleted by CCh or CCh with PTH Are Similarly Effective in Evoking SOCE

SOCE is triggered by loss of Ca^{2+} from the ER, leading to association of stromal interaction molecule 1 (STIM1) and Orai at ER-plasma membrane junctions (Lewis, 2011). Previous work established that, in HEK-PR1 cells, CCh-evoked Ca^{2+} entry is entirely mediated by SOCE (López Sanjurjo et al., 2014). We considered whether the Ca^{2+} stores emptied by CCh or CCh with PTH might differ in their abilities to evoke SOCE. The peak increases in $[Ca^{2+}]_i$ evoked by CCh alone or CCh with PTH were, as expected, entirely mediated by Ca^{2+} release from intracellular stores (Figures S3A and S3B). Comparison of the initial peak increases in $[Ca^{2+}]_i$ evoked by CCh or CCh with PTH (Ca^{2+} release) with the amplitude of the subsequent sustained increase in $[Ca^{2+}]_i$ (SOCE) revealed that the relationship between the two Ca^{2+} signals was indistinguishable for cells stimulated with the different stimuli (Figure S3C). These results, which are also consistent with previous reports that intracellular stores must be substantially depleted of Ca^{2+} before they effectively evoke STIM1 translocation (Suzuki et al., 2014) and activation of SOCE (Bird et al., 2009; Luik et al., 2008), suggest that stores depleted by CCh alone or CCh with PTH are equally capable of stimulating

OPEN ACCESS
CellPress

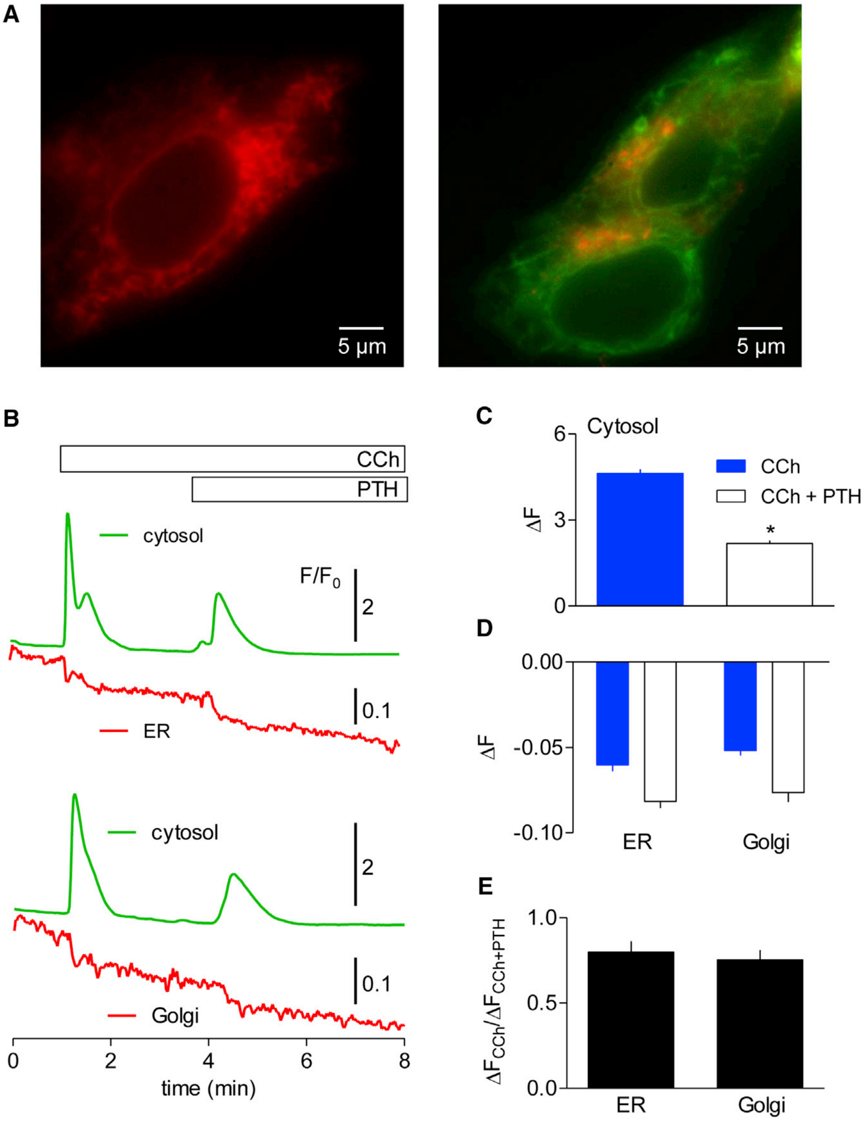

Figure 6. The Golgi Apparatus Is Not the Independent Ca^{2+} Store Recruited by PTH

(A) Typical widefield images of HEK-PR1 cells expressing ER-LAR-GECO1 (left) and Golgi-LAR-GECO1 with GFP-ER (right).

(B) HEK-PR1 cells expressing Ca^{2+} sensors within either the ER or Golgi lumen and loaded with a cytosolic Ca^{2+} indicator (fluo-8) were stimulated as indicated, in Ca^{2+}-free HBS with CCh (1 mM) and PTH (300 nM). Typical traces, each from a single cell, show the simultaneous recordings of cytosolic and luminal fluorescence measured in several regions of interest in each cell (as F/F_0, where F_0 is the average fluorescence recorded for 15 s before any stimulation).

(C and D) Summary results show fluorescence changes for the cytosolic (C) and luminal (D) indicators (as $\Delta F = F_{peak} - F_{pre}$, where F_{peak} is the peak F/F_0 value, and F_{pre} is the value determined immediately before stimulation). The code applies to both panels.

(E) For each cell, the ratio of the fluorescence signals (ΔF) evoked by CCh and PTH is shown for the ER and Golgi sensors. (C–E) Results are mean ± SEM from at least 27 cells. $^{*}p < 0.05$, Student's t test, comparing CCh with CCh and PTH (C) or ER relative to Golgi (D and E).

SOCE. We also considered whether translocation of STIM1 after store depletion might reveal the subcellular location of the Ca^{2+} stores emptied by CCh alone or with PTH. In HEK-PR1 cells expressing mCherry-STIM1, the stimuli evoked formation of STIM1 puncta near the plasma membrane, but there was no discernible difference in the spatial distribution of the puncta formed after stimulation with CCh alone or CCh with PTH (Figure S3D).

The Golgi Apparatus Is Not the Independent Ca^{2+} Store Recruited by PTH

The ER and Golgi apparatus accumulate Ca^{2+}, IP_3 can evoke Ca^{2+} release from both organelles (Pizzo et al., 2011; Rodríguez-Prados et al., 2015; Wong et al., 2013), and recent work suggests that in cardiac myocytes spontaneous Ca^{2+} release through ryanodine receptors in the Golgi apparatus is enhanced by activation of G_s-coupled receptors (Yang et al., 2015). Ca^{2+} accumulation by the Golgi apparatus is mediated by a SERCA and, within the *trans*-Golgi, by a secretory pathway Ca^{2+}-ATPase (SPCA) (Aulestia et al., 2015). Both Ca^{2+} pumps are inhibited by thapsigargin, although SPCAs are less sensitive to thapsigargin than SERCAs (Dode et al., 2006). Because considerable evidence suggests that the ER is luminally continuous (Park et al., 2000), allowing free movement of proteins as large as GFP (Dayel et al., 1999), we considered whether the Golgi apparatus might provide the independent Ca^{2+} store recruited by PTH. The latter would be consistent with evidence that the Ca^{2+} release evoked by CCh or CCh with PTH is abolished by pretreatment with thapsigargin (Short and Taylor, 2000).

We used a low-affinity, red Ca^{2+} sensor (LAR-GECO1, K_D = 24 μM) (Wu et al., 2014) targeted to the lumen of either the ER or the medial/*trans*-Golgi apparatus (Figure 6A) to measure the free $[Ca^{2+}]$ within these organelles. These sensors were used with fluo-8 to report the changes in luminal and cytosolic $[Ca^{2+}]$ evoked by CCh and then PTH (Figure 6B). CCh and the subsequent addition of PTH evoked increases in $[Ca^{2+}]_i$ (Figure 6C), and they both caused decreases in the fluorescence of the ER and Golgi sensors (Figure 6D). Comparison of the effects of CCh and the subsequent addition of PTH on the ER and Golgi sensors ($\Delta F_{CCh}/\Delta F_{CCh\ then\ PTH}$) shows that neither organelle responded selectively to PTH (Figure 6E). The results suggest that the independence of the stores from which CCh or CCh with PTH release Ca^{2+} is not due to selective release of Ca^{2+} from the medial/*trans*-Golgi apparatus. We have not

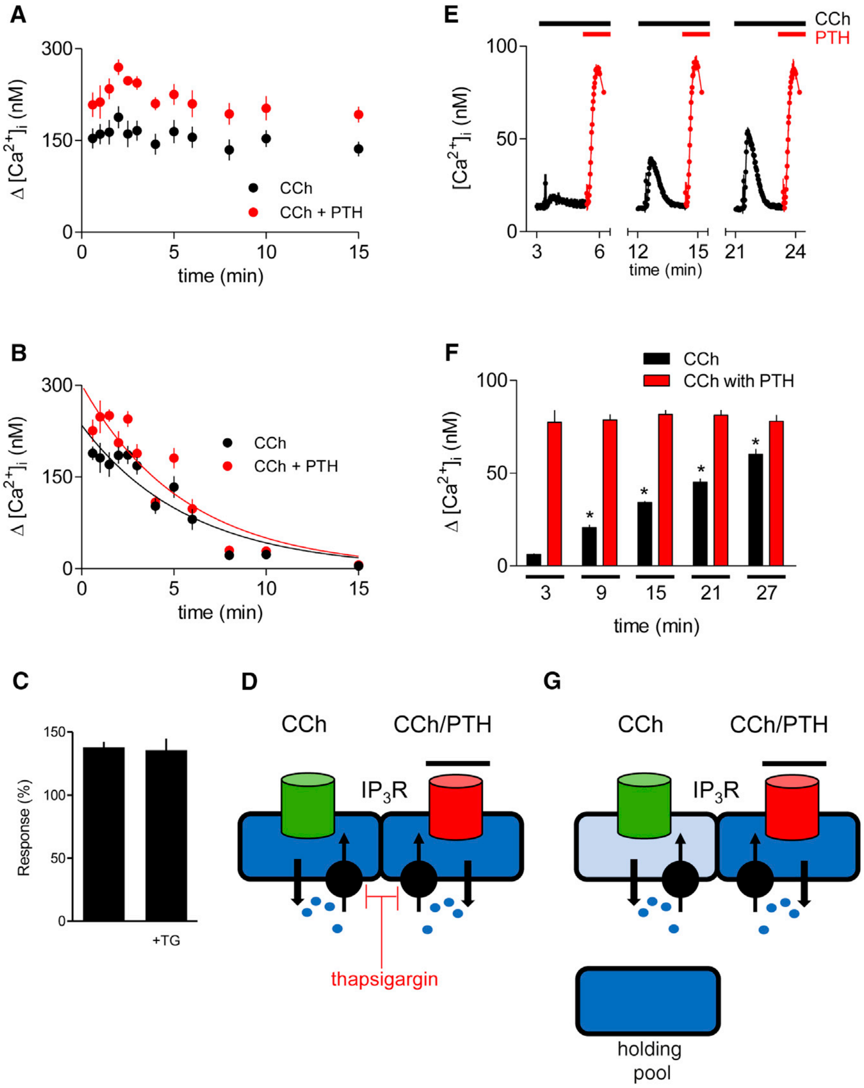

Figure 7. Independent Refilling of Dynamic Ca^{2+} Pools that Respond to CCh or CCh with PTH

(A) HEK-PR1 cells were incubated in Ca^{2+}-free HBS for the indicated times before addition of CCh (1 mM) alone or with PTH (100 nM). Results show peak increases in $[Ca^{2+}]_i$ ($\Delta[Ca^{2+}]_i$).

(B) Similar experiments in the presence of thapsigargin (1 μM) to inhibit SERCA. The lines are mono-exponential curve fits. Results show mean ± SEM, n = 3.

(C) The increase in $[Ca^{2+}]_i$ evoked by CCh with PTH in Ca^{2+}-free HBS alone or with thapsigargin (TG, 1 μM, 1 min) is shown relative to the time-matched response evoked by CCh alone (%, mean ± SEM, n = 3). Data are from (A) and (B).

(D) Two distinct Ca^{2+} stores, responding to CCh alone or CCh with PTH, maintain their integrity despite rapidly recycling their Ca^{2+} through the cytosol.

(E) HEK-PR1 cells were incubated with 30 μM CCh for 60 min in Ca^{2+}-free HBS to deplete the CCh-sensitive Ca^{2+} stores and then washed twice with Ca^{2+}-free HBS to remove CCh. Typical traces (mean ± SD from three wells) show the response to CCh (1 mM) and then PTH (100 nM) after the indicated periods of recovery in Ca^{2+}-free HBS.

(F) Summary results (mean ± SEM from three independent experiments) show the response to CCh alone and the subsequent response to PTH after the indicated periods of recovery. *$p < 0.05$, one-way ANOVA and Tukey's post hoc test, relative to measurement at 3 min.

(G) The stores that respond to CCh alone can refill in the absence of extracellular Ca^{2+} without affecting the Ca^{2+} content of the stores that respond to CCh with PTH. The "holding pool" from which these stores acquire Ca^{2+} has not been identified, although it may reside within mitochondria or lysosomes.

See also Figure S4.

examined the *cis*-Golgi, which has been reported to have a higher luminal Ca^{2+} concentration and more IP_3Rs than the medial/*trans*-Golgi (Pizzo et al., 2011).

CCh and CCh with PTH Release Ca^{2+} from Intracellular Stores that Dynamically Exchange Ca^{2+} with the Cytosol

We next considered whether the independence of the Ca^{2+} stores released by CCh alone or CCh with PTH might reflect the existence of a store that only very slowly exchanges Ca^{2+} with the cytosol. In Ca^{2+}-free HBS, the intracellular stores of unstimulated HEK-PR1 cells lose Ca^{2+} extremely slowly (Figure 3C), suggesting either that the stores exchange Ca^{2+} very slowly with the cytosol or that cells efficiently retain Ca^{2+} to allow rapid recycling to intracellular stores. Inhibition of SERCA with thapsigargin reveals that there is rapid cycling of Ca^{2+} across ER membranes. In Ca^{2+}-free HBS without thapsigargin, there was no significant loss of the response to CCh or CCh with PTH after 15 min (Figure 7A), whereas with thapsigargin there was no response to either stimulus after 15 min (Figure 7B). The rate of decline of the response was indistinguishable for Ca^{2+} signals evoked by CCh alone ($t_{1/2} = 4.0 \pm 0.2$ min, n = 3) or CCh with PTH ($t_{1/2} = 3.9 \pm 0.2$ min) (Figure 7B), suggesting that the Ca^{2+} stores released by CCh or CCh with PTH have similar basal rates of Ca^{2+} leak. Because PTH evokes Ca^{2+} release from a thapsigargin-sensitive Ca^{2+} store (Short and Taylor, 2000), these results confirm that responses to PTH are not dependent on Ca^{2+} being chased from one pool to another (Figure 7C), and they demonstrate that segregation of the two intracellular stores is maintained despite rapid cycling of Ca^{2+} between the cytosol and stores (Figure 7D).

The Two Ca^{2+} Stores Refill Independently

HEK-PR1 cells in Ca^{2+}-free HBS were incubated for 60 min with 30 μM CCh to empty the CCh-sensitive Ca^{2+} stores; the cells were then rapidly washed in Ca^{2+}-free HBS to remove CCh, and the responses to CCh alone or CCh with PTH in Ca^{2+}-free HBS were assessed during this recovery period. At the end of the 60 min incubation with 30 μM CCh, the response to addition of 1 mM CCh had declined to 1% ± 1% (n = 3) of the initial

response, consistent with previous results (Figures 3 and 4). Because there is no desensitization of M_3Rs with this stimulus regime (Figures 4 and S1), the results confirm that the CCh-sensitive stores were empty at the end of the sustained incubation. During the subsequent recovery period in Ca^{2+}-free HBS, the response to 1 mM CCh recovered relatively slowly (to ~10 times the initial response after 27 min). However, the response to addition of PTH after CCh remained constant over the entire recovery period (Figures 7E and 7F). Hence, under conditions where the CCh-sensitive store substantially refilled, there was no effect on the Ca^{2+} content of the store released by CCh with PTH. We have not determined the source of the intracellular Ca^{2+} that replenished the CCh-sensitive store, although mitochondria (Rizzuto et al., 2012) or lysosomes (López Sanjurjo et al., 2014) are likely candidates. Others have also reported refilling of IP_3-sensitive Ca^{2+} stores within the ER from unidentified intracellular sources (Suzuki et al., 2014). Our results, where CCh-sensitive stores refill without affecting the response to PTH, mirror those in Figure 4, where depletion of the CCh-sensitive stores had no impact on the subsequent response to PTH. Both sets of results establish the functional independence of the Ca^{2+} stores released by CCh alone and by CCh with PTH (Figure 7G).

Conclusions

Substantial evidence suggests that the ER is luminally continuous, and so unlikely to provide a barrier to free movement of Ca^{2+} within the ER lumen (Dayel et al., 1999; Mogami et al., 1997; Park et al., 2000; Rizzuto and Pozzan, 2006), but other evidence suggests some functional compartmentalization of ER Ca^{2+} stores. In HEK293 cells, for example, CCh and ATP, via their respective PLC-coupled receptors, can release Ca^{2+} from different IP_3-sensitive Ca^{2+} stores (Short et al., 2000). Further evidence for compartmentalization within ER Ca^{2+} stores includes measurements of sustained focal changes of luminal Ca^{2+} concentration within the ER and different responses of adjacent compartments to activation of IP_3R and ryanodine receptors (Golovina and Blaustein, 1997). ER Ca^{2+} pools that differ in their susceptibilities to SERCA inhibitors further suggest a degree of compartmentalization (Aulestia et al., 2011). A recent cryo-electron tomographic analysis of ER-plasma membrane contact sites, where the lumen of some ER is very constricted, suggests a possible structural basis for compartmentalization of ER Ca^{2+} stores (Fernández-Busnadiego et al., 2015).

Our present results demonstrate a remarkable functional independence of two discrete ER Ca^{2+} stores that persists despite each rapidly exchanging Ca^{2+} with the cytosol. The first store expresses IP_3Rs with modest affinity and responds to the IP_3 produced in response to CCh alone. The second store expresses IP_3Rs with greater affinity for IP_3 (possibly IP_3R2), but these IP_3Rs are unmasked only in the presence of cAMP. We have not established the identities of the independent Ca^{2+} stores, although it is clear that IP_3R2, which we showed to be important for responses to PTH (Tovey et al., 2008), has a different subcellular distribution to that of IP_3R1 and IP_3R3 (Figure S4). The interactions between PTH and CCh in HEK-PR1 cells are reminiscent of those between PTH and ATP in osteoblasts (Buckley et al., 2001), suggesting that the mechanisms we have described here may be widespread. We conclude that a strict functional compartmentalization of ER Ca^{2+} stores allows IP_3 alone and IP_3 with cAMP to release Ca^{2+} from discrete stores. Our results suggest a hitherto unexpected versatility in IP_3-evoked Ca^{2+} release from the ER.

EXPERIMENTAL PROCEDURES

Measurements of $[Ca^{2+}]_i$ and Intracellular cAMP

HEK-PR1 cells (Short and Taylor, 2000) were cultured as described previously (Tovey et al., 2008). HEK293 cells (without PTH receptors) were used for some experiments because ATP evoked larger Ca^{2+} signals in these cells than in HEK-PR1 cells. Measurements of intracellular free Ca^{2+} concentration ($[Ca^{2+}]_i$) in single cells and populations of fluo-4-loaded HEK-PR1 cells were performed as previously described (Tovey et al., 2008). Intracellular cAMP was measured as previously described (Pantazaka et al., 2013) (Supplemental Experimental Procedures).

Measurements of Luminal-Free $[Ca^{2+}]$ within the ER and Golgi Apparatus

A low-affinity (K_D = 24 μM), red genetically encoded Ca^{2+} sensor (LAR-GECO1) was used to record the luminal $[Ca^{2+}]$ within the ER ($[Ca^{2+}]_{ER}$ using ER-LAR-GECO1) (Wu et al., 2014) or within the Golgi apparatus ($[Ca^{2+}]_{GA}$ using Golgi-LAR-GECO1). Details are given in the Supplemental Experimental Procedures.

Expression of IRBIT and siRNA-Mediated Knockdown

BacMam viruses were used to express IRBIT and IRBIT-S68A in HEK-PR1 cells. Cells were transfected with siRNAs to reduce IRBIT expression in HEK-PR1 cells (Supplemental Experimental Procedures).

Statistical Analyses

The experiments reported were completed over a prolonged period during which there was some variation between absolute values for changes in $[Ca^{2+}]_i$ and sensitivities to CCh and PTH. Hence, all statistical comparisons use observations from matched analyses. For each experiment, the concentration-effect relationship was fitted to a logistic equation (GraphPad Prism version 5). From each experiment, pEC_{50} (−log of the half-maximally effective concentration $[EC_{50}]$ in M) and the maximal response were obtained and used for statistical analyses. Most graphs show mean results from several experiments, but values (pEC_{50}, etc.) were computed from individual experiments before pooling for statistical comparisons.

SUPPLEMENTAL INFORMATION

Supplemental Information includes Supplemental Experimental Procedures and four figures and can be found with this article online at http://dx.doi.org/10.1016/j.celrep.2016.12.058.

AUTHOR CONTRIBUTIONS

V.K. performed experiments. S.C.T. contributed to fluorescence experiments. S.M. performed western blot (WB) and analyses of STIM1. D.L.P. contributed to design and analysis of targeted Ca^{2+} indicators. C.W.T. supervised the project and contributed to data analysis. C.W.T with V.K and D.L.P. wrote the paper. All authors contributed to review of the paper.

ACKNOWLEDGMENTS

This work was supported by the Wellcome Trust (grant 101844) and the Biotechnology and Biological Sciences Research Council (BBSRC) (grant L000075). V.K. was supported by the German Academic Exchange Service. V.K. and S.M. were supported by studentships from the BBSRC. S.C.T. is now an employee of Cairn Research.

Received: September 9, 2016
Revised: November 17, 2016
Accepted: December 19, 2016
Published: January 17, 2017

REFERENCES

Alswied, A., and Parekh, A.B. (2015). Ca^{2+} influx through store-operated calcium channels replenishes the functional phosphatidylinositol 4,5-bisphosphate pool used by cysteinyl leukotriene type I receptors. J. Biol. Chem. *290*, 29555–29566.

Ando, H., Mizutani, A., Matsu-ura, T., and Mikoshiba, K. (2003). IRBIT, a novel inositol 1,4,5-trisphosphate (IP_3) receptor-binding protein, is released from the IP_3 receptor upon IP_3 binding to the receptor. J. Biol. Chem. *278*, 10602–10612.

Ando, H., Mizutani, A., Kiefer, H., Tsuzurugi, D., Michikawa, T., and Mikoshiba, K. (2006). IRBIT suppresses IP_3 receptor activity by competing with IP_3 for the common binding site on the IP_3 receptor. Mol. Cell *22*, 795–806.

Aulestia, F.J., Redondo, P.C., Rodríguez-García, A., Rosado, J.A., Salido, G.M., Alonso, M.T., and García-Sancho, J. (2011). Two distinct calcium pools in the endoplasmic reticulum of HEK-293T cells. Biochem. J. *435*, 227–235.

Aulestia, F.J., Alonso, M.T., and García-Sancho, J. (2015). Differential calcium handling by the cis and trans regions of the Golgi apparatus. Biochem. J. *466*, 455–465.

Betzenhauser, M.J., and Yule, D.I. (2010). Regulation of inositol 1,4,5-trisphosphate receptors by phosphorylation and adenine nucleotides. Curr. Top. Membr. *66*, 273–298.

Bird, G.S., Hwang, S.Y., Smyth, J.T., Fukushima, M., Boyles, R.R., and Putney, J.W., Jr. (2009). STIM1 is a calcium sensor specialized for digital signaling. Curr. Biol. *19*, 1724–1729.

Buckley, K.A., Wagstaff, S.C., McKay, G., Gaw, A., Hipskind, R.A., Bilbe, G., Gallagher, J.A., and Bowler, W.B. (2001). Parathyroid hormone potentiates nucleotide-induced $[Ca^{2+}]_i$ release in rat osteoblasts independently of G_q activation or cyclic monophosphate accumulation. A mechanism for localizing systemic responses in bone. J. Biol. Chem. *276*, 9565–9571.

Dayel, M.J., Hom, E.F., and Verkman, A.S. (1999). Diffusion of green fluorescent protein in the aqueous-phase lumen of endoplasmic reticulum. Biophys. J. *76*, 2843–2851.

Delmas, P., Wanaverbecq, N., Abogadie, F.C., Mistry, M., and Brown, D.A. (2002). Signaling microdomains define the specificity of receptor-mediated InsP(3) pathways in neurons. Neuron *34*, 209–220.

Devogelaere, B., Nadif Kasri, N., Derua, R., Waelkens, E., Callewaert, G., Missiaen, L., Parys, J.B., and De Smedt, H. (2006). Binding of IRBIT to the IP_3 receptor: determinants and functional effects. Biochem. Biophys. Res. Commun. *343*, 49–56.

Devogelaere, B., Beullens, M., Sammels, E., Derua, R., Waelkens, E., van Lint, J., Parys, J.B., Missiaen, L., Bollen, M., and De Smedt, H. (2007). Protein phosphatase-1 is a novel regulator of the interaction between IRBIT and the inositol 1,4,5-trisphosphate receptor. Biochem. J. *407*, 303–311.

Dode, L., Andersen, J.P., Vanoevelen, J., Raeymaekers, L., Missiaen, L., Vilsen, B., and Wuytack, F. (2006). Dissection of the functional differences between human secretory pathway Ca^{2+}/Mn^{2+}-ATPase (SPCA) 1 and 2 isoenzymes by steady-state and transient kinetic analyses. J. Biol. Chem. *281*, 3182–3189.

Fernández-Busnadiego, R., Saheki, Y., and De Camilli, P. (2015). Three-dimensional architecture of extended synaptotagmin-mediated endoplasmic reticulum-plasma membrane contact sites. Proc. Natl. Acad. Sci. USA *112*, E2004–E2013.

Fink, C.C., Slepchenko, B., and Loew, L.M. (1999). Determination of time-dependent inositol-1,4,5-trisphosphate concentrations during calcium release in a smooth muscle cell. Biophys. J. *77*, 617–628.

Foskett, J.K., White, C., Cheung, K.H., and Mak, D.O. (2007). Inositol trisphosphate receptor Ca^{2+} release channels. Physiol. Rev. *87*, 593–658.

Golovina, V.A., and Blaustein, M.P. (1997). Spatially and functionally distinct Ca^{2+} stores in sarcoplasmic and endoplasmic reticulum. Science *275*, 1643–1648.

He, Q., Zhu, Y., Corbin, B.A., Plagge, A., and Bastepe, M. (2015). The G protein α subunit variant $XL\alpha_s$ promotes inositol 1,4,5-trisphosphate signaling and mediates the renal actions of parathyroid hormone in vivo. Sci. Signal. *8*, ra84.

Iwai, M., Tateishi, Y., Hattori, M., Mizutani, A., Nakamura, T., Futatsugi, A., Inoue, T., Furuichi, T., Michikawa, T., and Mikoshiba, K. (2005). Molecular cloning of mouse type 2 and type 3 inositol 1,4,5-trisphosphate receptors and identification of a novel type 2 receptor splice variant. J. Biol. Chem. *280*, 10305–10317.

Kiefer, H., Mizutani, A., Iemura, S., Natsume, T., Ando, H., Kuroda, Y., and Mikoshiba, K. (2009). Inositol 1,4,5-triphosphate receptor-binding protein released with inositol 1,4,5-triphosphate (IRBIT) associates with components of the mRNA 3' processing machinery in a phosphorylation-dependent manner and inhibits polyadenylation. J. Biol. Chem. *284*, 10694–10705.

Kloor, D., Hermes, M., Kirschler, J., Müller, M., Hagen, N., Kalbacher, H., Stevanovic, S., and Osswald, H. (2009). Determinants for the cAMP-binding site at the S-adenosylhomocysteine-hydrolase. Naunyn Schmiedebergs Arch. Pharmacol. *380*, 215–222.

Konieczny, V. (2015). Signalling from extracellular stimuli to inositol 1,4,5-trisphosphate receptors. PhD thesis (University of Cambridge).

Konieczny, V., Keebler, M.V., and Taylor, C.W. (2012). Spatial organization of intracellular Ca^{2+} signals. Semin. Cell Dev. Biol. *23*, 172–180.

Kurian, N., Hall, C.J., Wilkinson, G.F., Sullivan, M., Tobin, A.B., and Willars, G.B. (2009). Full and partial agonists of muscarinic M3 receptors reveal single and oscillatory Ca2+ responses by beta 2-adrenoceptors. J. Pharmacol. Exp. Ther. *330*, 502–512.

Lewis, R.S. (2011). Store-operated calcium channels: new perspectives on mechanism and function. Cold Spring Harb. Perspect. Biol. *3*, a003970.

López Sanjurjo, C.I., Tovey, S.C., and Taylor, C.W. (2014). Rapid recycling of Ca^{2+} between IP_3-sensitive stores and lysosomes. PLoS ONE *9*, e111275.

Luik, R.M., Wang, B., Prakriya, M., Wu, M.M., and Lewis, R.S. (2008). Oligomerization of STIM1 couples ER calcium depletion to CRAC channel activation. Nature *454*, 538–542.

Masuda, W., Betzenhauser, M.J., and Yule, D.I. (2010). $InsP_3R$-associated cGMP kinase substrate determines inositol 1,4,5-trisphosphate receptor susceptibility to phosphoregulation by cyclic nucleotide-dependent kinases. J. Biol. Chem. *285*, 37927–37938.

Matsu-ura, T., Michikawa, T., Inoue, T., Miyawaki, A., Yoshida, M., and Mikoshiba, K. (2006). Cytosolic inositol 1,4,5-trisphosphate dynamics during intracellular calcium oscillations in living cells. J. Cell Biol. *173*, 755–765.

Meena, A., Tovey, S.C., and Taylor, C.W. (2015). Sustained signalling by PTH modulates IP3 accumulation and IP3 receptors through cyclic AMP junctions. J. Cell Sci. *128*, 408–420.

Mogami, H., Nakano, K., Tepikin, A.V., and Petersen, O.H. (1997). Ca^{2+} flow via tunnels in polarized cells: recharging of apical Ca^{2+} stores by focal Ca^{2+} entry through basal membrane patch. Cell *88*, 49–55.

Pantazaka, E., Taylor, E.J.A., Bernard, W.G., and Taylor, C.W. (2013). Ca(2+) signals evoked by histamine H1 receptors are attenuated by activation of prostaglandin EP2 and EP4 receptors in human aortic smooth muscle cells. Br. J. Pharmacol. *169*, 1624–1634.

Park, M.K., Petersen, O.H., and Tepikin, A.V. (2000). The endoplasmic reticulum as one continuous Ca(2+) pool: visualization of rapid Ca(2+) movements and equilibration. EMBO J. *19*, 5729–5739.

Park, S., Shcheynikov, N., Hong, J.H., Zheng, C., Suh, S.H., Kawaai, K., Ando, H., Mizutani, A., Abe, T., Kiyonari, H., et al. (2013). Irbit mediates synergy between ca(2+) and cAMP signaling pathways during epithelial transport in mice. Gastroenterology *145*, 232–241.

Pizzo, P., Lissandron, V., Capitanio, P., and Pozzan, T. (2011). Ca(2+) signalling in the Golgi apparatus. Cell Calcium *50*, 184–192.

Prole, D.L., and Taylor, C.W. (2016). Inositol 1,4,5-trisphosphate receptors and their protein partners as signalling hubs. J. Physiol. *594*, 2849–2866.

Rizzuto, R., and Pozzan, T. (2006). Microdomains of intracellular Ca^{2+}: molecular determinants and functional consequences. Physiol. Rev. *86*, 369–408.

Rizzuto, R., De Stefani, D., Raffaello, A., and Mammucari, C. (2012). Mitochondria as sensors and regulators of calcium signalling. Nat. Rev. Mol. Cell Biol. *13*, 566–578.

Rodríguez-Prados, M., Rojo-Ruiz, J., Aulestia, F.J., García-Sancho, J., and Alonso, M.T. (2015). A new low-Ca^{2+} affinity GAP indicator to monitor high Ca^{2+} in organelles by luminescence. Cell Calcium *58*, 558–564.

Short, A.D., and Taylor, C.W. (2000). Parathyroid hormone controls the size of the intracellular Ca(2+) stores available to receptors linked to inositol trisphosphate formation. J. Biol. Chem. *275*, 1807–1813.

Short, A.D., Winston, G.P., and Taylor, C.W. (2000). Different receptors use inositol trisphosphate to mobilize Ca(2+) from different intracellular pools. Biochem. J. *351*, 683–686.

Suzuki, J., Kanemaru, K., Ishii, K., Ohkura, M., Okubo, Y., and Iino, M. (2014). Imaging intraorganellar Ca^{2+} at subcellular resolution using CEPIA. Nat. Commun. *5*, 4153.

Taylor, C.W., and Tovey, S.C. (2012). From parathyroid hormone to cytosolic Ca^{2+} signals. Biochem. Soc. Trans. *40*, 147–152.

Tovey, S.C., and Taylor, C.W. (2013). Cyclic AMP directs inositol (1,4,5)-trisphosphate-evoked Ca^{2+} signalling to different intracellular Ca^{2+} stores. J. Cell Sci. *126*, 2305–2313.

Tovey, S.C., Goraya, T.A., and Taylor, C.W. (2003). Parathyroid hormone increases the sensitivity of inositol trisphosphate receptors by a mechanism that is independent of cyclic AMP. Br. J. Pharmacol. *138*, 81–90.

Tovey, S.C., Dedos, S.G., Taylor, E.J.A., Church, J.E., and Taylor, C.W. (2008). Selective coupling of type 6 adenylyl cyclase with type 2 IP3 receptors mediates direct sensitization of IP3 receptors by cAMP. J. Cell Biol. *183*, 297–311.

Tovey, S.C., Dedos, S.G., Rahman, T., Taylor, E.J.A., Pantazaka, E., and Taylor, C.W. (2010). Regulation of inositol 1,4,5-trisphosphate receptors by cAMP independent of cAMP-dependent protein kinase. J. Biol. Chem. *285*, 12979–12989.

Tu, J.C., Xiao, B., Yuan, J.P., Lanahan, A.A., Leoffert, K., Li, M., Linden, D.J., and Worley, P.F. (1998). Homer binds a novel proline-rich motif and links group 1 metabotropic glutamate receptors with IP_3 receptors. Neuron *21*, 717–726.

Wang, S.S., Alousi, A.A., and Thompson, S.H. (1995). The lifetime of inositol 1,4,5-trisphosphate in single cells. J. Gen. Physiol. *105*, 149–171.

Willoughby, D., and Cooper, D.M.F. (2007). Organization and Ca^{2+} regulation of adenylyl cyclases in cAMP microdomains. Physiol. Rev. *87*, 965–1010.

Wong, A.K., Capitanio, P., Lissandron, V., Bortolozzi, M., Pozzan, T., and Pizzo, P. (2013). Heterogeneity of Ca^{2+} handling among and within Golgi compartments. J. Mol. Cell Biol. *5*, 266–276.

Wu, J., Prole, D.L., Shen, Y., Lin, Z., Gnanasekaran, A., Liu, Y., Chen, L., Zhou, H., Chen, S.R., Usachev, Y.M., et al. (2014). Red fluorescent genetically encoded Ca^{2+} indicators for use in mitochondria and endoplasmic reticulum. Biochem. J. *464*, 13–22.

Yang, Z., Kirton, H.M., MacDougall, D.A., Boyle, J.P., Deuchars, J., Frater, B., Ponnambalam, S., Hardy, M.E., White, E., Calaghan, S.C., et al. (2015). The Golgi apparatus is a functionally distinct Ca^{2+} store regulated by the PKA and Epac branches of the β_1-adrenergic signaling pathway. Sci. Signal. *8*, ra101.

Zaika, O., Zhang, J., and Shapiro, M.S. (2011). Combined phosphoinositide and Ca^{2+} signals mediating receptor specificity toward neuronal Ca^{2+} channels. J. Biol. Chem. *286*, 830–841.

Cell Reports, Volume *18*

Supplemental Information

Cyclic AMP Recruits a Discrete Intracellular Ca^{2+} Store by Unmasking Hypersensitive IP_3 Receptors

Vera Konieczny, Stephen C. Tovey, Stefania Mataragka, David L. Prole, and Colin W. Taylor

Abbreviations

BAPTA, 1,2-bis(*O*-aminophenoxy)ethane-N,N,N',N'-tetraacetic acid; $[Ca^{2+}]_i$ intracellular free Ca^{2+} concentration; $[Ca^{2+}]_{ER}$, $[Ca^{2+}]_{GA}$, free Ca^{2+} concentration within the ER, Golgi apparatus; cAMP, 3′,5′-cyclic adenosine monophosphate; CCh, carbachol (carbamylcholine); 8-Br-cAMP, 8-bromo cAMP; EC_{50}, half-maximally effective concentration; EGFP, enhanced green fluorescent protein; HBS, HEPES-buffered saline; HEK-PR1, human embryonic kidney cell stably expressing human type 1 PTH receptor; IP_3 inositol 1,4,5-trisphosphate; IP_3R, IP_3 receptor; IRBIT, IP_3R-binding protein released by IP_3; Mfm, 5-methylfurmethiodide; MOI, multiplicity of infection; M_r, relative molecular mass; pEC_{50}, -log EC_{50}; PCR, polymerase chain reaction; PFU, plaque-forming unit; PKA, cyclic AMP-dependent protein kinase; PTH, parathyroid hormone (residues 1-34); SD, standard deviation; SEM, standard error of the mean; SERCA, sarcoplasmic/endoplasmic reticulum Ca^{2+}-ATPase; siRNA, small interfering RNA; SOCE, store-operated Ca^{2+} entry; SPCA, secretory pathway Ca^{2+}-ATPase; $t_{½}$, half-time; STIM1, stromal interaction molecule 1; TIRFM, total internal refection fluorescence microscopy; WB, Western blot.

SUPPLEMENTAL EXPERIMENTAL PROCEDURES

Materials

Cell culture materials and fluo-4 acetoxymethyl ester (fluo-4/AM) were from Life Technologies (Paisley, UK). Fluo-8/AM was from AAT Bioquest (Sunnyvale, CA, USA). Poly-L-lysine, Triton X-100, carbamylcholine hydrochloride (carbachol, CCh), methylatropine (Atr), isoprenaline, Pluronic F-127, trichloroacetic acid, alumina, Dowex 50WX4-400 and dimethyl sulfoxide (DMSO) were from Sigma-Aldrich (Poole, Dorset, UK). Human parathyroid hormone (residues 1-34, PTH) was from Bachem (St Helens, UK). Thapsigargin was from Tocris (Bristol, UK). 1,2-bis(*O*-aminophenoxy)ethane-N,N,N',N'-tetraacetic acid (BAPTA) was from Molekula (Dorset, UK). Ionomycin was from Apollo Scientific (Bredbury, UK). Imidazole was from Thermo Scientific (Waltham, MA, USA). ^{3}H-adenine (18.4 Ci/mmol) and Ultima Gold scintillant were from Perkin Elmer (Waltham, MA, USA).

Measurements of $[Ca^{2+}]_i$

HEK293 cells stably expressing human type 1 PTH receptor (HEK-PR1 cells) (Short and Taylor, 2000) were cultured as described previously (Tovey et al., 2008). HEK293 cells (without PTH receptors) were used for some experiments because ATP evoked larger Ca^{2+} signals in these cells than in HEK-PR1 cells.

For measurements of intracellular free Ca^{2+} concentration ($[Ca^{2+}]_i$) in cell populations, HEK-PR1 cells (8 x 10^4 cells/well in poly-L-lysine-coated black full-area 96-well plates, Greiner Bio-One) were grown to confluence (48 h). The cells were washed in HEPES-buffered saline (HBS) and loaded with fluo-4 by incubation with fluo-4/AM (2 µM) in HBS with Pluronic F-127 (0.02 %, v/v) for 1 h at 20 °C in the dark. Cells were then washed and incubated with HBS for 45 min to allow de-esterification of the fluo-4/AM. HBS had the following composition (in mM): NaCl (135), KCl (5.9), $MgCl_2$ (1.2), $CaCl_2$ (1.5), HEPES (11.6), D-glucose (11.5), pH 7.3. Fluorescence (excitation, 485 nm; emission, 525 nm) was recorded at 1.44-s intervals in HBS (100-160 µl) at 20 °C using a FlexStation III fluorescence plate-reader (MDS Analytical Devices, Wokingham, UK) (Tovey et al., 2008). Minimum (F_{min}) and maximum (F_{max}) fluorescence values were determined from parallel wells on each plate by addition of 0.1 % Triton X-100 with 10 mM BAPTA (F_{min}) or 10 mM $CaCl_2$ (F_{max}). Fluorescence values (F) were calibrated to $[Ca^{2+}]_i$ from:

$$[Ca^{2+}]_i = K_D \times \frac{F - F_{min}}{F_{max} - F}$$

The K_D of fluo-4 was assumed to be 345 nM (Gee et al., 2000). Fluo-8 was used for some experiments (K_D = 389 nM).

For experiments in Ca^{2+}-free HBS, cells were incubated in normal HBS (60 µl) to maintain the Ca^{2+} content of the intracellular stores before addition of Ca^{2+}-free HBS containing 5 mM BAPTA (60 µl) 20 s before stimulation (final BAPTA and Ca^{2+} concentrations of 2.5 mM and 0.75 mM, respectively; free $[Ca^{2+}]$ < 60 nM). Stimuli (20 µl) were added by an automated pipetting system from stock solutions prepared in HBS or Ca^{2+}-free HBS, as appropriate.

For single-cell measurements of $[Ca^{2+}]_i$, cells were seeded onto poly-L-lysine-coated 35-mm glass-bottomed dishes (MatTek, number 1 coverglass) and grown to ~80 % confluence (48 h). Cells were loaded with fluo-4 and fluorescence was recorded at 20°C using an Olympus IX81 inverted microscope with a 40x/1.35 NA objective. Cells were illuminated every 1 s with a mercury xenon lamp and U-MNIBA filter set (Olympus, excitation 470-495 nm, emission 510-550 nm). Fluorescence was detected using an Andor iXon 897 EMCCD camera. Fluorescence values were calibrated to $[Ca^{2+}]_i$ as described above. Images were processed using CellR software (Olympus).

Measurements of Intracellular cAMP

HEK-PR1 cells (4.5 x 10^5 cells/well) grown to confluence in 24-well plates (48 h) were incubated for 2 h in normal growth medium with ^{3}H-adenine (1 µCi/well) at 37 °C with 5 %

CO_2. The cells were washed twice with HBS and then incubated in Ca^{2+}-free HBS with appropriate stimuli at 20 °C. After 5 min, the medium was removed, and the reaction was terminated by addition of ice-cold trichloroacetic acid (5 %). ^{3}H-cAMP was separated from other ^{3}H-adenine nucleotides by sequential column chromatography using a Dowex cation exchange resin and alumina as described (Pantazaka et al., 2013). The activity of the eluates was determined by liquid scintillation counting in Ultima Gold scintillant. ^{3}H-cAMP levels are expressed as percentages of the sum of ^{3}H-ATP, ^{3}H-ADP and ^{3}H-cAMP activities.

Measurements of Luminal Free [Ca^{2+}] Within the ER and Golgi Apparatus

A low-affinity (K_D = 24 μM) red genetically encoded Ca^{2+} sensor (LAR-GECO1) was used to record the luminal [Ca^{2+}] within the ER ([Ca^{2+}]$_{ER}$ using ER-LAR-GECO1) (Wu et al., 2014) or within the Golgi apparatus ([Ca^{2+}]$_{GA}$ using Golgi-LAR-GECO1). Targeting to the medial/trans Golgi apparatus (Llopis et al., 1998) was achieved by fusing the N-terminus of LAR-GECO1 to a 61-residue N-terminal sequence from human β-1,4-galactosidase 1 (MRLREPLLSGSAAMPGASLQRACRLLVAVCALHLGVTLVYYLAGRDLSRLPQLVG VSTPLQ) (Tian et al., 2014). The targeting signal and flanking restriction sites (BamHI and EcoRI) were added to LAR-GECO1 by PCR using CMV-ER-LAR-GECO1 (Addgene plasmid 61244) (Wu et al., 2014) as a template. Digestion with BamHI and EcoRI then allowed subcloning into similarly digested pcDNA3.1(+) (Thermo Fisher) to give Golgi-LAR-GECO1.

HEK-PR1 cells were seeded on fibronectin-coated 35-mm glass-bottomed dishes (MatTek, number 1 coverglass) and transfected (1 μg DNA/dish) after 24 h using TransIT-LT1 (Mirus Bio, Madison, WI, USA). Cells were used 24 h after transfection. For simultaneous measurements of [Ca^{2+}]$_i$ and either [Ca^{2+}]$_{ER}$ or [Ca^{2+}]$_{GA}$, cells were loaded with fluo-8/AM as described for fluo-4/AM, and imaged using an Olympus IX83 inverted microscope with a 100x/1.49 NA objective. Cells were alternately illuminated (50-100 ms for each wavelength on a 400-ms cycle) with a 470-nm light-emitting diode (LED) (Spectra X, Lumencor) for visualizing fluo-8 fluorescence and a 561-laser (Coherent) for visualizing ER-LAR-GECO1 and Golgi-LAR-GECO1, via a 405/488/561/647-nm quad band dichroic/emitter (TRF89902, Chroma Technology). Fluorescence was detected using an Andor 6 iXon Ultra EMCCD camera. Images were processed using MetaMorph (Molecular Devices).

Expression of IRBIT and siRNA-Mediated Knockdown

The coding sequences of mouse IRBIT (sequence identifier: GI 17390492) and IRBIT with an N-terminal EGFP tag and linker sequence (SGRTQISSSSFEF were cloned into pENTR1A (Life Technologies) as SalI/NotI fragments. For the dominant-negative form of IRBIT (IRBIT-S68A) (Ando et al., 2006), the codon TCA encoding Ser68 was mutated to GCA (Ala) using the QuikChange Lightning Mutagenesis Kit (Agilent) following the manufacturer's instructions.

To generate BacMam viruses (Fornwald et al., 2007) for expression of IRBIT and IRBIT-S68A, the open reading frames of the IRBIT constructs were transferred from pENTR1A to the pCMV-DEST vector (Life Technologies). Viral stocks (P1-P3) of each construct were prepared following the manufacturer's instructions (Life Technologies). For transduction of HEK-PR1 cells, the P3 viral stock (10 µl, ~10^8 PFU/ml) was added to HEK-PR1 cells in suspension (10^5 cells in 200 µl of culture medium, MOI ~10 PFU/cell). Cells were used after 48 h. Western blots (WB) using a mouse polyclonal anti-IRBIT antiserum (1:750, #H00010768-A01, Abnova, Littleton, CO, USA) were used to quantify IRBIT expression.

For siRNA-mediated knockdown of IRBIT, HiPerFect transfection reagent (Qiagen, 0.75 µl/well or 12 µl/well for 96- and 6-well plates, respectively) was added to 40 nM siRNA in serum-free DMEM/F-12 GlutaMAX medium (50 µl or 200 µl for 96- and 6-well plates). The mixture was then incubated for 15 min at 22 °C to allow complex formation. Cells were seeded onto poly-L-lysine-coated wells in normal medium: 3 x 10^4 cells/well for 96-well plates used for measurements of [Ca^{2+}]$_i$, and 4.8 x 10^5 cells/well in 6-well plates for WB analysis. The siRNAs used were Hs_AHCYL1_1 and Hs_AHCYL1_2 (SI00090328, SI00090335) and the AllStars Negative Control siRNA (SI036550318) (Qiagen).

Translocation of mCherry-STIM1 to ER-Plasma Membrane Junctions
HEK-PR1 cells grown on poly-L-lysine-coated 35-mm glass-bottomed dishes (MatTek) were transfected with STIM1-mCherry (Wang et al., 2010) using TransIT-LT1 (1 μg plasmid DNA/dish). After 24 h, cells were loaded with the Ca^{2+} indicator, Cal-520, by incubation with Cal-520/AM (2 μM, Stratech Scientific Ltd) in HBS containing F-127 pluronic acid (0.02%). After 1 h, the cells were washed and incubated in HBS to allow de-esterification of the indicator (30 min). TIRFM, at 20°C in HBS, used an Olympus IX83 inverted microscope with 100x/1.49 NA TIRF objective. Diode-pumped solid-state lasers (iLas Laser System, Cairn) were used to excite the fluorescence of Cal-520 (488 nm, emission 525 nm) and m-Cherry (561 nm, emission 630 nm). Emitted light was captured by an Andor iXon 897 EMCCD camera at 1-s intervals for Cal-520 (50-ms capture interval) and (to minimize bleaching) for 20 ms from frames captured before or 2 min after addition of each stimulus for m-Cherry. Images were corrected for background by subtraction of fluorescence determined from an area outside the cell.

SUPPLEMENTAL REFERENCES

Ando, H., Mizutani, A., Kiefer, H., Tsuzurugi, D., Michikawa, T., and Mikoshiba, K. (2006). IRBIT suppresses IP_3 receptor activity by competing with IP_3 for the common binding site on the IP_3 receptor. Mol Cell *22*, 795-806.

Fornwald, J.A., Lu, Q., Wang, D., and Ames, R.S. (2007). Gene expression in mammalian cells using BacMam, a modified baculovirus system. Methods Mol Biol *388*, 95-114.

Gee, K.R., Brown, K.A., Chen, W.N., Bishop-Stewart, J., Gray, D., and Johnson, I. (2000). Chemical and physiological characterization of fluo-4 Ca^{2+}-indicator dyes. Cell Calcium *27*, 97-106.

Llopis, J., McCaffery, J.M., Miyawaki, A., Farquhar, M.G., and Tsien, R.Y. (1998). Measurement of cytosolic, mitochondrial, and Golgi pH in single living cells with green fluorescent proteins. Proc Natl Acad Sci USA *95*, 6803-6808.

Pantazaka, E., Taylor, E.J.A., Bernard, W., and Taylor, C.W. (2013). Ca^{2+} signals evoked by histamine H_1 receptors are attenuated by activation of prostaglandin EP_2 receptors in human aortic smooth muscle. Br J Pharmacol *169*, 1624-1634.

Short, A.D., and Taylor, C.W. (2000). Parathyroid hormone controls the size of the intracellular Ca^{2+} stores available to receptors linked to inositol trisphosphate formation. J Biol Chem *275*, 1807-1813.

Tian, G., Ropelewski, P., Nemet, I., Lee, R., Lodowski, K.H., and Imanishi, Y. (2014). An unconventional secretory pathway mediates the cilia targeting of peripherin/rds. J Neurosci *34*, 992-1006.

Tovey, S.C., Dedos, S.G., Taylor, E.J.A., Church, J.E., and Taylor, C.W. (2008). Selective coupling of type 6 adenylyl cyclase with type 2 IP_3 receptors mediates a direct sensitization of IP_3 receptors by cAMP. J Cell Biol *183*, 297-311.

Wang, Y., Deng, X., Mancarella, S., Hendron, E., Eguchi, S., Soboloff, J., Tang, X.D., and Gill, D.L. (2010). The calcium store sensor, STIM1, reciprocally controls Orai and $Ca_V1.2$ channels. Science *330*, 105-109.

Wu, J., Prole, D.L., Shen, Y., Lin, Z., Gnanasekaran, A., Liu, Y., Chen, L., Zhou, H., Chen, S.R., Usachev, Y.M., *et al.* (2014). Red fluorescent genetically encoded Ca^{2+} indicators for use in mitochondria and endoplasmic reticulum. Biochem J *464*, 13-22.

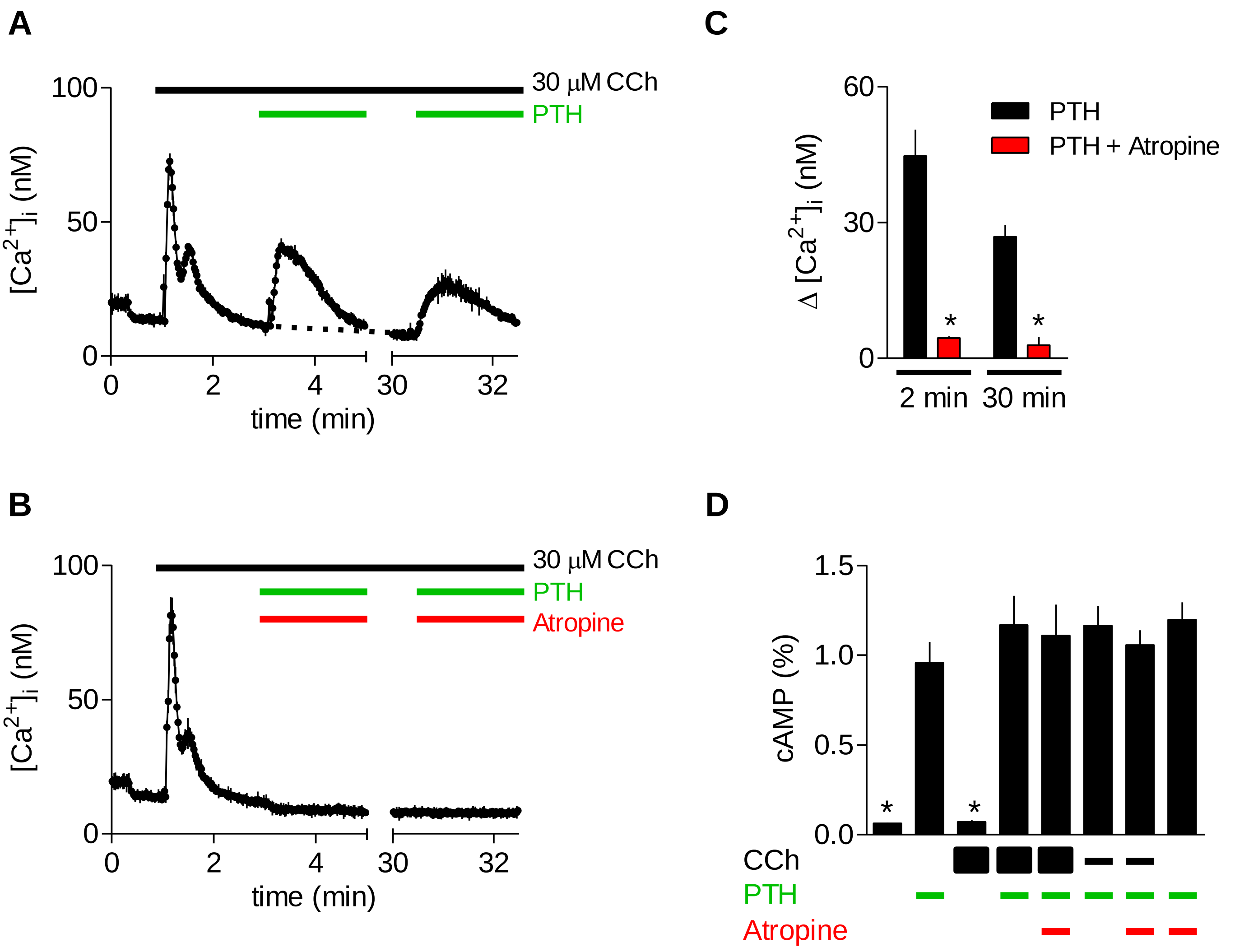

Figure S1. PTH-Evoked Ca^{2+} Release Requires Continuous Stimulation of M_3Rs. Related to Figure 4.

(A, B) HEK-PR1 cells were stimulated with 30 µM CCh in Ca^{2+}-free HBS before addition of PTH alone (100 nM) (A) or with methylatropine (10 µM) (B). Each panel shows results from 2 separate experiments in which PTH was added 2 or 30 min after addition of CCh. The typical traces show mean ± SD from 3 measurements.
(C) Summary shows the increase in $[Ca^{2+}]_i$ evoked by PTH (mean ± SEM from 3 independent experiments). $^{*}P < 0.05$, Student's *t*-test.
(D) Intracellular cAMP concentrations (% of ^{3}H-adenine nucleotides) in HEK-PR1 cells measured 5 min after addition of PTH (100 nM) with methylatropine (10 µM) and/or CCh (1 mM or 30 µM, thick and thin bar, respectively) as indicated. Results show means ± SEM, n = 3. $^{*}P < 0.05$, one-way ANOVA and Tukey's post hoc test, relative to all other conditions.

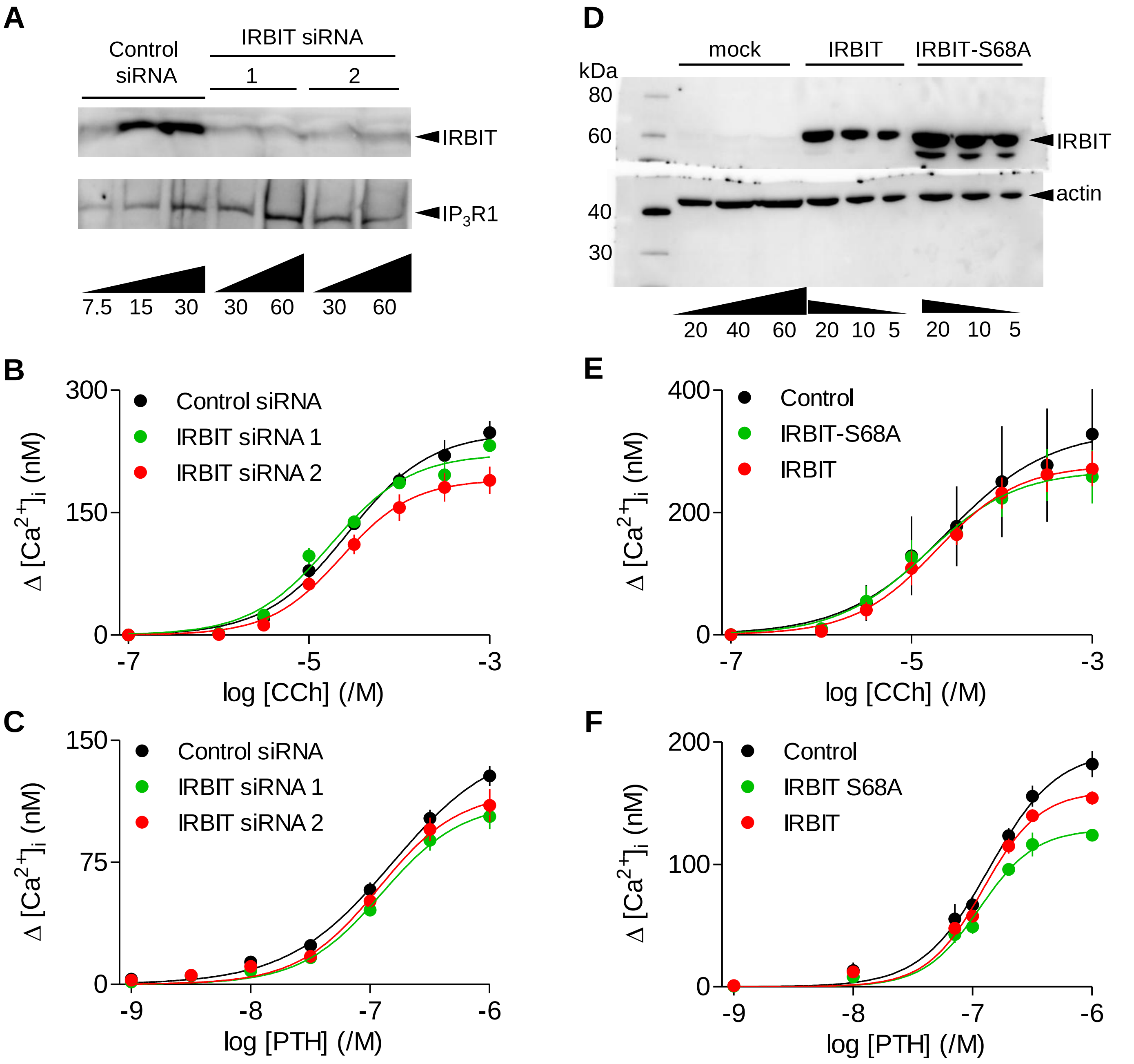

Figure S2. IRBIT Does Not Mediate the Effects of PTH on CCh-Evoked Ca^{2+} Release. Related to Figures 1-4.

(A) WB (taken from the same gel) show expression of IP_3R1 and IRBIT in HEK-PR1 cells transfected with control siRNA or 2 different siRNA against IRBIT. The amounts of protein loaded are shown beneath each lane (µg). Similar results (showing ~90% loss of IRBIT expression after treatment with either effective siRNA) were obtained in at least 3 WB. Neither the control siRNA nor mock transfection affected IRBIT expression. Expression of IP_3R1 was unaffected by IRBIT siRNA.
(B, C) Effects of siRNA treatment on CCh-evoked Ca^{2+} release (B) and the response to PTH added 2 min after CCh (1 mM) (C). Results (mean ± SEM, n = 3) show peak increases in $[Ca^{2+}]_i$ ($\Delta[Ca^{2+}]_i$).
(D) WB (taken from the same gel), typical of 3 similar WB, shows expression of β-actin, IRBIT and IRBIT-S68A in HEK-PR1 cells after transduction with baculovirus. Protein loadings (µg) are shown beneath each lane. M_r markers (kDa) are shown. Parallel transductions of cells with EGFP-IRBIT confirmed that >90% of cells expressed the protein.
(E, F) Effects of over-expressing IRBIT or IRBIT-S68A on CCh-evoked Ca^{2+} release (E) and the response to PTH added 2 min after CCh (1 mM) (F). Results are mean ± SEM, n = 3.

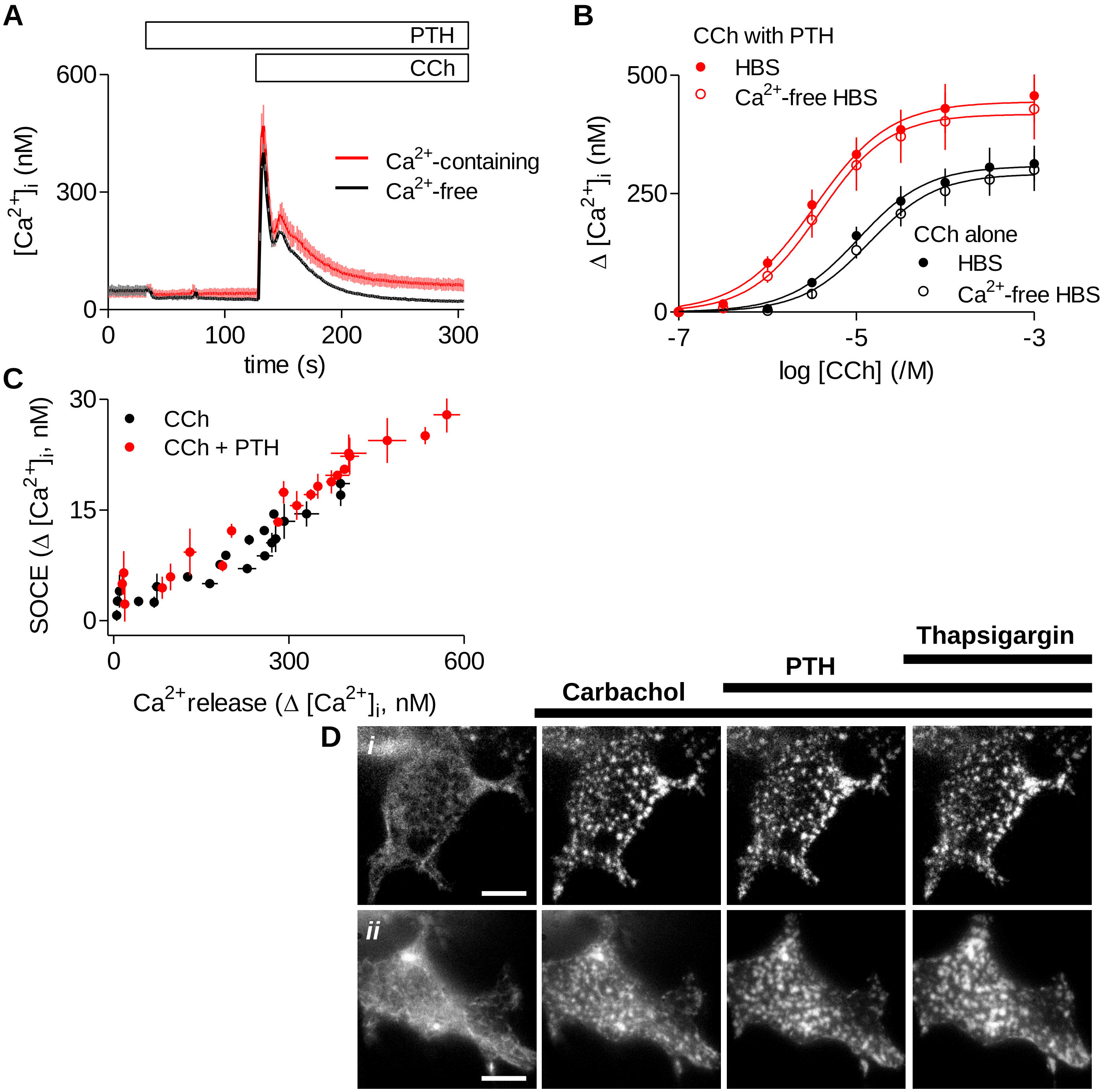

Figure S3. Store-Depletion Evoked by CCh or CCh With PTH Similarly Stimulate SOCE and Translocation of STIM1.
Related to Figures 1-4.

(A) Typical responses from HEK-PR1 cells stimulated with CCh after PTH (100 nM, 1 min) in either HBS or Ca^{2+}-free HBS. Mean ± SD, for 3 replicates for each trace.
(B) Summary results show the peak increase in $[Ca^{2+}]_i$ ($\Delta[Ca^{2+}]_i$).
(C) The sustained increase in $[Ca^{2+}]_i$ in normal HBS was determined 2.5 min after CCh addition (SOCE) and plotted against the initial peak increase in $[Ca^{2+}]_i$ (Ca^{2+} release) for each CCh concentration alone or with PTH. Results (B and C) show mean ± SEM, n = 3.
(D) Representative TIRFM images of HEK-PR1 cells expressing mCh-STIM1 and stimulated with carbachol (1 mM), PTH (100 nM) and then thapsigargin (1 µM) in Ca^{2+}-free HBS (*i*) or HBS (*ii*). Simultaneous recordings of $[Ca^{2+}]_i$ confirmed that each stimulus evoked the expected Ca^{2+} signal. However, the formation of STIM1 puncta varied considerably between cells. The results provided no clear evidence that PTH evoked formation of puncta that were spatially distinct from those evoked by carbachol. Scale bars = 10 µm.

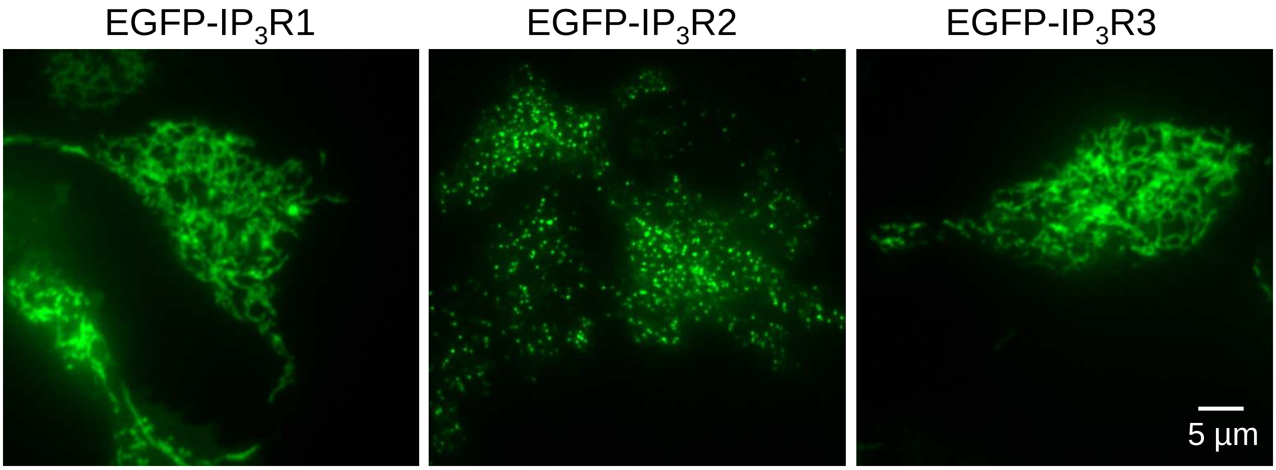

Figure S4. Distribution of GFP-tagged IP_3Rs in HEK-293 Cells.
Related to Figure 7.

Typical total internal reflection fluorescence (TIRF) images of HEK293 cells expressing EGFP-IP_3R1, EGFP-IP_3R2 or EGFP-IP_3R3.
